# From DNA-Encoded Library Screening to **AM-9747**: An MTA-Cooperative PRMT5 Inhibitor with Potent Oral In Vivo Efficacy

**DOI:** 10.1021/acs.jmedchem.4c03101

**Published:** 2025-03-18

**Authors:** Ian Sarvary, Mikkel Vestergaard, Loris Moretti, Jan Andersson, Jorge Peiró Cadahía, Sanne Cowland, Thomas Flagstad, Thomas Franch, Alex Gouliaev, Gitte Husemoen, Tomas Jacso, Titi Kronborg, Aleksandra Kuropatnicka, Anna Nadali, Mads Madsen, So̷ren Nielsen, David Pii, So̷ren Ryborg, Camillia Soede, Jennifer R. Allen, Matthew Bourbeau, Kexue Li, Qingyian Liu, Mei-Chu Lo, Franck Madoux, Narbe Mardirossian, Jodi Moriguchi, Rachel Ngo, Chi-Chi Peng, Liping Pettus, Nuria Tamayo, Paul Wang, Rajiv Kapoor, Brian Belmontes, Sean Caenepeel, Paul Hughes, Siyuan Liu, Katherine K. Slemmons, Yajing Yang, Fang Xie, Sudipa Ghimire-Rijal, Susmith Mukund, Sanne Glad

**Affiliations:** † Amgen Research, Amgen Inc, Ro̷nnegade 8, DK-2100 Copenhagen, Denmark; ‡ Amgen Research, Amgen Inc, Fruebjergvej 3, DK-2100 Copenhagen, Denmark; § Amgen Research, Amgen Inc, One Amgen Center Drive, Thousand Oaks, California 91320, United States; ∥ Amgen Research, Amgen Inc, 750 Gateway Blvd, South San Francisco, California 94080, United States; ⊥ Amgen Research, Syngene-Amgen Research and Development Center, Biocon Park, Bangalore 560099, India

## Abstract

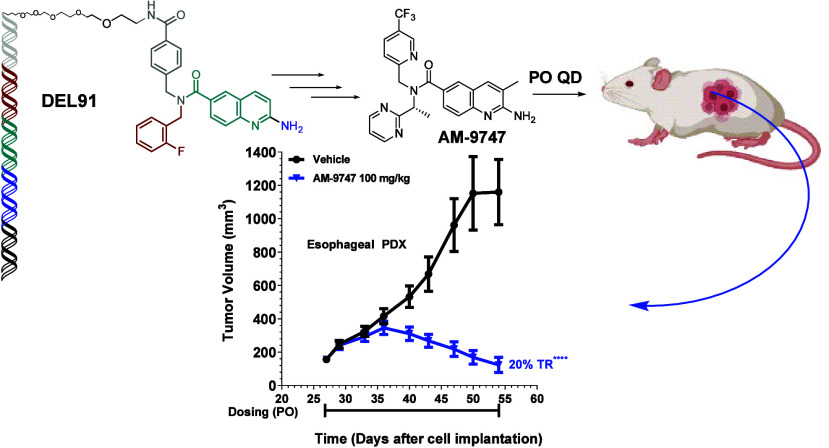

Inhibition of the methyltransferase enzyme PRMT5 by MTA accumulation is a vulnerability of MTAP-deleted cancers. Herein, we report the discovery and optimization of a quinolin-2-amine DEL hit that cooperatively binds PRMT5:MEP50 and MTA to generate a catalytically inhibited ternary complex. X-ray crystallography confirms quinolin-2-amine binding of PRMT5 glutamate-444, while simultaneously exhibiting a hydrophobic interaction with MTA. Lead optimization produced **AM-9747**, which selectively inhibits PRMT5-directed symmetric dimethylation of arginine residues of proteins, leading to a potent reduction of cell viability in MTAP-del cells compared to MTAP-WT cells. Once-daily oral dosing of **AM-9747** in mouse xenografts is well tolerated, displaying a robust and dose-dependent inhibition of symmetric dimethylation of arginine in MTAP-del tumor-xenografts and significant concomitant tumor growth inhibition without any significant effect on MTAP-WT tumor xenografts.

## Introduction

Protein arginine methyltransferase 5 (PRMT5) belongs to a group of nine protein arginine methyltransferases (PRMTs) that catalyze the methylation of protein arginine residues.^1−4^ PRMTs are classified into three categories, type I–III, based upon their catalytic specificity.^5^ Type I (PRMT1-4, PRMT6, and PRMT8) catalyzes arginine mono- and asymmetric dimethylation. Meanwhile, type II (PRMT5 and PRMT9) catalyzes arginine mono- and symmetric dimethylation, with PRMT5 as the primary enzyme for symmetric dimethylation of protein arginines.^6^ Lastly, type III (PRMT7) specifically catalyzes arginine monomethylation.

Endogenous PRMT5 forms a heterooctameric complex with methylosome protein 50 (MEP50). PRMT5 binds an arginine of a substrate protein and transfers a methyl group from the cosubstrate S-adenosylmethionine (SAM) to the targeted arginine.^7^ PRMT5 function is essential to mammalian cell survival with many cellular processes regulated through methylation, i.e., chromatin remodeling, gene expression, mRNA splicing, DNA replication, along with cell cycle regulation (by methylating spliceosomal Sm proteins, histones H2A, H3, and H4, nucleolin, SPT5, tumor suppressor p53, RAF proteins, and others).^8−12^ Notably, elevated PRMT5 expression levels in both clinical and preclinical studies have been observed in various cancers. Moreover, high expression of PRMT5 has also been associated with poor survival and has been correlated with tumor metastasis.^13−17^

**Figure 1:**
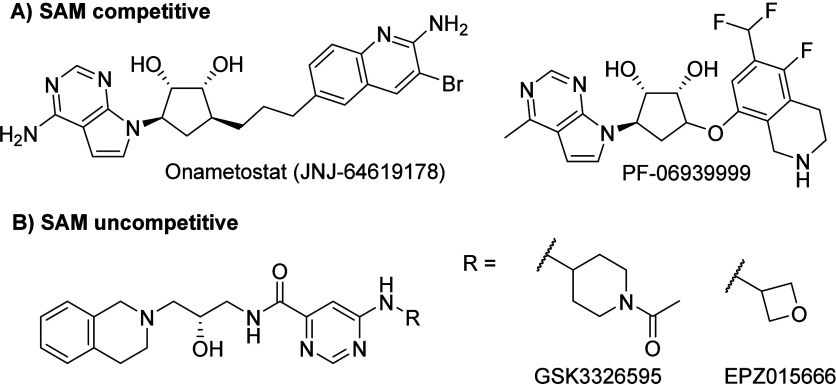
Compound structures of first generation PRMT5 inhibitors. (A) SAM competitive inhibitors Onametostat (JNJ-64619178) and PF-06939999. (B) SAM uncompetitive PRMT5 inhibitors GSK3326595 and EPZ015666.

First-generation PRMT5 inhibitors entering clinical trials were either SAM competitive (JNJ-64619178 and PF-06939999) or SAM uncompetitive (GSK3326595 and EPZ015666) (Figure 1).^18−21^ However, concerns regarding dose-limiting side effects such as anemia, thrombocytopenia, and neutropenia were apparent.^22,23^ Consequently, several PRMT5 ligands acting via different mechanisms (e.g., proteolysis targeting chimeras) are being investigated as potential cancer therapeutics.^12^

The cosubstrate SAM readily degrades into MTA, which in turn is a SAM-competitive inhibitor of PRMT5.^24^ Phosphorolysis of MTA, producing adenine and 5′-methylthioribose-1-phosphate, is catalyzed by MTAP and is a critical pathway for the regeneration of both adenine and methionine.^25^ In addition, the MTAP gene is located adjacent to the cyclin-dependent kinase inhibitor 2A (*CDKN2A*) gene, a tumor suppressor gene that is deleted in many forms of cancer. This homozygous deletion often occurs with the simultaneous loss of MTAP as a passenger gene, resulting in approximately 15% of all solid tumors being MTAP-deleted (MTAP-del).^8^

The absence of MTAP activity leads to an accumulation of cellular MTA resulting in partial inhibition of PRMT5. Therefore, an appealing strategy is to leverage this hypomorphic state and utilize MTA-cooperative inhibitors for targeting PRMT5. These would selectively inhibit the growth of MTAP-del cancer cells in contrast to MTAP-wild type cells (MTAP-WT) increasing the safety margin.^26,27^

**Figure 2:**
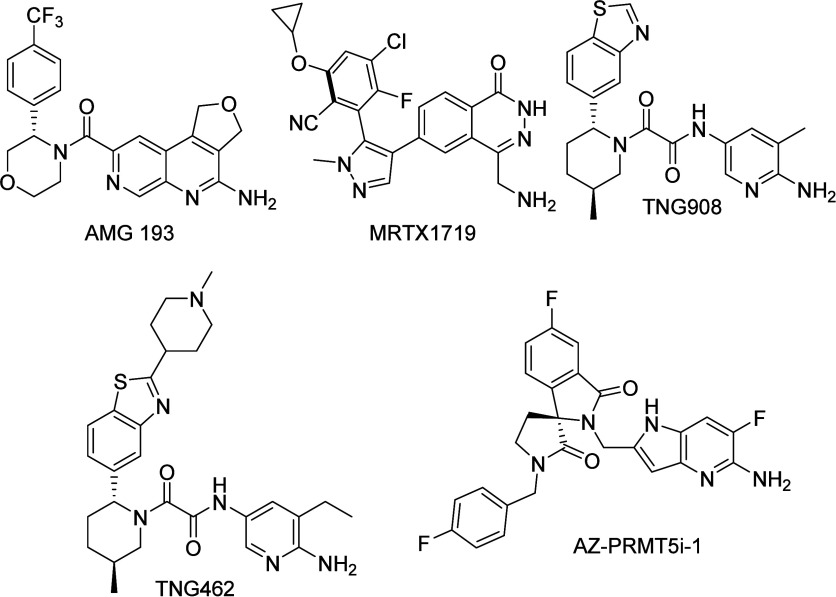
Structures of the MTA-cooperative PRMT5 inhibitors currently in clinical trials AMG 193, MRTX1719, TNG908, and TNG462. Additionally, AZ-PRMT5i-1 is shown as it is a close analogue to AZD-3470, which is also in clinical trials.

This vulnerability has been highlighted by numerous research reports.^9−11^ Consequently, a new generation of PRMT5 inhibitors employing MTA-cooperative PRMT5 binding has entered clinical trials for the treatment of patients with MTAP-del cancers AMG 193,^28^ MRTX1719,^29^ TNG908,^30^ and TNG462,^31^) along with AZD-3470^32^ (structure not shown), that are expected to give an increased therapeutic window arising from targeting the MTA:PRMT5 complex; Figure 2. Due to the absence of an officially published structure of AZD-3470, the close analog AZ-PRMT5i-1^33^ is depicted in Figure 2. A detailed overview of different PRMT5 inhibitors and their structures has recently been summarized.^12^

Herein, we report the identification of a quinolin-2-amine (Q2A) based hit series discovered as a side-product from a DNA-encoded library (DEL) in the course of screening the MTA preincubated PRMT5:MEP50 protein. The Q2A series demonstrated potent inhibition of PRMT5:MEP50 methyltransferase activity in the presence of MTA. During optimization, robust cell-based activity was achieved, creating a near-complete reduction (>80%) in symmetric dimethyl arginine (SDMA) leading to decreased cell viability of MTAP-del HCT116 cancer cells. The resulting lead, **AM-9747**, displayed a 75-fold difference in cell viability between HCT116 MTAP*-*del and -WT, thereby effectively exploiting differential MTA concentrations for the cooperative inhibition of PRMT5 and selectively targeting HCT116 MTAP-del cells.

Once-daily oral dosing of **AM-9747** demonstrated a reduction in the SDMA tumor biomarker, resulting in effective tumor growth inhibition and tumor regression when examined in various MTAP-del xenograft models. These results laid the foundation for our subsequent PRMT5 research, leading to **AMG 193**, the first MTA-cooperative PRMT5 for clinical evaluation, currently in Phase I/II clinical trials for the treatment of advanced MTAP-null solid tumors (NCT05094336 and NCT05975073).

## Results and Discussion

### DEL Screening and DEL91 Production

DNA-encoded libraries (DELs) have evolved into a powerful tool frequently used for small molecule hit discovery.^34^ Combinatorial (split and pool) DELs involve the synthesis of millions to billions of compounds covering substantial chemical space, with each individual final compound attached to a unique DNA coding sequence (tag). Following affinity selection, the DNA tags of enriched compounds allow deconvolution to the corresponding small molecule ligand structure, and the relative abundance of these DNA tags largely corresponds to the relative target binding affinity. Consequently, we often observe that DEL screening outputs provide valuable insights, forming a structure affinity relationship based upon the preliminary structures (DEL-SAFIR), which strengthens the validation.

**Scheme 1:**
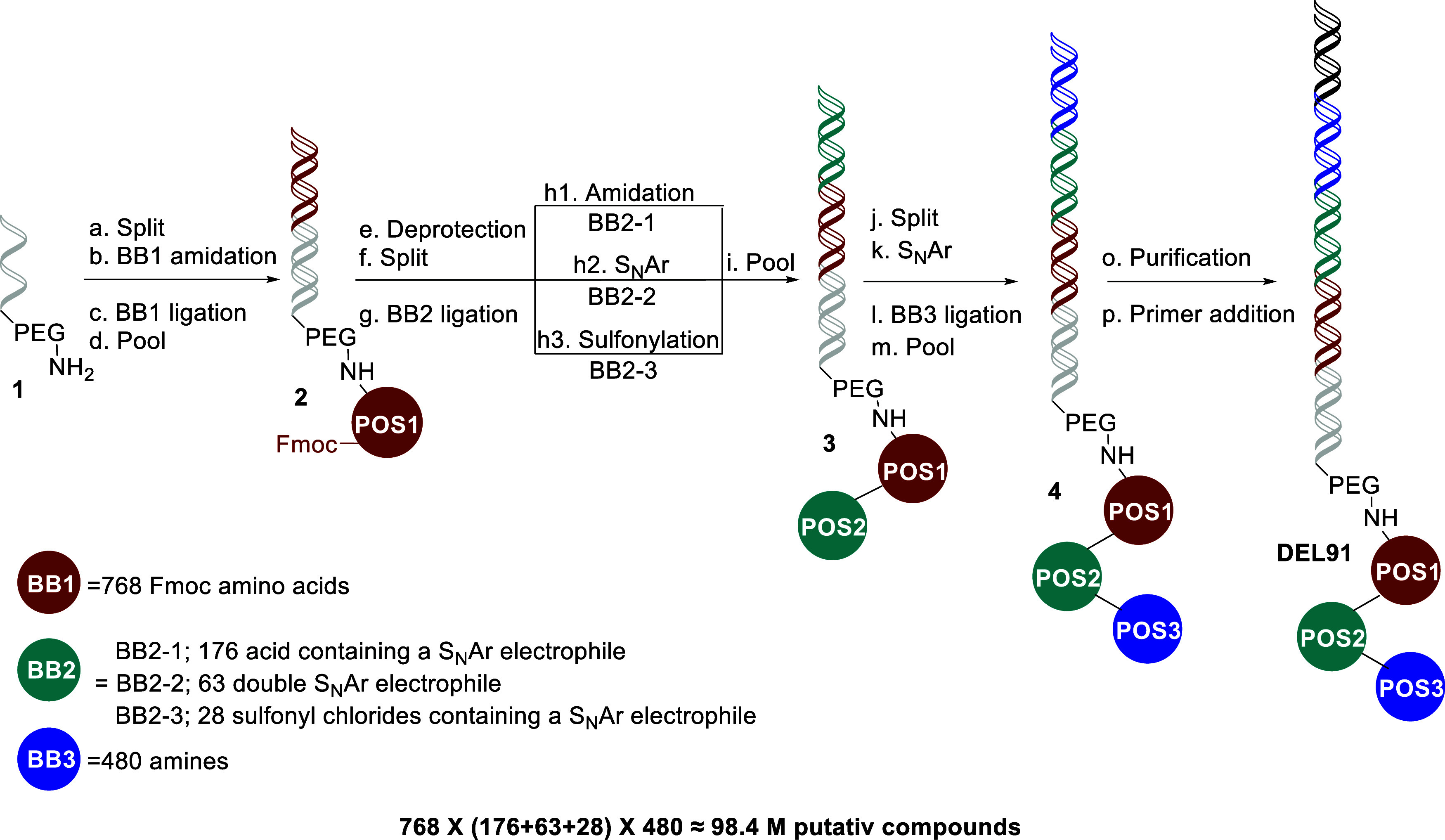
Production of DEL91 Reagents and conditions: (a) split into 768 wells; (b) **BB1**, DMTMM, water/DMSO, pH 8 (Na-phosphate buffer), RT, overnight; (c) BB1 DNA-tag, T4 DNA ligase, pH 7.5 (HEPES buffer), 37 °C to RT, inactivation at 80 °C; (d) the 768 wells were pooled; (e) piperidine, H_2_O, DMF, RT; (f) split into 262 wells; (g) BB2 DNA-tag, ligase, HEPES buffer, 37 °C to RT, inactivation at 80 °C; (h1) **BB2-1**, DMT-MM, water/DMSO, pH 8 (Na-phosphate buffer); (h2) **BB2-2**, water/DMSO, pH 8 (Na-phosphate buffer), 75 °C overnight; (h3) **BB2-3** water/THF, pH 9 (borate buffer), 30 °C overnight; (i) the 267 wells were pooled; (j) split into 480 wells; (k) **BB3**, water/DMSO, pH 8 (Na-phosphate buffer), 80 °C overnight; (l) BB3 DNA-tag, ligase, pH 7.5 (HEPES buffer), 37 °C to RT, inactivation at 80 °C; (m) the 480 wells were pooled; (o) gel electrophoresis; (p) **DEL91** specific primer extension.

When this work was conducted, there were no publicly available reports of MTA-cooperative PRMT5 inhibitors. To identify novel MTA-cooperative inhibitors, we envisioned that PRMT5 and MTA were required to preform the MTA+PRMT5:MEP50 complex prior to screening. Therefore, the DELs were screened with His-tagged PRMT5:MEP50 protein preincubated with MTA. The resulting complexes were captured on anti-His antibody beads, with subsequent bead washing and finally heat denaturation of the proteins. Applying this strategy, a single DEL, **DEL91**, produced several novel series of PRMT5 ligands. **DEL91** is a split and pool, linear, trimeric library, and the production is outlined in Scheme 1.

In the first cycle, position 1 (POS1) consisting of 768 fluorenyl methoxycarbonyl (Fmoc) protected amino acids (building block 1, **BB1**), was split into individual wells and attached to headpiece **1** via a (4-(4,6-dimethoxy-1,3,5-triazin-2-yl)-4-methylmorpholinium chloride (DMTMM) amide coupling. Subsequently, the DNA headpiece in each well was ligated to the corresponding **BB1**-tags and the 768 individual wells were pooled into **2**. For the second cycle, **2** was subjected to Fmoc-deprotection and split into 268 wells, followed by ligation with the corresponding POS2 **BB2**-tags, and employing the amine in either a DMTMM amide coupling, a S_N_Ar (nucleophilic aromatic substitution) reaction, or a sulfonylation, after which the library was again pooled producing **3**. The third cycle was initiated by splitting the library into 480 wells. Position 3 **BB3** was introduced via a S_N_Ar reaction and the **BB3**-tag by subsequent ligation in individual wells. Pooling the wells produced **4**. Finally, the addition of a library-specific primer finalized **DEL91** with a total of 98.4 million preliminary DEL molecules.

**Figure 3:**
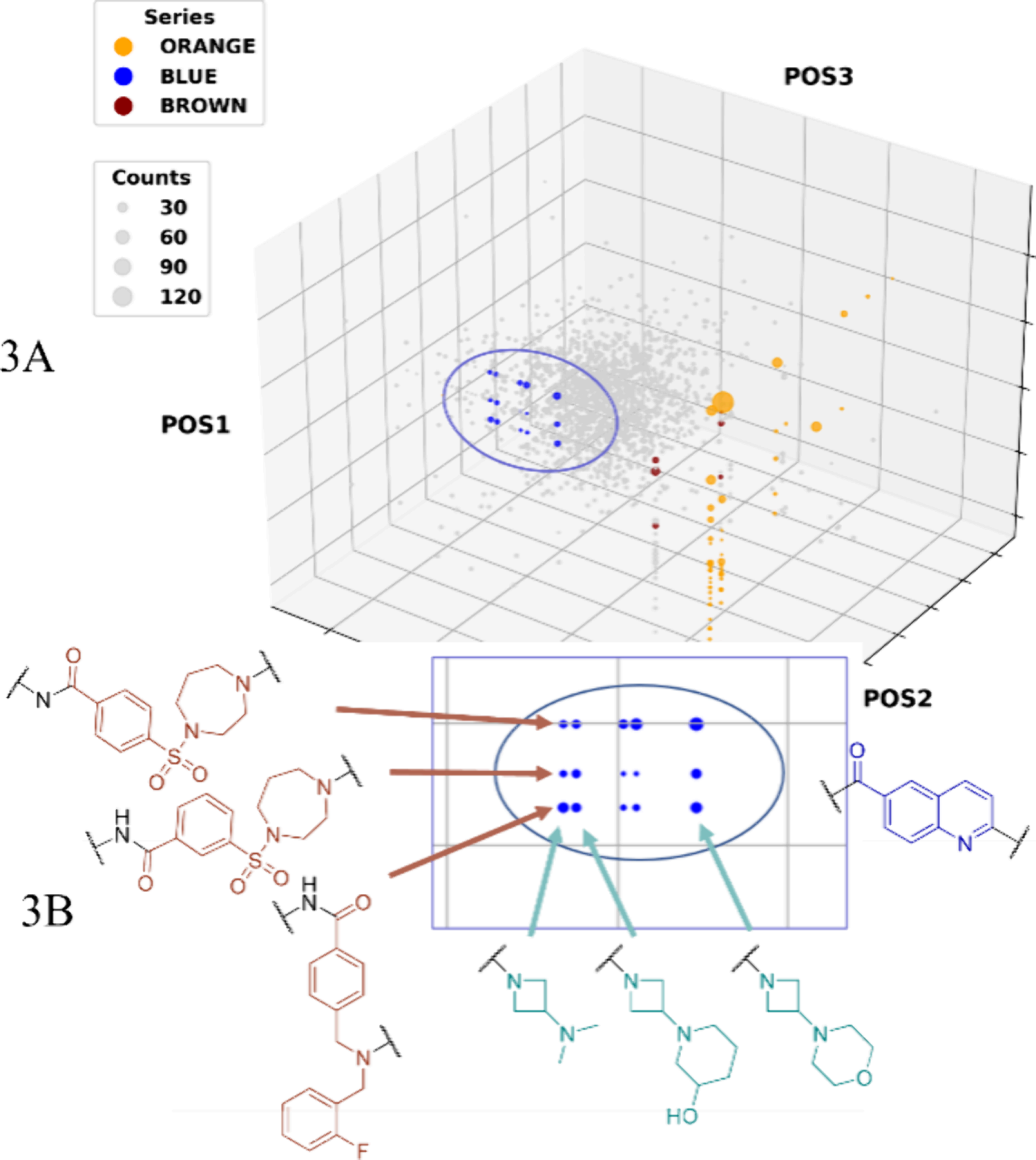
(A) **DEL91** PRMT5:MTA affinity selection output represented as a 3D scatterplot, with the **BB**s of the structures represented on the *x*, *y*, and *z* axis, and the size of the circle representing the counts. Background displayed in light gray and three series ORANGE, BROWN, and BLUE represented in their respective colors and the BLUE series encircled. (B) Transformation of the 3D to a 2D plot by locking POS2. POS1 represented on the *y* axis and POS3 on the *x* axis. The preliminary ligand structures of the BLUE series with the two POS1-subseries in brown (diazepane-sulfonyl-phenyl and the substituted dibenzyl), the POS2 locked as the blue structure, and the POS3 in green, outlining the DEL-SAFIR.

The DNA-tags from the lligands obtained from affinity selection against MTA preincubated PRMT5:MEP50 were amplified by PCR and after DNA sequencing, the corresponding preliminary ligand structures were obtained. Finally, the preliminary ligands were ranked according to their enrichment in the selection output and subsequently clustered into chemical series utilizing structural resemblance. Three novel DEL-based PRMT5 ligand series were identified from screening, depicted as ORANGE, BROWN, and BLUE, Figure 3A. A more comprehensive account of the screening process will be communicated in due course.^35^

The vast majority of selection outputs exhibit a clear one-to-one correlation between the unique DNA tag and its corresponding ligand. Ranking of the preliminary compounds, within different series, creates a preliminary structure affinity relationship (DEL-SAFIR) of the series. In addition to potentially gaining early SAFIR learnings, a clear DEL-SAFIR further builds confidence in the DEL-selection output and assurance of realization of the combinatorial DEL-library.

As a rule, DEL-SAFIRs of trimeric ligands will be founded upon the structure of the POS3 (alternatively the POS2-POS3 dimer), with the hits of the series creating vertical lines such as with the ORANGE series, Figure 3A. However, the BLUE series was based around a single POS2 (originating from the **BB2** 2-chloroquinoline-6-carboxylic acid), thus creating horizontal lines, blue dots Figure 3A. Locking the POS2 to this moiety transformed the 3D box scatter plot into a 2D representation, illustrated in Figure 3B. The DEL-SAFIR of the BLUE series generated two subseries derived from the POS1 origin: diazepane-sulfonyl-phenyl (Figure 3B left top and middle) or a substituted dibenzyl amine (Figure 3B bottom left). Finally, POS3 was an azetidine substituted with a tertiary amine in multiple constellations, i.e., piperidine, dimethylamine, and morpholine (Figure 3B green).

### Inhibitor Validation

Initial off-DNA synthesis of the DEL ligands from the **DEL91** selections was performed on the solid phase. To align closely with library production, the same synthetic sequence and batches of the BBs used for **DEL91** production were employed. However, the aqueous milieu, different reagents, and the substantial excess of building blocks (>200 fold) in DEL production may lead to distinct reactivity and formation of unexpected side-products, which in turn may act as binders. Despite taking measures when resynthesizing library hits, variations in reactivity and generation of side products may differ from library production. Synthesis on Rink-amide produced the corresponding 1°-amide, representing the 2°-amide present in the library.

**Figure 4:**
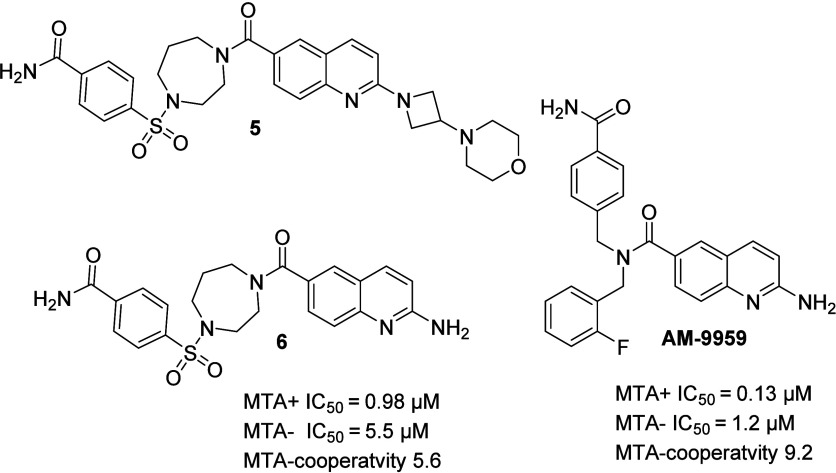
Structure of the preliminary MTA cooperative PRMT5 ligand **5** and the corresponding validated MTA-cooperative inhibitor **6** and the substituted dibenzyl subseries **AM-9959**. MDCKII-MDR1 and -BCRP permeability were performed according to Method B.

After being released from the resin, the crude compounds were purified by UPLC, characterized via time-of-flight (TOF) high-resolution mass spectroscopy (HRMS), and finally quantified by chemiluminescence nitrogen detection (CNLD). Thereafter, these compounds were examined in two methyltransferase assays, MTase-Glo, containing either 5 μM added MTA (MTA+) or no added MTA (MTA-), validating the compounds as PRMT5 ligands and examining their MTA cooperative inhibitory properties. The MTA-cooperativity was determined through the ratio of these assays (MTA- IC_50_/MTA+ IC_50_). Material from the solid-phase synthesis of the most enriched preligand **5;**Figure 4, displayed a modest inhibition in the MTA+ assay and no activity in the MTA- assay. Upscaling of **5** with a solution-phase synthesis produced additional material for further analysis. However, the material from solution phase synthesis was inactive in both biochemical assays.

When considering that the DEL-SAFIR was based upon the POS2, the modest inhibition of material from the solid phase, in combination with the lack of biochemical inhibition of the solution phase material **5**-solution, led us to hypothesize that the actual ligand behind the inhibition was in fact a side-product.

A ligand-specific DNA tag represents all products formed during library production of the ligand’s reaction sequence (including unreacted starting materials, deletion-, by-, and side-products).^36,37^ Further, applying PCR and high throughput DNA sequencing generates a most sensitive selection output, even described as ultrasensitive.^38^ Reanalysis of the material from the solid-phase synthesis, **5**-solid, revealed an impurity of ca. 10% (see the Supporting Information).

The mass of this impurity corresponded to **6**; Figure 4. Solution phase synthesis and subsequent examination of **6** in the MTase-Glo assays showed it had robust inhibition in both assays (MTA+ IC_50_ = 0.98 μM and MTA- IC_50_ = 5.5 μM, Figure 4), validating **6** as an MTA-cooperative inhibitor of PRMT5.

However, even more encouraging was that dibenzyl subseries analogue **AM-9959** displayed better activity and MTA-cooperativity in the biochemical assays (MTA+ IC_50_ = 0.13 μM and 9-fold cooperativity). Pleasingly, **AM-9959** was a promising starting point from both medicinal and synthetic chemistry perspectives.

### LHS (Left Hand Side) Optimization

Contrasting ligand validation or confirmation of DEL-selection outputs, we often experience that 1°-amides impede further development due to potential drawbacks, such as 1°-amide hydrolysis, decreased passive cell permeability, or increased efflux. Therefore, **AM-9959** was examined in Madin-Darby Canine Kidney cell trans-well assays, where cells were transfected with either multi-drug resistance gene 1 (MDCKII-MDR1) or breast cancer resistant protein (MDCKII-BCRP). Apical to basal (A-B) and basal to apical (B-A) rates (Papp) were measured and efflux ratios (B-A/A-B) were calculated. Values indicating high permeability were defined as Papp >4 μcm/s, likewise low efflux was defined as <4. Analysis of **AM-9959** in MDCKII-MDR1 and -BCRP for permeability displayed a poor A-B Papp for MDR1 and a modest A-B Papp for BCRP (0.3 and 1.2 μ cm/s, respectively) along with significant efflux in both assays.

This identified **AM-9959** as a possible substrate for two of the three relevant ATP-binding cassette transporters (ABC) that cause multidrug resistance in cancer, ABCB1 (MDR1 or P-gp) and ABCG2 (BCRP). High efflux can provide challenges for drug development such as increased risk for drug–drug interactions, reduced permeability, lower intestinal uptake, increased renal and hepatic clearance, impaired CNS exposure, and a negative impact upon cancer treatment.^39^

To ameliorate efflux, the LHS-up 4-carboxamide was replaced with electron-withdrawing groups (EWG) such as −Br or −CF_3_ to reduce polar surface area (PSA) and to eliminate the strong 1°-amide H-bond capabilities while inducing a similar polarization of the attached phenylene. Gratifyingly, these EWG modifications maintained or increased MTA+ potency (**7**, IC_50_ = 130 nM and **8**, IC_50_ = 60 nM) along with increased MTA-cooperativity for the latter; Table 1.

**Table 1:**
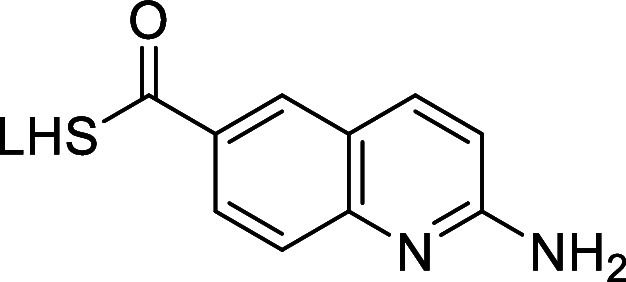

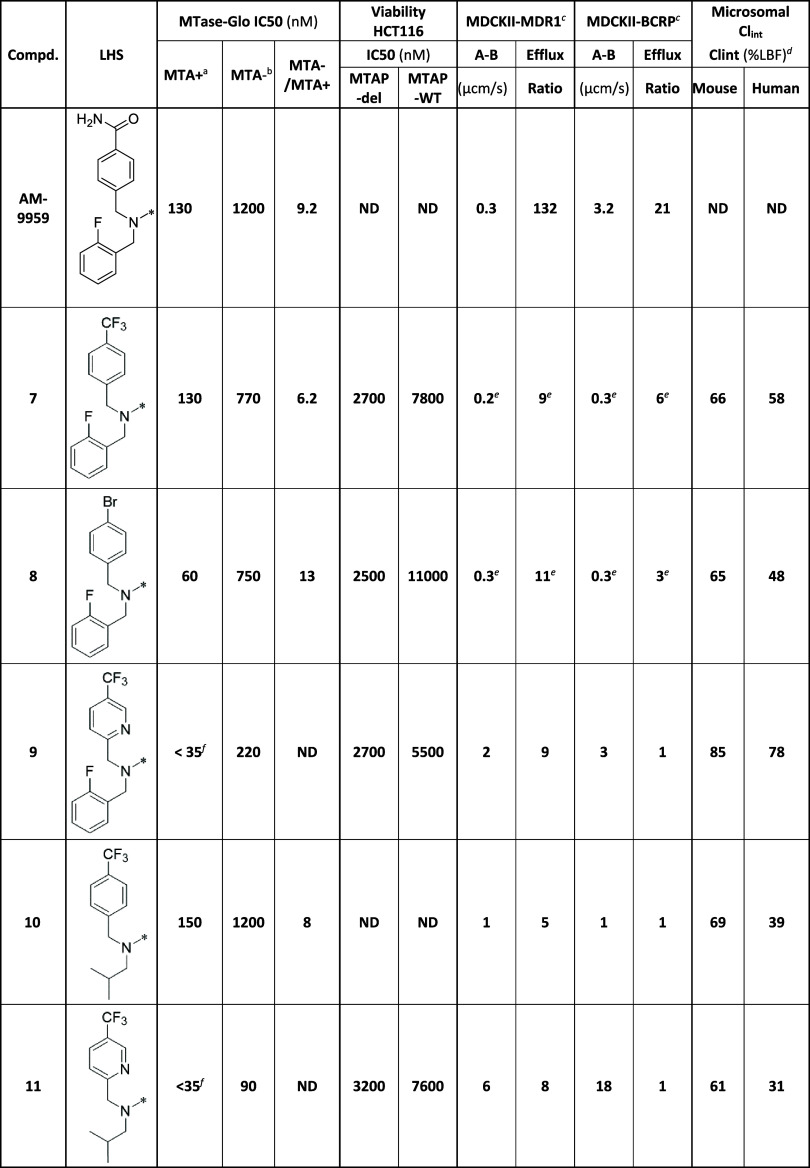
Initial SAR of the LHS^g^

a 5 μM MTA.

b 0 μM MTA.

c MDCKII-MDR1 and -BCRP membrane permeability at 1 μM substrate concentration with BSA, Method B.

d Intrinsic clearance was obtained from scaling in vitro half-lives in liver microsomes to the corresponding %LBF of the species.

e Poor recovery.

f Poor curve fitting near, or below, theoretical assay limit and therefore reported as <35 nM.

g All data represent *n* ≥ 2.

Introduction of a CF_3_-pyridinyl instead of a CF_3_-phenyl as the LHS-up aromatic moiety identified **9** with ameliorated efflux combined with MTA+ inhibition at low ligand concentrations. Investigating the LHS-down revealed that an isobutyl group (**10**) possessed similar potency as the 2-F benzyl but with an improved MTA cooperativity. Combining the CF_3_-pyridyl LHS-up and the isobutyl LHS-down, **11**, resulted in a potent inhibitor in both MTA+ and MTA- assays (IC50 < 35 and 90 nM, respectively). In addition, the compound showed better permeability with lower efflux in both MDCKII assays. Compounds were thereafter evaluated in CellTiter-Glo assays, employing HCT116 colon carcinoma MTAP-WT cells and the corresponding MTAP-del isogenic clone. Cell viability was determined after 6 days in both cell lines, and the IC_50_ values were used to determine their activity in the different cell lines. The cellular MTA-selectivity was defined as the ratio of these values (MTAP-WT IC_50_/ MTAP-del IC_50_).

However, the biochemical potency in Table 1 is not reflected in the viability assays. For instance, **11** and **7** displayed similar potency in the viability assays (MTAP-del IC_50_ = 3.2 and 2.7 μM, respectively), whereas the former was below our assay limit in the biochemical MTA+ assay, while the latter was moderately active. Further, **11** was 100-fold less potent in the cell viability assay compared to the corresponding biochemical assay (MTAP-del IC_50_= 3.2 μM, relative to MTA+ IC_50_ < 35 nM). Even if **11** did not display the same efflux for ABC transporters as **AM-9959** (ABCB1 and ABCG2) other ABC transporters, permeability, or a plethora of other mechanisms may influence intracellular concentrations or the inhibitor-PRMT5+MTA ternary complex in cells, which in turn may impact viability assays. Also, even if a biochemical IC_50_ and a cellular IC_50_ are not expected to perfectly correlate, we had concerns about how this would affect further optimization and structure–activity relationship (SAR) exploration.

Compounds were assessed for mouse and human in vitro intrinsic clearance in liver microsomes and reported with predicted in vivo hepatic clearance (CL) as the calculated percentage liver blood flow (%LBF) and defined by the following criteria: low CL < 30%LBF, moderate CL = 30–70%LBF, and high CL > 70%LBF (see Supporting Information). The 1°-amide substitution to an EWG (**AM-9959** to **7**, **8,** and **9**) decreased the predicted metabolic stability for mouse and especially human microsomes. The increase of the predicted clearance with the 2F-benzyl LHS-down (**7** and **9**) was slightly improved with the LHS-down iso-butyl (**10** and **11**), but not to the point as to classifying the compounds as having low predicted hepatic clearance.

### RHS (Right Hand Side) Exploration

**Figure 5:**
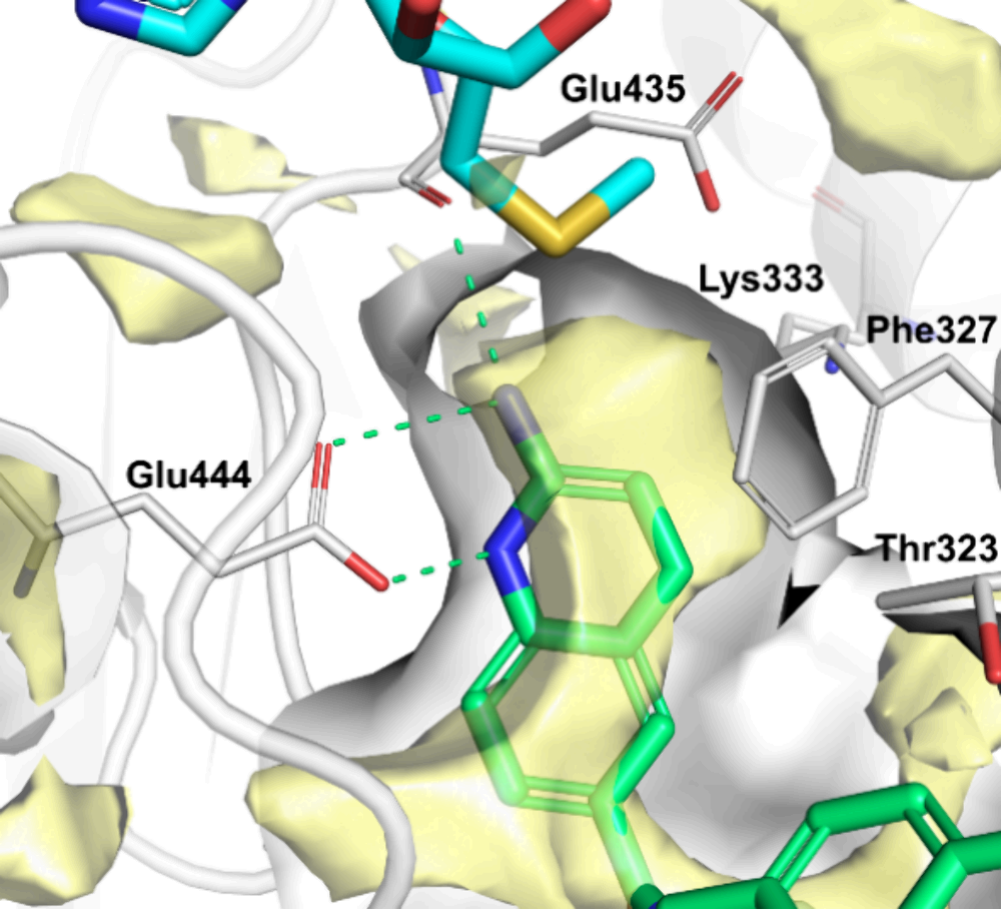
Starting point coordinates were obtained from 5FA5 in PDB. Docking of **7** (green) in the binding site of PRMT5 with MTA (cyan), PRMT5 is depicted as ribbons with key residues of the binding site represented as sticks and labeled. The binding-site internal surface is light gray, and favorable energy grid points for hydrophobic interactions are rendered as yellow surfaces. Main Q2A polar interactions are shown as green dashed lines. NB The yellow surface, displaying favorable points for hydrophobic interactions, influences the color of the aniline nitrogen, rendering it gray instead of blue.

For an improved understanding of the Q2A series binding and exploring possible advantageous structural improvements, we turned to modeling of **7** (see the Supporting Information). The docking model of **7** shows protonated Q2A as the main feature of the binding, forming a salt bridge to Glu444, an H-bond to the carbonyl of Glu435, and several hydrophobic interactions of surrounding residues; Figure 5.

To further investigate and potentiate ligand binding, the protein region around the ligand was explored by using an energy-grid approach for predicting favorable hydrophobic contacts within the RHS-pocket hydrophobic binding site. This permitted us to envisage modifications of the ligands to obtain additional favorable interactions. Primarily, introducing small alkyl groups, such as methyl, in position 3-, or 7- of the Q2A seemed favorable. However, it could be argued that such modifications could possibly be accepted at other positions as well, such as the 4- position. Therefore, a methyl scan was performed on the RHS, synthesizing the compounds with a methyl group at each available position of the Q2A RHS.

The introduction of a methyl group at either the 8-, 7-, or 5-postions did not increase the potency or selectivity in the biochemical and were all >5 uM in the MTAP-del viability assays (data not shown). However, in agreement with our modeling, the introduction of a methyl group at either the 4- or 3- positions (**12** and **AM-9934,**Table 2) increased the inhibitory effect. The IC_50_ values of these were now below the assay limit of the MTA+ assay and approaching the limit of the MTA- assay as well (IC_50_ = 76 and 37 nM, respectively).

**Table 2:**
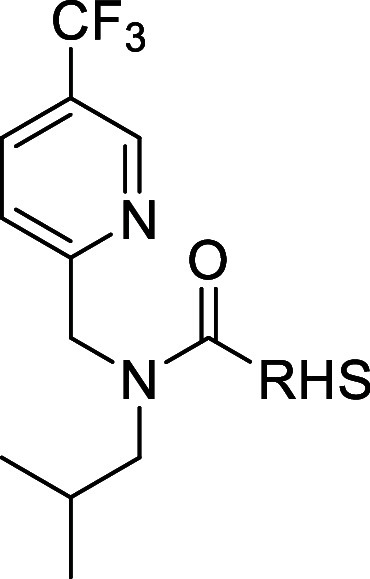

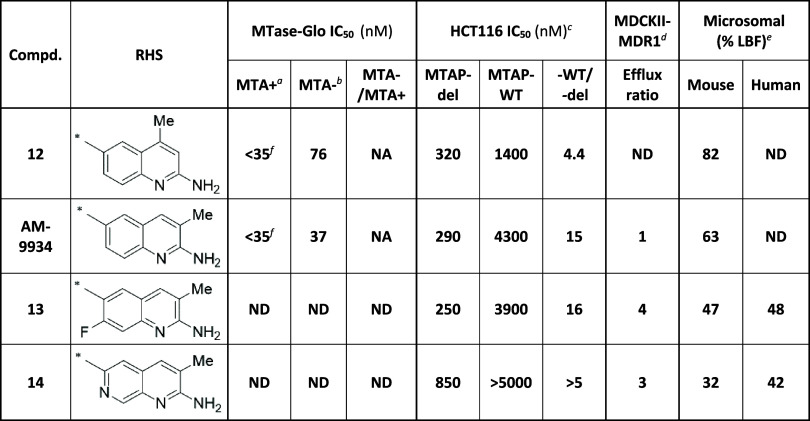
Right Hand Side (RHS) Investigations^g^

a 5 μM MTA.

b 0 μM MTA.

c Inhibition of viability evaluated on day 6 by the luminescence-based assay in HCT116 MTAP-del and MTAP-WT cells.

d MDCKII-MDR1 membrane permeability at 1 μM substrate concentration, efflux ratio A-B/B-A, Method A.

e Intrinsic clearance obtained from scaling in vitro half-lives from microsomes and based upon %LBF.

f Poor curve fitting below or near the theoretical assay limit and therefore reported as <35 nM.

g All data represent *n* ≥ 2.

Importantly, a methyl introduced in either the 4- or 3-position (**12** and **AM-9934**) for the first time resulted in compounds with viability IC_50_ values for the MTAP-del cells below 1 μM (IC_50_ = 320 and 290 nM, respectively). Furthermore, the MTAP-WT IC_50_s of **12** and **AM-9934** were 1.4 and 4.4 μM, respectively, corresponding to a 15-fold MTA selectivity for the latter. The compounds from the methyl scan (**12** and **AM-9934**) did not significantly alter the permeability or efflux in MDCKII-MDR1 cells compared to **11**. However, the metabolic stability of **13** was predicted to be low (82%LBF) in mouse microsomes.

Encouraged by the increased inhibitory potency of **AM-9934**, we synthesized and evaluated analogues of the methyl group. An H to F substitution or replacing methyl with small cyclic moieties such as cyclopropyl or -butyl has been used extensively to enhance both metabolic stability and biological activity.^40−42^ However, increasing the size of the 3-position substituent from methyl to ethyl, isobutyl, cyclopropyl, or cyclobutyl reduced the cellular inhibition by these compounds without increasing metabolic stability. Further, substituting hydrogen to fluorine on the methyl of the 3-methyl-Q2A (from CH_3_ to CFH_2_) created a compound with an inherent metabolic instability.

A subsequent fluorine scan of **AM-9934** revealed that the H could be substituted with a 7- F analogue, **13**, with a similar profile to **AM-9934** regarding potency, MTA-cooperativity, and metabolic stability. In addition, a corresponding N-scan (substituting a CH to an N in the quinoline aromatic system) of **AM-9934** also highlighted the 7-position and **14** displaying a slightly increased predicted metabolic stability, albeit with a 3-fold lower potency in the MTAP-del viability assay.

### LHS-Down Exploration

Both **12** and **AM-9934** displayed activities below the theoretical IC_50_ assay limit of the MTA+ assay and were approaching the theoretical IC_50_ limits of the MTA- assay as well. Consequently, hereafter, we focused on evaluating inhibition in the cell viability assays. Compared to the initial validated ligands (**6** and **AM-9959**), progress had been achieved in addressing efflux and enhancing cellular PRMT5 inhibition. However, the predicted hepatic clearance was not optimal. Despite the increased cellular potency of **AM-9934**, the molecule did not meet the desired criteria for further exploration in vivo. We reasoned that increased focus on the LHS-down could further improve cellular potency or metabolic stability; Table 3.

**Table 3:**
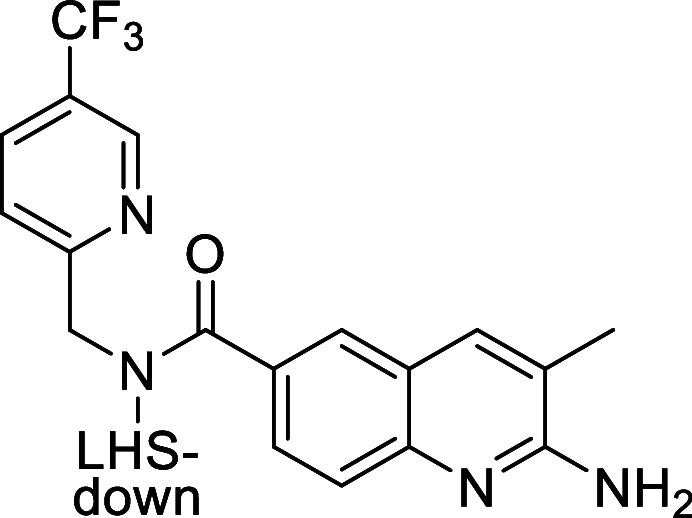

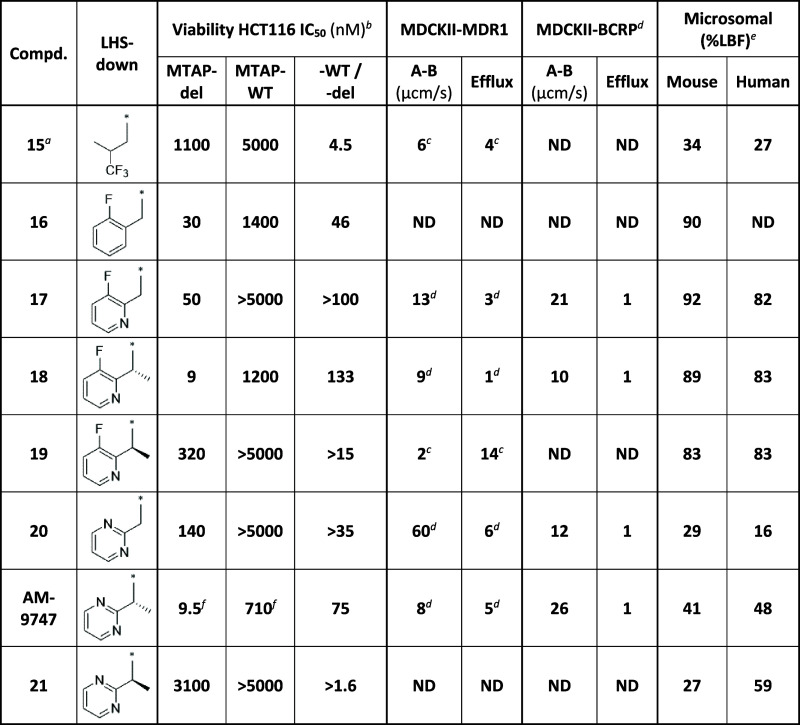
LHS-Down Exploration^g^

a Most potent enantiomer, configuration not determined.

b Inhibition of viability evaluated on day 6 by the luminescence-based assay in HCT116 MTAP-del and MTAP-WT cells.

c MDCKII-MDR1 membrane permeability at 1 μM substrate concentration, efflux ratio A-B/B-A, Method B.

d MDCKII-MDR1 and -BCRP MDCKII-MDR1 membrane permeability at 1 μM substrate concentration, efflux ratio A-B/B-A, Method B.

e Intrinsic clearance obtained from scaling in vitro half-lives from pooled microsomes and based upon %LBF.

f Average of *n* = 8, Standard Deviation: HCT116 MTAP-del ± 2.7 nM and *n* = 6 HCT116 MTAP-WT ± 0.52 μM.

g All data represent *n* ≥ 2.

To improve metabolic stability, various aliphatic LHS-down compounds were investigated, such as **15**, whereas its enantiomer (not shown) was less potent. These modifications improved predicted metabolic stability in human microsomes, classifying them as low clearance compounds in human microsomes (27%LBF). However, in mouse microsomes, these were still predicted to have medium intrinsic clearance (34%LBF). Moreover, this slight improvement came at the expense of decreased cellular potency, relative to **AM-9934**. Reintroducing the original aromatic ortho-fluoro benzyl LHS-down from **DEL91** in **16** produced a 10-fold increase in cellular potency in HCT116 MTAP-del cells compared to **AM-9934** (IC_50_ = 30 and 290 nM, respectively). Further, the cellular MTA-selectivity was boosted to 46-fold.

Therefore, we conducted LHS-down SAR exploration of aromatic moieties, e.g. producing **17**, with a similar cellular potency (IC_50_ = 50 nM) but demonstrating excellent cellular MTA-selectivity (>100-fold, MTAP-WT IC_50_ = > 5 μM). The introduction of an alpha-methyl group further improved cellular potency in the MTAP-del viability assay for the R-enantiomer (**18**), while the S-enantiomer (**19**) showed reduced potency (IC_50_ = 9 and 320 nM, respectively). Additionally, the cellular MTA selectivity of **18** was exquisite (133-fold). However, the hepatic clearance of the compounds with the ortho-fluoro-phenyl or -pyridyl in the LHS-down (**16**, **17**, **18,** and **19**) were all predicted to be high by both human and mouse microsomes.

Further exploration of heteroaromatic LHS-down motifs yielded **20**, featuring a pyridinyl in this position. This compound displayed MTAP-del IC_50_ = 140 nM and a cellular MTA-selectivity >35-fold. Further, it displayed low clearance in both human and mouse microsomes. Encouraged by these findings, a methyl group was introduced to the benzylic position on the LHS-down to investigate the potential enhancement of cellular inhibition. Consistent with previous observations, the R-enantiomer, **AM-9747**, demonstrated excellent cellular potency (HCT116 MTAP-del IC_50_ = 9.5 nM), whereas the S-enantiomer, **21**, exhibited a weaker inhibitory effect. The former also displayed excellent cellular MTA-selectivity (75-fold, MTAP-WT IC_50_ = 710 nM). Viability data varies slightly from our previously published results,^43^ due to minor differences in the assay setup between studies. The metabolic stability of **AM-9747** was predicted to be moderate (41%LBF), however, with good permeability and relatively low efflux in the MDCKII-MDR1 assay (PappA-B = 8 μcm/s and 5, respectively).

### Binding Mode Description from X-ray

**Figure 6:**
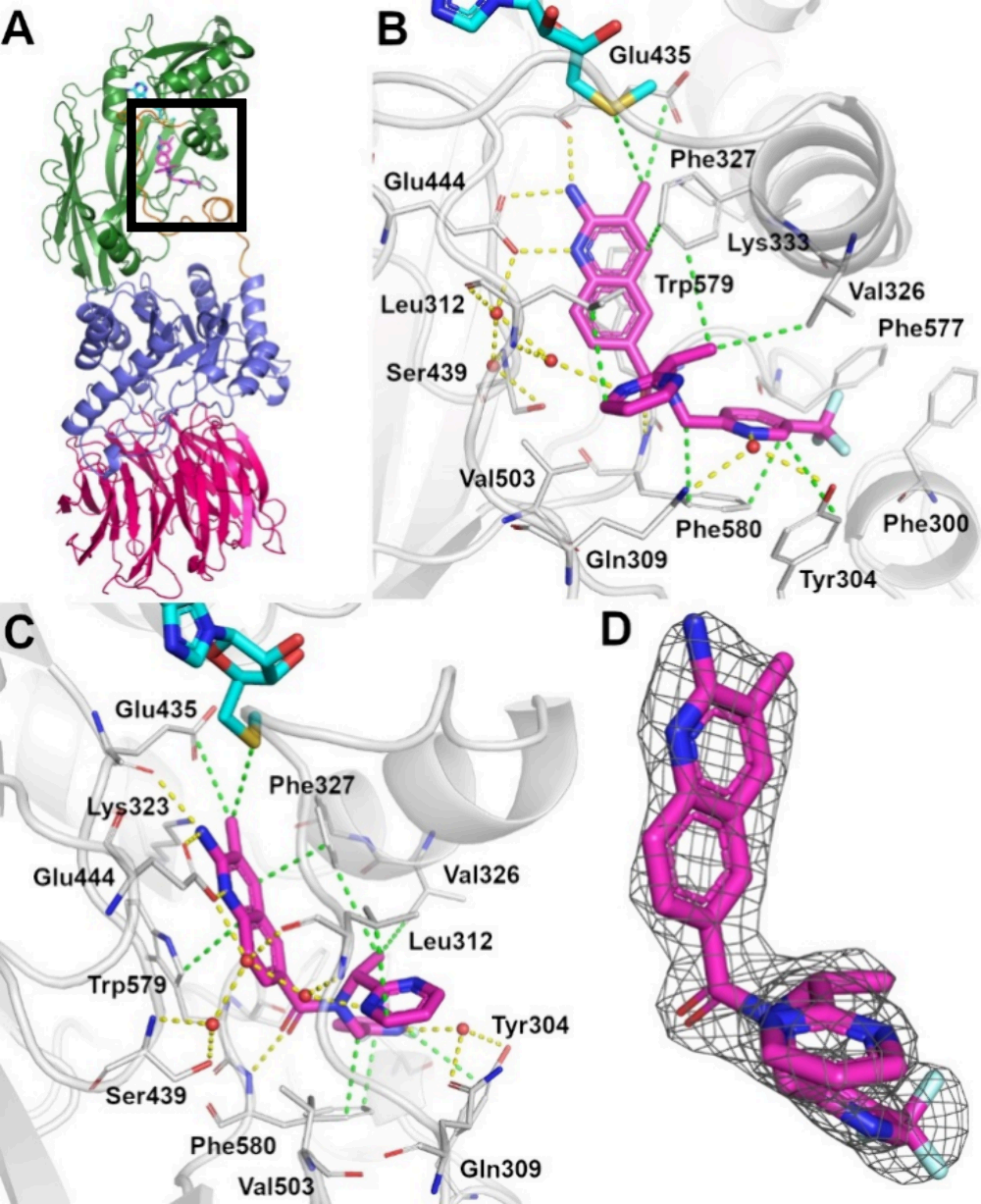
Crystallographic complex of **AM-9747** and MEP50:PRMT5 with MTA, PDB ID: 9MRE. (A) Proteins, MEP50 and PRMT5, have their secondary structure represented and are color-coded for MEP50 (warm pink), PRMT5 catalytic domain (green), and the TIM barrel domain (violet), and the loop linking these two domains (orange). The ligands, MTA (cyan) and for **AM-9747** (magenta), are represented as sticks. (B,C) Coordinates of the crystallographic structure are represented to reveal the binding mode details of **AM-9747**. The secondary structure of the protein is depicted and colored gray. Residues in proximity of the ligand are drawn as sticks with gray carbon atoms. **AM-9747** and MTA are also in stick representation, and carbon atoms are colored magenta and cyan, respectively. Polar interactions are depicted as yellow dashed lines and nonpolar interaction are in green dashed lines. (D) **AM-9747** is depicted as sticks with magenta carbon atoms, and the 2Fo-Fc electron density map for the ligand is also shown as mesh at 2.0 RMSD, clearly depicting the R-enantiomer.

The binding mode of **AM-9747** was obtained from the cocrystal structure of the ternary complex of PRMT5:MEP50, MTA, and the ligand. The overall complex has the classic structure as previously observed^7^ with the PRMT5 catalytic domain in contact with the N-terminal domain (TIM barrel), which is in contact with MEP50, Figure 6A. **AM-9747** resides within the peptide binding site at the position occupied by the arginine substrate and in the proximity of the coinhibitor MTA (see Figure 6B,C). The 2-aminoquinoline appears to be the driving moiety for the binding of this chemical series, featuring a salt bridge to Glu444 as the main interaction with PRMT5. The Q2A also forms a hydrogen bond with the carbonyl backbone of Glu435, and the quinoline ring system is sandwiched by Phe327 and Trp579. The methyl group at position 3 exhibits contact with Glu435 and more interestingly with the sulfur of the coinhibitor MTA, further substantiating the hydrophobic interactions for this portion of the ligand. The interaction with the sulfur supports the MTA-cooperativity observed for PRMT5 inhibition of **AM-9747**.

Another hydrogen bond is observed between the secondary amide of **AM-9747** and the main chain nitrogen of Phe580. In addition, two water-mediated interactions were also observed, one between the nitrogen atom of the para-CF_3_ pyridine and Gln309 together with Tyr304 and the other as part of a network with three water molecules starting from the nitrogen atom of the pyrimidine LHS-down and connecting with Leu312, Ser439, and Glu444. Finally, the crystallographic complex reveals hydrophobic contacts from the ligand pyrimidine ring packed against side chains of residues Leu312 and Val503, while the para-CF_3_ pyridine moiety shows hydrophobic packing with Phe300, Tyr304, Phe580, and Phe577.

Further examination of the crystallographic structure of **AM-9747** (Figure 6) revealed the importance of LHS-down chirality. The S configuration of **21** would orient the LHS-down Me-group toward the aminoquinolinone and eliminate the Phe327 and Val326 hydrophobic interactions. In addition, this drives the pyrimidine into a different position, further disrupting the positive interactions in the region. Clearly, **21** cannot establish the binding strength observed with **AM-9747** or even **20**.

The structure of JNJ-64619178, Figure 1, also contains a Q2A moiety, and a cocrystal structure of it in complex with PRMT5:MEP50 was recently reported.^21^ Interestingly, both Q2A motifs of JNJ-64619178 and **AM-9747** form a crucial salt-bridge interaction with Glu444. However, they are rotated 180° relative to each other and only partly overlapping in the two cocrystal structures. Whereas **AM-9747** extends into the substrate pocket, JNJ-64619178 extends into the SAM/MTA binding pocket.

### Effect of **AM-9747** on SDMA Levels in HCT116 Cells

**Figure 7:**
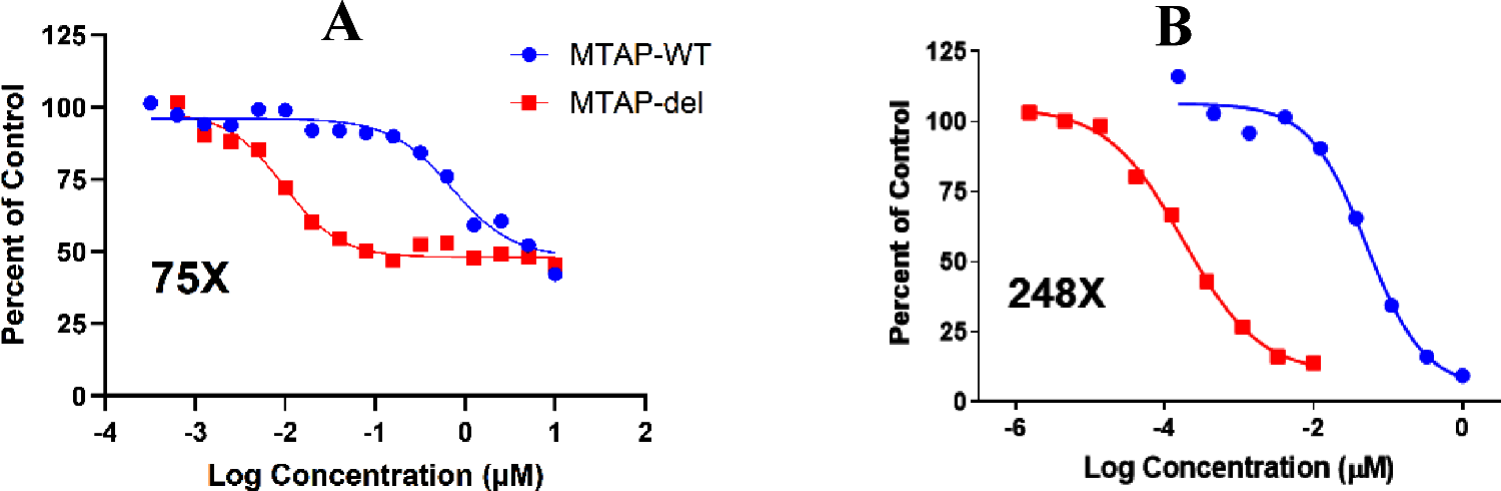
(A) **AM-9747** viability in HCT116 cellular assay (blue circles, MTAP-WT; red squares MTAP-del). Viability was measured by a CellTiter-Glo assay, and cellular MTA-selectivity was determined as (HCT116-WT IC_50_/MTAP-del IC_50_). (B) HCT116-WT and MTAP-del global SDMA levels were assessed by an in-cell imaging assay after 3 days of treatment with **AM-9747**.

Inspired by the cellular potency, selectivity, and relative metabolic stability of **AM-9747**, the symmetrical dimethyl arginine (SDMA) levels were measured in HCT116 MTAP-del and -WT cells. Following treatment with **AM-9747**, the global SDMA levels displayed a concentration-dependent reduction in both cell lines, indicating PRMT5 inhibition of protein arginine dimethylation. As anticipated, this effect was more pronounced in MTAP-del cells (IC_50_ = 0.2 nM) compared to the MTAP-WT cells (IC_50_ = 50 nM). Selectivity defined by SDMA levels (MTAP-WT IC_50_/MTAP-del IC_50_) was even more pronounced, 248-fold, compared to the 75-fold selectivity obtained in the cellular selectivity from the viability assays (Figure 7).

Data in the figure varies slightly from our previously published results^43^ due to minor differences in the assay setup between studies.

### **AM-9747** PK Profile in Mouse

After intravenous (IV) dosing of 1 mg/kg **AM-9747** to mice, clearance was 2.4 mL/min/kg, volume of distribution (Vd *t* = 0) and volume of distribution at steady state (Vd_SS_) were 1.0 and 2.3 L/kg, respectively, and half-life was 2.3 h. Oral dosing (PO) of 5 mg/kg **AM-9747** in mice (CD-1, *n* = 3) resulted in a *C*_max_ = 0.45 μM, AUCinf 1.0 μM h and 23% oral bioavailability (outlined in Table 4).

**Table 4:** PK Characterization of **AM-9747**

Cl_tot_ (mL/min/kg)	Vd_ss_ (L/kg)	*t* (1/2) (h)	*F* (%)^a^	*C*_max_^a^ (μM)	AUC_inf_^a^ (μM h)
2.4	2.3	2.3	23	0.45	1.0

a IV/PO dosing in CD-1 mice (IV (1.0 mg/kg, vehicle: 100% DMSO); PO (5 mg/kg, vehicle: 1% Tween 80, 2% HPMC, 97% water/methanesulfonic acid, pH 2.0)). Values represent an average of *n* = 3.

### **AM-9747** Displays In Vivo MTA Cooperativity in a Pharmacodynamic (PD) Bilateral Tumor Model

**Figure 8:**
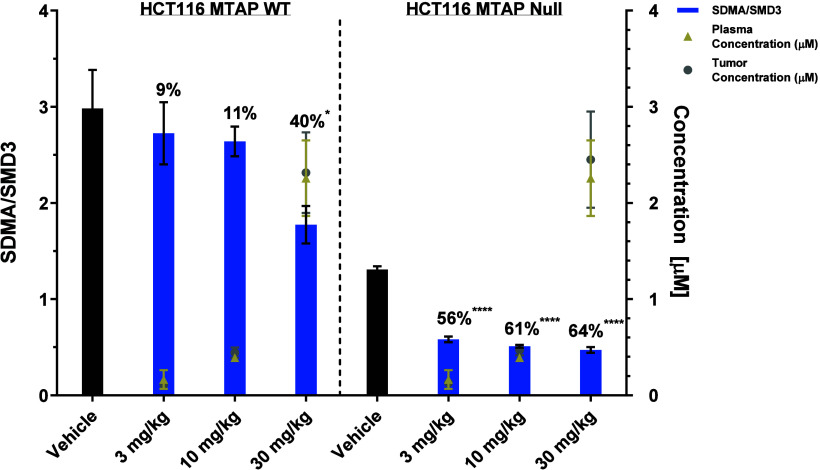
AMG 193 inhibits the growth of MTAP-deleted tumors in vivo. (A) SDMA ELISA analysis of HCT116 MTAP WT and MTAP-deleted bilateral tumors. Mice were administered a total of 4 doses, and tumors were collected 4 h after the last dose. Percentage of inhibition reported relative to the matched vehicle. **AM-9747** was quantified by using LC-SRM MS methods for both plasma and tumor homogenate samples. Data represent mean ± SEM, *n* = 5 for each group. Statistical analysis by one-way ANOVA with Dunnett comparison to control; *P = 0.05, ****P < 0.0001.

**AM-9747** was first evaluated in a four-day SDMA pharmacodynamic (PD) and PK study in mice implanted with HCT116 MTAP-WT cells on one flank and MTAP-del cells on the opposite flank*.* Mice were treated with vehicle or **AM-9747** (3, 10, or 30 mg/kg) PO every day (QD) for 4 days (Figure 8).

Tumors and blood were collected at 4h after the final dose, and the PRMT5-mediated SDMA levels were assessed employing enzyme-linked immunosorbent assay (ELISA), applying SDM3 as a control to determine the reduction of SDMA levels (SDMA/SDM3). **AM-9747** treatment inhibited SDMA signal at all doses evaluated in the MTAP-deleted tumors, however only at the maximum dose of 30 mg/kg was 40% SDMA inhibition observed in the MTAP-WT tumors, compared to a reduction of 64% in the already lower MTAP-del (Figure 8). Further, tumor and plasma mean concentrations were equivalent, indicating that permeability into the tumors was not an issue (Figure 8).

In the MTAP-del tumors, suppression was similar between the 10 and 30 mg/kg doses, despite an increased exposure at the higher dose (Figure 8). This could indicate that a maximum level of suppression may have been reached. However, it has been suggested that the remaining SDMA signal might be due to the limited duration of dosing.^21^ To further investigate this, we extended the dosing period to 21 days. This extension resulted in an increase in SDMA suppression up to 86–89% at 30 mg/kg; see Supporting Information. The remaining SDMA signal might be attributed to the potential presence of MTAP-expressing stromal cells in the samples.^44^

### **AM-9747** Displays Antitumor Activity in HCT116 MTAP-del Xenografts, but Not in HCT116-WT Xenografts

**Figure 9:**
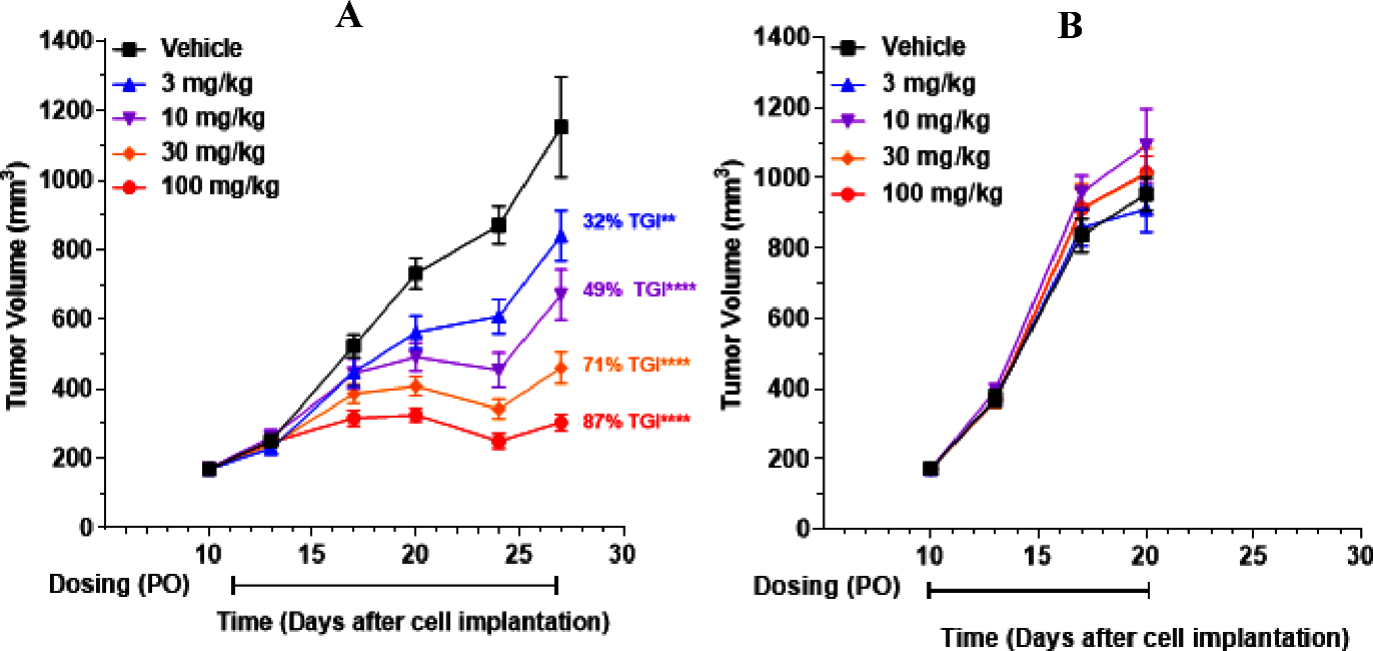
Mouse xenograft efficacy model employing female Athymic nude mice implanted with either HCT116 (A) MTAP-del or (B) MTAP-WT tumors. Vehicle and **AM-9747** were administered a PO QD for the duration of the study. Percentage of inhibition is reported relative to vehicle. Data represents group means ± SEM, *n* = 10. STATS: *P* values were determined by Linear Mixed-Effects Model with Dunnett’s comparison to control. (A) **p < 0.01, ****p < 0.0001. (B) *p* = NS.

Following the PD study, **AM-9747** was investigated for antitumor efficacy in HCT116 xenografts, as shown in Figure 9. To assess selectivity female athymic nude mice implanted with either HCT116 MTAP-del or -WT tumors were treated PO QD with vehicle or **AM-9747** (3, 10, 30, and 100 mg/kg) for the duration of the study. Tumor volume was measured twice a week throughout the study. Tumor growth inhibition (TGI) was observed in a dose-dependent manner, with inhibition of 32, 49, 71, and 87% at 3, 10, 30, and 100 mg/kg, respectively, in the MTAP-del group. In contrast, no effects upon tumor size were observed in the HCT116 MTAP-WT group, as shown in Figure 9. Furthermore, **AM-9747** was well tolerated, displaying no significant effects on body weight in the HCT116 xenograft PD or efficacy studies.

### **AM-9747** Exhibits Significant Antitumor Efficacy in Endogenous MTAP-del Cell-Derived Xenografts

**Figure 10:**
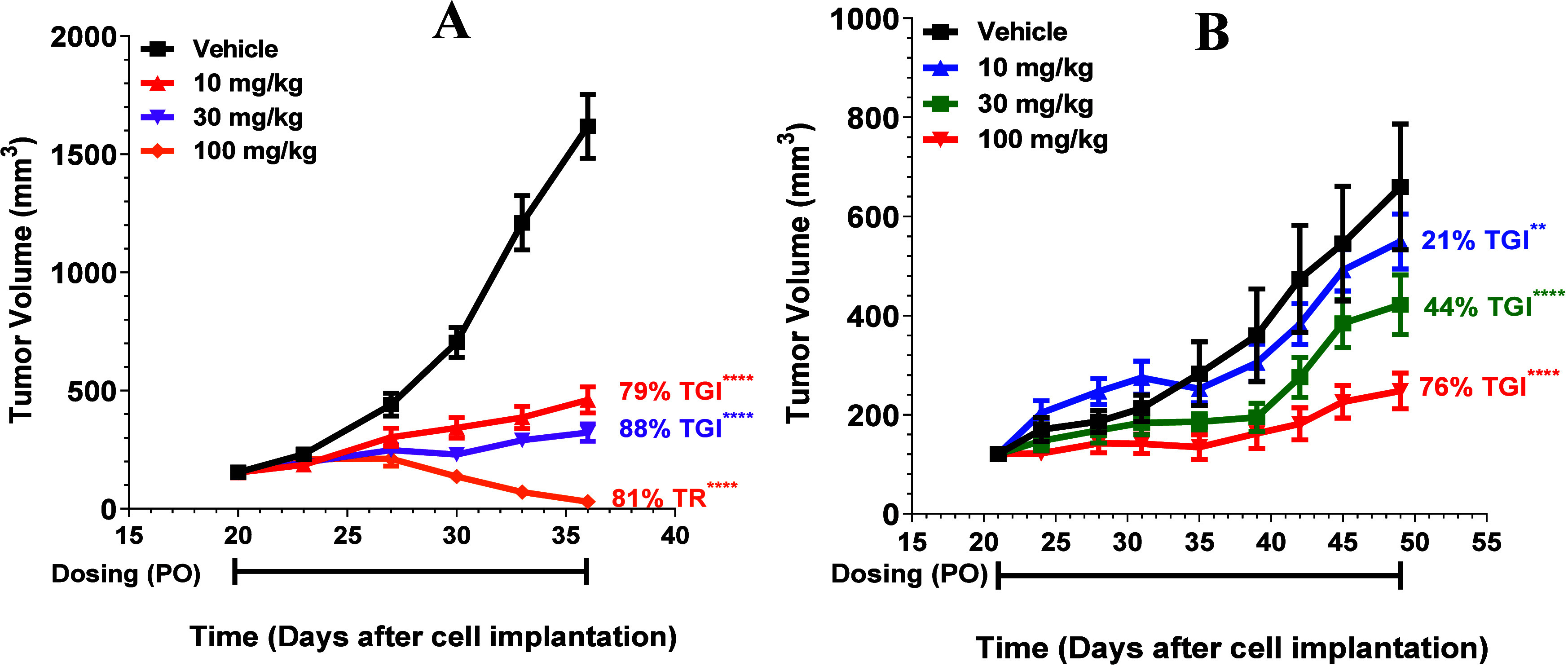
(A) CB17 SCID mice were implanted with DOHH-2 or (B) BxPC-3 tumors. Vehicle and **AM-9747** were administered PO QD for the duration of the study. Data represent group means ± SEM, *n* = 10. STATS: *P* values were determined by linear mixed-effects model with a Dunnett’s comparison to control; **p < 0.01, ****p < 0.0001.

Thereafter, **AM-9747** was examined for its ability to inhibit growth in two endogenous MTAP-del cell-line derived xenografts (CDX) derived from DOHH-2 (Diffuse Large B-cell lymphoma) or BxPC-3 (pancreatic adenocarcinoma) in female CB17 SCID mice, Figure 10. Mice bearing CDX tumors were administered with vehicle or **AM-9747** (10, 30, or 100 mg/kg) PO QD, as shown in Figure 10. The group with DOHH-2 tumors displayed dose-dependent TGI, demonstrating 81% tumor regression (TR) with the 100 mg/kg dose. Likewise, the BxPC-3 group also resulted in a dose-dependent response, resulting in 76% TGI with a 100 mg/kg dose.

### **AM-9747** Exhibits Significant Antitumor Efficacy in Pancreatic and Esophageal PDX Efficacy Models

**Figure 11:**
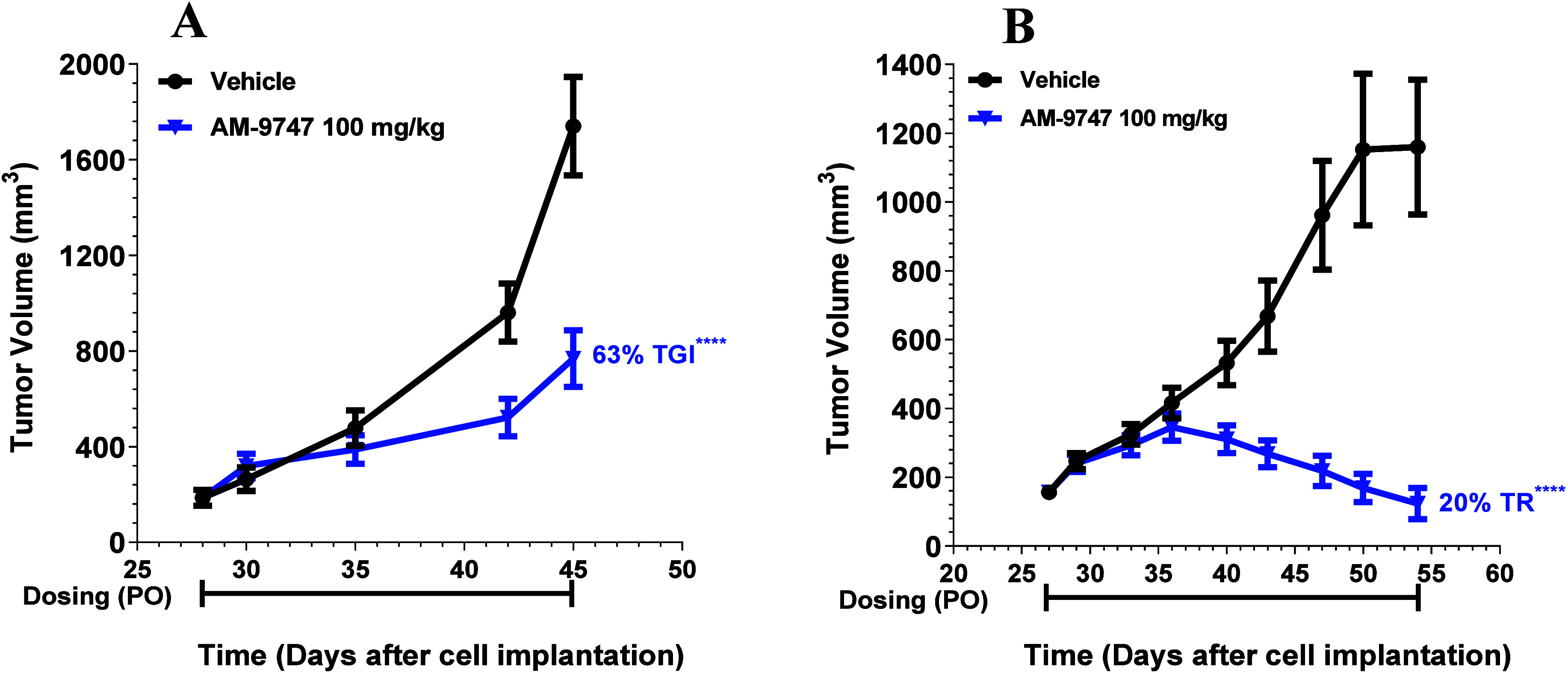
(A) On day 28, female NOD/SCID mice bearing PA5415 (pancreatic tumors) were sorted into two groups, and dosing was initiated. Vehicle and **AM-9747** were administered PO QD for 18 days. Plotted data represent group means ± SEM, *n* = 10 for each group. (B) On day 27, female NOD/SCID mice bearing ES11082 (esophageal tumors) were sorted into two groups, and dosing was initiated. Vehicle and **AM-9747** were administered PO QD for 28 days. Plotted data represent group means ± SEM, *n* = 10 for each group. STATS: *P* values were determined by linear mixed-effects model with a Dunnett’s comparison to control; ****p < 0.0001.

Finally, **AM-9747** was evaluated for efficacy in inhibiting tumor growth in endogenous MTAP-del patient-derived xenografts (PDX), as shown in Figure 11. PDX models have tumor tissues from patients implanted into immunocompromised or humanized mice. These tumors are believed to conserve the original tumor biology such as heterogeneous histology, clinical biomolecular signature, malignant phenotypes and genotypes, tumor architecture, and tumor vasculature. Therefore, they offer a translational research model for evaluating efficacy.^45^

Female NOD/SCID mice bearing pancreatic tumors (PA5415) or esophageal tumors (ES11082) were treated with vehicle or **AM-9747** 100 mg/kg PO QD producing robust antitumor activity against both tumors. The mice bearing pancreatic tumors displayed a TGI of 63%, whereas a TR of 20% was observed for the mice bearing esophageal tumors. Combining these data demonstrates that MTA-cooperative PRMT5 inhibitor **AM-9747** inhibits MTAP-deleted tumor growth in vivo across a variety of tumor lineages and models.

### **AM-9747** Is Highly Selective with Minimal Off-Target Liabilities

To gain further confidence in the series, **AM-9747** underwent pharmacological off-target profiling at Eurofins. It was tested in 83 assays at Cerep and for 100 kinases employing DiscoverX KINOME*scan*. The only notable activity was as an agonist against the μ-opioid receptor (EC50 = 3 μM), see the Supporting Information. Additionally, it was examined in the IonChannelProfiler Qpatch and FLIPR assays without any significant effects. Satisfyingly, this pointed toward **AM-9747** and related compounds being selective and exhibiting minimal off-target effects.

### Chemistry

**Scheme 2:**
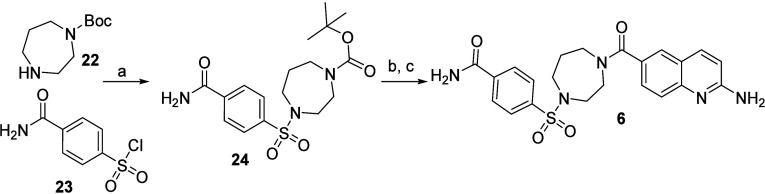
Synthesis of **6** Reaction conditions: (a) DIPEA, DCM, RT; (b) TFA, DCM, RT; (c) 2-aminoquinoline-6-carboxylic acid, EDC, HOAt, DIPEA, DMF, RT.

**Scheme 3:**
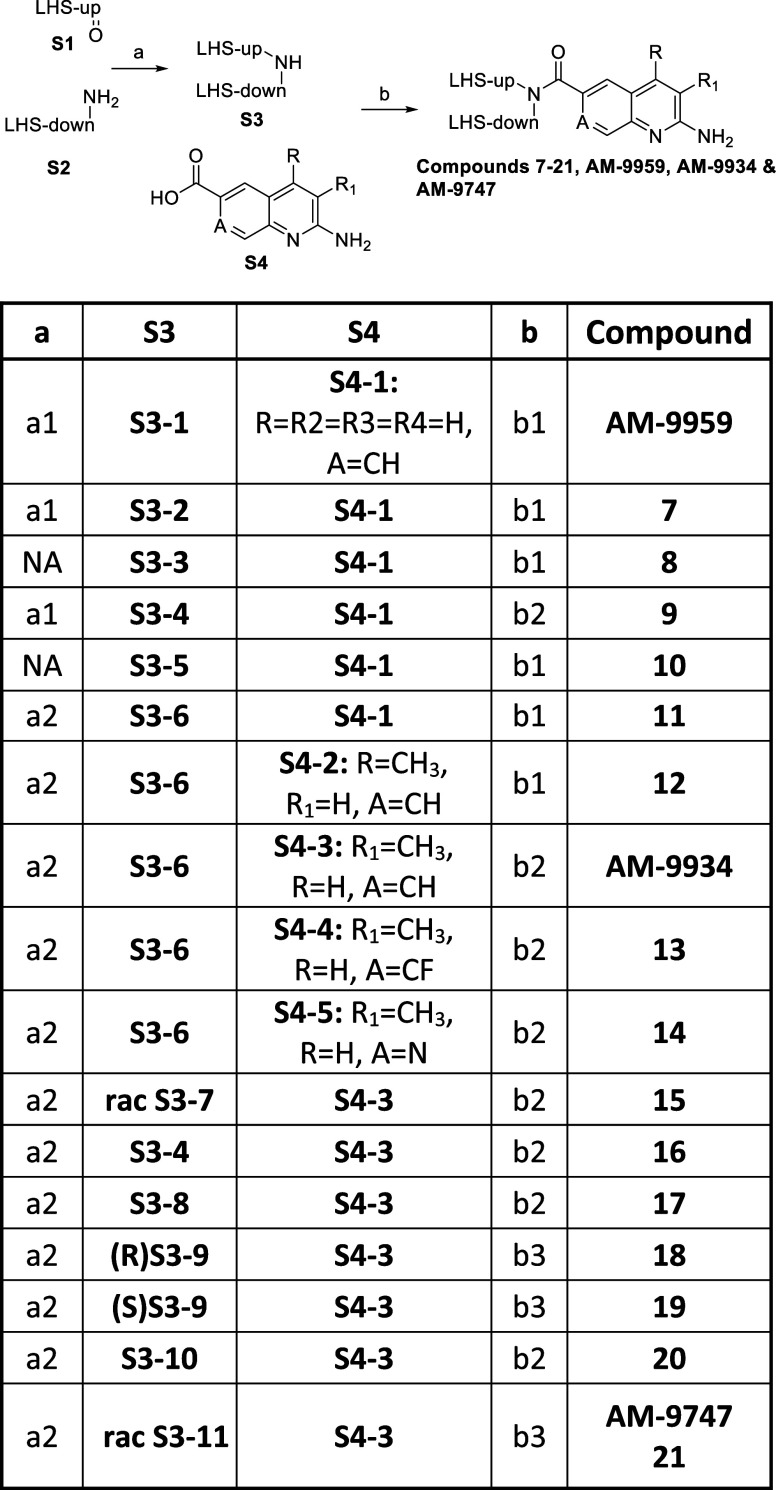
General Synthesis of the Compounds Reagents and conditions: (a) reductive amination (a1) NaCNBH_3_, MeOH; (a2) Na(OAc)_3_BH, DCM, HOAc; (b); amide coupling (b1) EDC·HCl, HOAt, DIPEA, DMF; (b2) HATU, DMF, TEA, or DIPEA; (b3) PyBroP, DMAc or DMF, TEA, or DIPEA. NA= not applicable.

The initial solid- and solution-phase syntheses of the preliminary ligand **5** are outlined in the Supporting Information. The synthesis of **6,** the first validated inhibitor, is outlined in Scheme 2. Thereafter, all the compounds were synthesized via a versatile general synthesis, a reductive amination followed by an amide coupling, as outlined in Scheme 3. This approach enabled a combinatorial exploration of the LHS-up, LHS-down, and RHS SAR.

The reductive aminations were achieved either through imine formation and reduction of the imine with NaBH_4_ or by reductive amination with either NaCNBH_3_ or Na(OAc)_3_BH. These 2°-amines (**S3**) were thereafter used in an amide coupling employing either HATU, EDC/HOAt, or PyBroP and the corresponding carboxylic acids (**S4**).

**Scheme 4:**
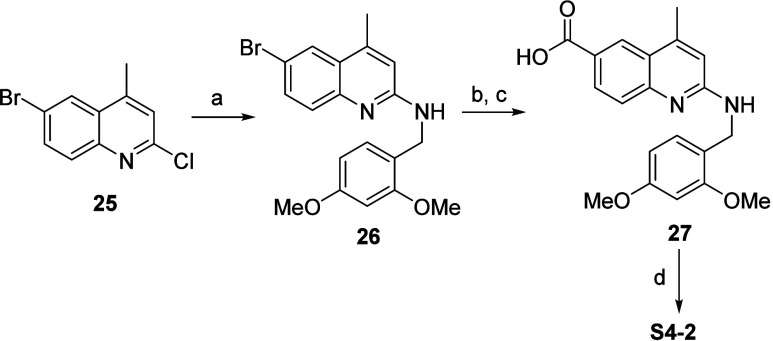
Synthesis of 4-Me-Q2A Acid Reaction conditions: (a) (2,4-dimethoxyphenyl)methanamine, DIPEA, DMSO, 80–100 °C; (b) CO, MeOH, DIPEA, Pd(OAc)_2_, xantphos, DMF, 80 °C; (c) LiOH, THF, MeOH, H_2_O; (d) TFA, DMSO, 50 °C.

The corresponding 4-Me-Q2A acid was synthesized by a S_N_Ar reaction between **25** and (2,4-dimethoxyphenyl)methanamine to yield **26**, followed by Pd-catalyzed carbonylation^46^ and methyl ester deprotection producing **27**, and final deprotection (to remove the DMPM protective group) yielded **S4-2**, outlined in Scheme 4.

**Scheme 5:**
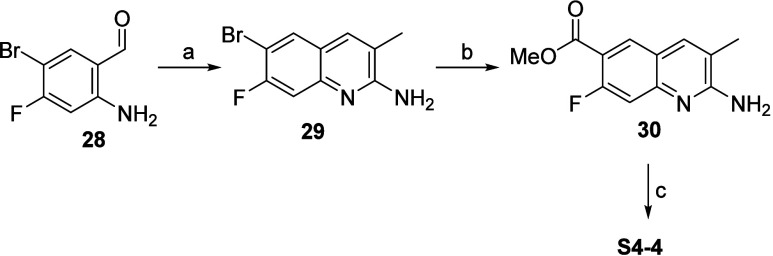
Synthesis of Q2A Acid **S4-4** via the tBuOK-Mediated Friedländer Q2A Synthesis Reaction conditions: (a) propionitrile, ^t^BuOK, DMSO, 50 °C; (b) CO, MeOH, DIPEA, Pd(OAc)_2_, xantphos, DMF, 80 °C; or dppf, Pd(OAc)_2_, CO, MeOH, DMSO, 80 °C; (c) LiOH hydrolysis, RT.

**Scheme 6:**
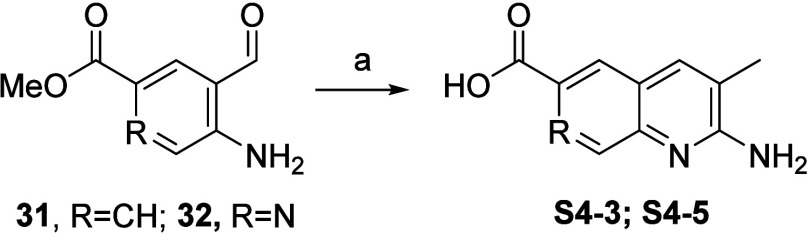
Synthesis of Q2A Acids **S4-3** and **S4-5** via the tBuOK-Mediated Friedländer Q2A Synthesis Reaction conditions: (a) propionitrile, ^t^BuOK, DMSO, 50 °C.

For the 3-Me-Q2A acids, we developed the tBuOK mediated, transition-metal free, Friedländer-type quinoline synthesis. Utilizing propionitrile **S4-5** (Scheme 5), **S4-3** and **S4-5** (Scheme 6) were synthesized.

Thereafter, these acids were subjected to an amide coupling, giving the final compounds **13**, **14,** and **AM-9747**; Scheme 3. Since our initial disclosure of the tBuOK mediated Friedländer Q2A synthesis,^47^ the usefulness and wide applicability of this reaction have since been further explored.^48^ The enantiomers **18** and **19** were synthesized from the corresponding optically pure amines (**S3-9**), whereas compounds **15** and **21** and **AM-9747** were isolated from racemates by SFC.

## Conclusions

We have shown that targeting a specific vulnerability present in MTAP-deleted tumors results in an effective and highly selective novel cancer therapy. Ligands that bound PRMT5 in the presence of MTA were identified by screening a DEL library against the preformed PRMT5-MTA complex. Directly from the screening, one hit identified after structure elucidation, **AM-9959**, displayed an IC_50_ of 130 nM in the MTase-Glo assay (MTA+, 5 μM MTA added) and a nine-fold selectivity compared to the corresponding biochemical assay without added MTA (MTA-).

Initial optimization efforts successfully ameliorated P-gp and BCRP mediated efflux by replacing a 1° amide with EWG groups such as −CF_3_ or −Br. Combining these modifications with substituting the phenyl to a pyridine decreased *c*log *P* for the halogenated phenylene compounds, see Supporting Information. Insights into the ligand's cooperative binding to the PRMT5-MTA complex were unearthed by computer-aided modeling. These insights directed further synthesis of Q2A analogues, leading to the identification of **AM-9934**. **AM-9934** displayed remarkable biochemical MTA+ activity, surpassing the theoretical assay limit. Further, it displayed an IC_50_ below 300 nM in the viability assay of the MTAP-del cell line combined with a 15-fold selectivity.

Additional design and synthesis cycles improved cellular potency and enhanced selectivity, leading to **AM-9747** that displayed an impressive IC_50_ of 9.5 nM in the MTAP-del viability assay and an outstanding 75-fold selectivity over the corresponding isogenic MTAP-WT cellular viability. Moreover, **AM-9747** effectively inhibited arginine dimethylation in HCT116 MTAP-del with an IC_50_ of 2.7 nM and an exquisite 248-fold selectivity compared to the HCT116 MTAP-WT cells (520 nM).

X-ray cocrystal structures of the ternary **AM-9747**+**PRMT5:MEP50**+**MTA** complex confirmed the binding mode of Q2A from the modeling, showing a tight fit and strong hydrogen bonding to Glu444 between the methyl-substituted Q2A RHS and the inner part of the ligand binding pocket as well as a hydrophobic contact to the sulfur of MTA supporting the MTA cooperativity. The binding is further substantiated by additional hydrogen bonds and several hydrophobic interactions between the LHS and the outer part of the ligand binding pocket.

Even despite its modest bioavailability in mice, 23%, a once daily oral administration of **AM-9747** through 17 days of dosing resulted in tumor growth inhibition and even tumor regression (up to 81%) in a dose-dependent manner in mouse CDX models. Moreover, **AM-9747** showed a significant oral antitumor effect (up to 20% TR) in mouse models employing PDXs at 100 mg/kg.

These findings underscore the tremendous potential of targeting the PRMT5-MTA complex for selective inhibition in the treatment of cancers with the homozygous deletion of the *MTAP* gene. These proof-of-concept results directly fueled our PRMT5 program with confidence, ultimately leading to the discovery of **AMG 193**.^28^

## Experimental Section

Unless otherwise noted, reagents and solvents were purchased from commercial suppliers and used without purification, reactions were performed at ambient temperature in anhydrous solvents, and reactions employing air- or moisture-sensitive reagents were performed under a N_2_ or Ar atmosphere. Nuclear magnetic resonance spectra (^1^H and ^13^C) were performed on Bruker NMR instruments at the frequency and temperature indicated. Chemical shifts (δ) are reported in parts per million (ppm) relative to either tetramethylsilane (TMS) or the known chemical shift of the residual solvent, which was used as the spectral reference. Coupling constants (*J*) given are reported in Hz.

Flash chromatography (Flash CC) was performed on an ISCO CombiFlash, Teledyne CombiFlash, or Biotage Isolera instrument using the prepacked columns. Preparative HPLC was performed on an Agilent Prep 1290 instrument with UV and MS(SQ). Analytical Column: PoroShell 120 SB C18 Column Agilent (4.6 × 150 mm, 4 μM) at a flow rate of 1.5 mL/min. Preparative Column: Agilent PoroShell 120 SB C18 Column (21.2 × 150 mm, 4 μM) at a flow rate of 30 mL/min using the solvents indicated, such as *Solvent A: 0.1% formic acid in aqueous and Solvent B: 0.1% formic acid in MeCN. Gradient, 95% A to 100% B over 10–30 min*. MS Settings: Positive mode, UV from 190 to 400 nm, MS spectrum from 150 to 1300 *m*/*z*.

UV chromatography and low-resolution MS data were obtained using either an Agilent G1956B MSD SL coupled with a 6120B, 6130B, or 6140A quadrupole or alternatively a Waters Acquity UPLC-PDA system coupled to a SQD1Mass Spectrometer, all applying electrospray ionization (ESI).

Analytical data (purity at 254 nM, HRMS, and concentration) were established employing an Agilent 1290 Infinity II LC System coupled with a TOF mass spectrometer Agilent 6230B, equipped with a diode array detector and an Antek 8060 nitrogen-specific chemiluminescent detector. Column: Acquity Premier UPLC BEH C18 column (1.7 μm, 2.1 × 50 mm, Waters) at 45 °C. Solvent A: 0.1% formic acid in aqueous and Solvent B: 0.1% formic acid in MeOH. Gradient, 95% A to 100% B over 5.35 min, flow Rate: 0.75 mL/min. MS Settings: Positive mode, mass range from 100 to 3000 *m*/*z*, and UV detection from 190 to 500 nm.

All UPLC-UV of all final compounds, along with NMR spectra of **AM-9934** and **AM-9747,** are provided in the Supporting Information. The purity of all final compounds tested was ≥95% at 254 nM, with the exception of **5**-solid.

### General Methods

#### Reductive Amination 1 (**A1**)

A solution of the aldehyde (1.0–1.2 equiv) and the 1°-amine (1.0–1.2 equiv) in MeOH was stirred for 2 h at RT. The solution was then cooled to 0 °C, NaCNBH_3_ (1.3 equiv) was carefully added in portions, and the reaction was thereafter stirred at RT for 16 h. Ice water was added to quench the reaction and the mixture was concentrated under reduced pressure. The crude 2° amine was either used directly or was purified by Flash CC.

#### Reductive Amination 2 (**A2**)

Glacial acetic acid (1.0–1.1 equiv) was added to a mixture of aldehyde (1.0–1.1 equiv) and 1°-amine (1.2–1.3 equiv) in DCM. The mixture was stirred at RT for 30 min and then treated with Na(OAc)_3_BH (1.5 equiv). CAUTION EXOTHERMIC REACTION! The reaction was stirred at RT for 1 h and then quenched with satd aq Na_2_CO_3_. The layers were separated, and the aqueous layer was extracted with DCM. The combined organic phase was dried over Na_2_SO_4_ and concentrated in vacuo. The crude 2°-amine was used directly and purified by Flash CC, as indicated.

#### Amide Formation 1 (**B1**)

Neat DIPEA (3–4 equiv) was added to a solution of the 2°-amine (1.0–1.2 equiv), the Q2A carboxylic acid (1.0–1.2 equiv), HOAt (1.4 equiv), and EDC (1.4 equiv) in dry DMF and the reaction was stirred from 2 to 16 h at RT-45 °C. The reaction mixture was diluted with EtOAc and washed (×2) with aq LiCl (5%), satd aq NaHCO_3_ (×2), dried over Na_2_SO_4_, filtered, and concentrated under reduced pressure. The residue was purified, as indicated, to yield the final compound.

#### Amide Formation 2 (**B2**)

HATU (1.25 equiv) was added to a mixture of 2-amino-3-methylquinoline-6-carboxylic acid (1.0–1.1 equiv), 2° amine (1.0–1.1 equiv), and TEA or DIPEA (3–5 equiv) in dry DMF at RT. The reaction was stirred at RT-45 °C for 2–16 h whereafter H_2_O and EtOAc were added. The layers were separated, and the organic layer was washed with NaOH (1 M) or aq sat NaHCO_3_, dried over Na_2_SO_4_, filtered, and concentrated under reduced pressure. The residue was purified as indicated to yield the final compound.

#### Amide Formation 3 (**B3**)

PyBroP (1.15 equiv) was added to a mixture of 2° amine (1 equiv), the Q2A carboxylic acid (1.15 equiv), and DIPEA (3–5 equiv) in DMAc or DMF. The reaction was stirred at RT 45 °C for 2–16 h. The reaction was quenched with H_2_O and EtOAc was added, and the layers were separated. The organic layer was washed with NaOH (1M) or NaHCO_3_ and concentrated. The residue was purified, as indicated, to yield the final compound. Alternatively, the reaction mixture was used directly or applied onto SiO_2_, and purified as indicated.

#### 4-((4-(2-Aminoquinoline-6-carbonyl)-1,4-diazepan-1-yl)sulfonyl)benzamide (**6**)

##### Tert-Butyl 4-((4-carbamoylphenyl)sulfonyl)-1,4-diazepane-1-carboxylate (**24**)

DIPEA (1.2 mL, 6.8 mmol) was added to a solution of *tert*-butyl 1,4-diazepane-1-carboxylate (550 mg, 2.7 mmol) and 4-carbamoylbenzenesulfonyl chloride (500 mg, 2.3 mmol) in 1,4-dioxane (6 mL). The reaction mixture was stirred for 2 h and then concentrated under reduced pressure. The residue was dissolved in EtOAc (70 mL) and washed with 10% aq KHSO_4_ (2 × 15 mL), sat aq NaHCO_3_ (2 × 15 mL), and brine (15 mL). The organic solution was dried over Na_2_SO_4_, filtered, and concentrated under reduced pressure to yield crude **25** (215 mg, 0.56 mmol, 25% yield), which was used without further purification. ^1^H NMR (CDCl_3_, 400 MHz) δ 7.92–7.98 (m, 2H), 7.84–7.91 (m, 2H), 5.99–6.36 (m, 1H), 5.62–5.96 (m, 1H), 3.46–3.59 (m, 4H), 3.30–3.38 (m, 2H), 3.23–3.29 (m, 2H), 1.90–1.99 (m, 2H), 1.44 (s, 9H). *m*/*z* (ESI): (M + H)^+^ 384.0.

TFA (0.750 mL, 9.7 mmol) was added to a solution of **24** (100 mg, 0.26 mmol) in DCM (1 mL) and the reaction was stirred at RT for 20 min. The solution was concentrated under reduced pressure. Thereafter, the residue, **S4-1** (64 mg, 0.29 mmol), HATU (110 mg, 0.29 mmol), DMF (2 mL), and finally DIPEA (180 μL, 1.0 mmol) were added, and the reaction was stirred at RT for 88 h. The reaction was diluted with EtOAc (75 mL) and MeOH (2 mL). The resulting solution was washed with aq LiCl (5%, 2 × 10 mL) and brine (10 mL), dried over Na_2_SO_4_, filtered, and concentrated under reduced pressure. The remaining material was purified by Flash CC (Amino D, eluting with 0–100% EtOAc in heptane, followed by 0–20% MeOH in EtOAc) to yield **6** (28 mg, 62 μmol, 24% yield). ^1^H NMR (DMSO-*d*_6_, 400 MHz,) δ 7.98–8.16 (m, 3H), 7.82–7.94 (m, 3H), 7.61 (s, 1H), 7.31–7.53 (m, 3H), 6.80 (d, 1H, J = 8.9 Hz), 6.45 (s, 2H), 3.46–3.75 (m, 4H), 3.32–3.45 (m, 4H), 1.78 (br s, 2H). ESI-HRMS: calcd for C_22_H_23_NO_4_S: *m*/*z* [M + H]^+^ = 454.1544; found [M + H]^+^ = 454.1545.

#### 2-Amino-*N*-(4-carbamoylbenzyl)-*N*-(2-fluorobenzyl)quinoline-6-carboxamide (**AM-9959**)

##### 4-(((2-Fluorobenzyl)amino)methyl)benzamide (**S3-1**)

Reductive amination according to **A1** between 4-formylbenzamide and (2-fluorophenyl)methanamine was purified by Flash CC (C18, eluting with MeCN 5–95% in H_2_O, with 0.1% formic acid as an additive) to give **S3-1** (120 mg, 0.46 mmol, 14% yield). *m*/*z* (ESI): 259.1 (M + H)^+^. Amide formation according to **B2** between **S3-1** and **S4-1** was purified by Flash CC (Amino D, eluting with 0–100% EtOAc in heptane, followed by 0–20% MeOH in EtOAc) to yield **AM-9959** (38 mg, 89 μmol, 46% yield).^1^H NMR (DMSO-*d*_6_, 400 MHz, 330 K) δ 7.94 (m, 1H), 7.84 (td, 2H, *J* = 2.0, 8.2 Hz), 7.77 (d, 1H, *J* = 1.9 Hz), 7.5–7.6 (m, 1H), 7.45 (td, 1H, *J* = 0.6, 8.6 Hz), 7.3–7.4 (m, 4H), 7.1–7.2 (m, 2H), 6.80 (d, 1H, *J* = 8.9 Hz), 6.48 (s, 2H), 4.65 (s, 2H), 4.63 (s, 2H). ESI-HRMS: calcd for C_25_H_22_FN_4_O_2_: *m/z.* [M + H]^+^ = 429.1721; found [M + H]^+^ = 429.1722.

#### 2-Amino-*N*-(2-fluorobenzyl)-*N*-(4-(trifluoromethyl)benzyl)quinoline-6-carboxamide (**7**)

##### *N*-(2-Fluorobenzyl)-1-(4-(trifluoromethyl)phenyl)methanamine (**S3-2**)

A mixture of 4-(trifluoromethyl)benzaldehyde (150 mg, 0.81 mmol), (2-fluorophenyl)methanamine (102 mg, 0.81 mmol), and 3Å MS in MeOH (3 mL) were refluxed for 3h. Thereafter, the mixture was cooled, NaBH_4_ (60 mg, 1.6 mmol) was added, and the reaction was stirred ON at RT. Then, the reaction was diluted with EtOAc (25 mL) the solution was washed with aq NaOH (1 M, 50 mL) and the organic phase was dried (Na_2_SO_4_), filtered, concentrated under reduced pressure, and the crude **S4-1** (220 mg, ca. 80% pure) was used directly in the following step.

Amide formation according to **B1** between crude **S3-2** and **S4-1** was purified by Flash CC (SiO_2_, eluting with 0–10% MeOH in DCM) followed by prep-HPLC (C18, 10–90% MeCN in H_2_O, with 0.1% formic acid as an additive) to yield **7** (75 mg, 0.17 mmol, 21% yield) as the formic acid salt.^1^H NMR (DMSO-*d*_6_, 400 MHz, 330 K) δ 8.14 (s, 1H), 7.89 (d, 1H, *J* = 8.8 Hz), 7.78 (d, 1H, *J* = 1.9 Hz), 7.68 (d, 2H, *J* = 8.1 Hz), 7.5–7.6 (m, 1H), 7.46 (s, 3H), 7.3–7.4 (m, 2H), 7.19 (dt, 1H, *J* = 1.1, 7.5 Hz), 7.12 (dd, 1H, *J* = 8.3, 10.3 Hz), 6.80 (d, 1H, *J* = 8.9 Hz), 6.48 (s, 2H), 4.7–4.7 (m, 2H), 4.7–4.7 (m, 2H). ESI-HRMS: calcd for C_25_H_20_F_4_N_3_O: *m*/*z*. [M + H]^+^ = 454.1537; found [M + H]^+^ = 454.1537.

#### 2-Amino-*N*-(4-bromobenzyl)-*N*-(2-fluorobenzyl)quinoline-6-carboxamide (**8**)

Amide formation according to **B1** between N-(4-bromobenzyl)-1-(2-fluorophenyl)methanamine (**S3-3**) and **S4-1** was purified by prep-HPLC (C18, eluting with 5–95% MeCN in H_2_O, with 0.1% formic acid as an additive) to yield **8** (580 mg, 1.25 mmol, 47% yield). ^1^H NMR (DMSO-*d*_6_, 400 MHz, 350 K) δ 7.89 (d, 1H, *J* = 8.8 Hz), 7.75 (d, 1H, *J* = 1.9 Hz), 7.51 (br d, 3H, *J* = 8.5 Hz), 7.4–7.5 (m, 1H), 7.45 (td, 1H, *J* = 0.6, 8.6 Hz), 7.3–7.4 (m, 2H), 7.2–7.2 (m, 3H), 7.1–7.2 (m, 1H), 6.8–6.8 (m, 1H), 6.37 (br s, 2H), 4.64 (br s, 2H), 4.56 (br s, 2H). ESI-HRMS: calcd for C_24_H_20_BrFN_3_O: *m*/*z*. [M + H]^+^ = 464.0768 found; [M + H]^+^ = 464.0766.

#### 2-Amino-*N*-(2-fluorobenzyl)-*N*-((5-(trifluoromethyl)pyridin-2-yl)methyl)quinoline-6-carboxamide (**9**)

##### *N*-(2-Fluorobenzyl)-1-(5-(trifluoromethyl)pyridin-2-yl)methanamine (**S3-4**)

Reductive amination according to **A1** between 5-(trifluoromethyl)picolinaldehyde with (2-fluorophenyl)methanamine was purified by Flash CC (Amino D, eluting with 0–100% EtOAc in heptane) gave **S3-4** (40 mg, 88 μmol, 42% yield). ^1^H NMR (CDCl_3_, 400 MHz) δ 8.78–8.85 (m, 1H), 7.86–7.92 (m, 1H), 7.49–7.54 (m, 1H), 7.34–7.41 (m, 1H), 7.28–7.30 (m, 1H), 7.22–7.27 (m, 1H), 7.09–7.15 (m, 1H), 7.01–7.08 (m, 1H), 4.00–4.04 (m, 2H), 3.89–3.94 (m, 2H). *m*/*z* (ESI): (M + H)^+^ = 285.0.

Amide formation according to **B2** between **S3-4** and **S4-1** was purified directly by Flash CC (Amino D, eluting with 0–100% EtOAc in heptane, followed by 0–20% MeOH in EtOAc) to yield **9** (40 mg, 18 μmol, 88% yield). ^1^H NMR (DMSO-*d*_6_, 400 MHz, 330 K) δ 8.9–8.9 (m, 1H), 8.12 (dd, 1H, *J* = 2.0, 8.2 Hz), 7.87 (d, 1H, *J* = 8.8 Hz), 7.78 (d, 1H, *J* = 1.9 Hz), 7.54 (dd, 1H, *J* = 2.0, 8.6 Hz), 7.5–7.5 (m, 1H), 7.43 (d, 1H, *J* = 8.6 Hz), 7.4–7.4 (m, 1H), 7.3–7.4 (m, 1H), 7.19 (dt, 1H, *J* = 1.1, 7.5 Hz), 7.1–7.2 (m, 1H), 6.79 (d, 1H, *J* = 8.9 Hz), 6.47 (s, 2H), 4.76 (br s, 2H), 4.76 (br s, 2H). ESI-HRMS: calcd for C_24_H_19_F_4_N_4_O: *m*/*z*: [M + H]^+^ = 455.1490; found [M + H]^+^ = 455.1499.

#### 2-Amino-*N*-isobutyl-*N*-(4-(trifluoromethyl)benzyl)quinoline-6-carboxamide (**10**)

Amide formation according to **B2** was between 2-methyl-N-(4-(trifluoromethyl)benzyl)propan-1-amine (**S3-5**) (54 mg, 0.23 mmol) and **S4-1** (44 mg, 0.23 mmol) to give **10** (10 mg, 25 μmol, 11% yield). ^1^H NMR (CD_3_OD, 300 MHz) δ 8.3–8.5 (m, 1H), 8.0–8.19 (m, 1H), 7.77–7.91 (m, 1H), 7.53–7.77 (m, 6H), 7.26–7.45 (m, 1H), 6.86–7.05 (m, 1H), 4.88–4.95 (m, 1H), 4.69–4.81 (m, 1H), 3.35–3.44 (m, 1H), 3.18–3.30 (m, 1H), 2.08–2.31 (m, 1H), 1.86–2.08 (m, 1H), 0.91–1.19 (m, 3H), 0.60–0.91 (m, 3H). ESI-HRMS: calcd for C_22_H_23_F_3_N_3_O: *m*/*z*: [M + H]^+^ = 402.1788; found [M + H]^+^ = 402.1795.

#### 2-Amino-*N*-isobutyl-*N*-((5-(trifluoromethyl)pyridin-2-yl)methyl)quinoline-6-carboxamide (**11**)

##### 2-Methyl-*N*-((5-(trifluoromethyl)pyridin-2-yl)methyl)propan-1-amine (**S3-6**)

Reductive amination according to **A2** between 5-(trifluoromethyl)picolinaldehyde (3.0 g, 17.3 mmol) and isobutylamine (1.5 g, 20.2 mmol) was purified by Flash CC (SiO_2_, eluting with EtOAc/EtOH (3/1) 0–100% in heptane) to afford **S3-6** (2.81 g, 12.1 mmol, 70% yield) as a brown oil. ^1^H NMR (CDCl_3_, 400 MHz) δ 8.81 (s, 1H), 7.88 (dd, 1H, *J* = 8.1, 2.1 Hz), 7.50 (d, 1H, *J* = 8.1 Hz), 3.98 (s, 2H), 2.46 (d, 2H, *J* = 6.6 Hz), 1.73–1.84 (m, 2H), 0.94 (d, 6H, *J* = 6.6 Hz). *m*/*z* (ESI): 233.0 (M + H)^+^.

Amide formation according to **B1** between **S3-6** (93 mg, 0.40 mmol) and **S4-1** (83 mg, 0.44 mmol) was purified by Flash CC (SiO_2_, eluting with 0–10% MeOH in DCM) to yield **11** (10 mg, 25 μmol, 6% yield).^1^H NMR (DMSO-*d*_6_, 400 MHz, 330 K) δ 8.92 (s, 1H), 8.15 (br d, 1H, *J* = 7.4 Hz), 7.92 (br d, 1H, *J* = 8.7 Hz), 7.70 (s, 1H), 7.71 (d, 1H, *J* = 1.4 Hz), 7.5–7.6 (m, 1H), 7.49 (dd, 1H, *J* = 1.4, 8.6 Hz), 7.45 (d, 1H, *J* = 8.6 Hz), 6.81 (d, 1H, *J* = 8.8 Hz), 6.52 (s, 2H), 4.81 (br s, 2H), 3.27 (d, 2H, *J* = 7.4 Hz), 1.98 (br s, 1H), 0.82 (br s, 6H). ESI-HRMS: calcd for C_21_H_22_F_3_N_4_O: *m*/*z*: [M + H]^+^ = 403.1740; found [M + H]^+^ = 403.1748.

#### 2-Amino-*N*-isobutyl-4-methyl-*N*-((5-(trifluoromethyl)pyridin-2-yl)methyl)quinoline-6-carboxamide (**12**)

##### 6-Bromo-*N*-(2,4-dimethoxybenzyl)-4-methylquinolin-2-amine (**26**)

A solution of 6-bromo-2-chloro-4-methylquinoline (300 mg, 1.17 mmol), (2,4-dimethoxyphenyl)methanamine (215 mg, 1.29 mmol), and DIPEA (450 mg, 0.51 mmol) in DMSO (2 mL) was shaken at 80 °C for 5 h. Thereafter, additional (2,4-dimethoxyphenyl)methanamine (215 mg, 1.29 mmol) was added, and the reaction was shaken at 80 °C for an additional 16 h, followed by 2 h at 100 °C. The reaction was cooled to RT and diluted with EtOAc (75 mL). The organic solution was then washed aq LiCl (5%, 2 × 10 mL), sat aq NaHCO_3_ (2 × 10 mL), and brine (10 mL). Thereafter, the washed solution was dried (Na_2_SO_4_), filtered, and concentrated under reduced pressure. The residue was purified by Flash CC (amino D column, eluting with 0 to 100% EtOAc in heptane) to yield **26**. ^1^H NMR (400 MHz, DMSO-*d*_6_) δ ppm 7.84–7.89 (m, 1H), 7.51–7.57 (m, 1H), 7.38–7.44 (m, 1H), 7.22–7.28 (m, 1H), 7.17–7.21 (m, 1H), 6.70–6.75 (m, 1H), 6.55–6.58 (m, 1H), 6.43–6.48 (m, 1H), 4.43–4.50 (m, 2H), 3.81 (s, 3H), 3.71–3.75 (m, 3H), 2.41–2.47 (m, 3H). *m*/*z* (ESI): (M + H)^+^ 386.9.

##### 2-((2,4-Dimethoxybenzyl)amino)-4-methylquinoline-6-carboxylic Acid (**27**)

The carbonylation was performed in a two-chambered COware vial sealed with septa. Chamber A contained a solution of formic acid (92 mg, 2 mmol) and methanesulfonyl chloride (229 mg, 2 mmol) in toluene (2 mL). Chamber B contained a mixture of **26** (110 mg, 0.28 mmol), methanol (91 mg, 0.120 mL, 2.8 mmol), DIPEA (110 mg, 0.85 mmol), Pd(OAc)2 (6.4 mg, 0.028 mmol), and xantphos (16 mg, 0.028 mmol) in DMF (2 mL). The COware was evacuated and backfilled with Ar (x3). Thereafter, triethylamine (0.56 mL, 4 mmol) was added to chamber A and the vial was stirred at 80 °C for 16 h. After being cooled to RT, the contents of chamber B were transferred to a separatory funnel with EtOAc (50 mL). The organic phase was washed with aq sat NaH_2_PO_4_ (10 mL) and aq LiCl (5%, 2 × 10 mL). The organic solution was dried (Na_2_SO_4_), filtered, and concentrated under reduced pressure. The remaining material contained a mixture of methyl 2-((2,4-dimethoxybenzyl)amino)-4-methylquinoline-6-carboxylate and 2-((2,4-dimethoxybenzyl)amino)-4-methylquinoline-6-carboxylic acid. This mixture was then dissolved in THF (1 mL), MeOH (1 mL), and aq LiOH (2 N, 0.25 mL, 0.49 mmol) and stirred at 60 °C for 16 h. The reaction mixture was then diluted with EtOAc (75 mL), washed with sat aq NaH_2_PO_4_ (2 × 10 mL), and brine (10 mL). The organic phase was dried (Na_2_SO_4_), filtered, and concentrated under reduced pressure. The residue was purified by flash CC (C18 column, eluting with MeCN 5–95% in H_2_O, with 0.1% formic acid as an additive) to yield **27** (17 mg, 0.048 mmol, 6% yield over 2 steps). ^1^H NMR (DMSO-*d*_6_, 400 MHz) δ 12.38 (br s, 1H), 8.37 (d, 1H, *J* = 1.9 Hz), 7.95 (dd, 1H, *J* = 2.0, 8.6 Hz), 7.50 (d, 1H, *J* = 8.7 Hz), 7.2–7.3 (m, 2H), 6.77 (d, 1H, *J* = 0.8 Hz), 6.58 (d, 1H, *J* = 2.4 Hz), 6.47 (dd, 1H, *J* = 2.4, 8.3 Hz), 4.55 (d, 2H, *J* = 5.7 Hz), 3.84 (s, 3H), 3.75 (s, 3H), 2.52 (d, 3H, *J* = 1.0 Hz). *m*/*z* (ESI): 352.9 (M + H)^+^.

##### 2-Amino-4-methylquinoline-6-carboxylic Acid (**S4-2**)

TFA (1 mL, 13 mmol) was added to a solution of **27** (100 mg, 0.28 mmol) in DMSO (1 mL) and the reaction was shaken at 50 °C ON. The resulting semisolid was concentrated under reduced pressure and diluted with EtOAc (25 mL). The solution was treated with aq NaOH (1 M, 10 mL), resulting in a precipitation. The solid material was collected and subsequently washed with ice–water and ice-cold EtOAc. The resulting material was dried under a high vacuum to produce **S4-2** (31 mg, 15 mmol, 53% yield). ^1^H NMR (DMSO-*d*_6_, 400 MHz) δ 8.36 (d, 1H, *J* = 1.67 Hz) 8.01 (dd, 1H, *J* = 8.58, 1.91 Hz) 7.39 (d, 1H, *J* = 8.58 Hz) 6.65 (s, 1H) 6.17 (br s, 2H) 2.53 (d, 3H, *J* = 0.72 Hz).

Amide formation according to **B1** between **S3-6** and **S4-2** was purified by Flash CC (KP-Amino D, eluting with 0–100% EtOAc in heptane) to yield **12** (19% yield). ^1^H NMR (DMSO-*d*_6_, 400 MHz) δ 8.92–8.96 (m, 1 H), 8.16 (dd, 1H, J = 8.23, 2.03 Hz), 7.73 (s, 1H), 7.56 (br s, 1H), 7.39–7.53 (m, 2H), 6.64 (s, 1H), 6.32 (s, 2H), 4.80 (br s, 2H), 3.29 (d, 2H, J = 7.39 Hz), 2.29–2.48 (m, 3H), 2.00 (br d, 1H, J = 9.30 Hz), 0.77–0.82 (m, 1H), 0.84 (br s, 6H). ESI-HRMS: calcd for C_22_H_24_F_3_N_4_O: *m*/*z*: [M + H]^+^ = 417.1897; found [M + H]^+^ = 417.1904.

#### 2-Amino-*N*-isobutyl-3-methyl-*N*-((5-(trifluoromethyl)pyridin-2-yl)methyl)quinoline-6-carboxamide (**AM-9934**)

##### 2-Amino-3-methylquinoline-6-carboxylic Acid (**S4-3**)

Under a N_2_ atmosphere, a solution of ^t^BuOK (1.0 M in THF, 0.84 L, 0.84 mol) was added to a stirred solution of propionitrile (60 mL, 0.84 mol) in DMSO (0.75 L) at 0 °C. After stirring the mixture for 15 min, methyl 4-amino-3-formylbenzoate (75 g, 0.42 mol) was added in portions and then heated at 50 °C for 16 h. The reaction mixture was cooled, diluted with H_2_O (1.0 L), and extracted with EtOAc (2 × 1.0 L). The aqueous layer was adjusted to pH 6.5 with HCl (1.5 M). The resulting solid was filtered, washed with H_2_O (2 × 1.0 L) and acetone (2 × 1.0 L), and dried under vacuum overnight. The solid was stirred in MTBE (1.0 L) for 12 h, filtered, and concentrated under reduced pressure to give **S4-3** (52.3 g, 260 mmol, 62% yield). ^1^H NMR (DMSO-*d*_6_, 400 MHz) δ 10.0 (br s, 1H), 8.24 (d, 1H, *J* = 2.0 Hz), 7.91 (dd, 1H, *J* = 8.7, 2.0 Hz), 7.86 (s, 1H), 7.46 (d, 1H, *J* = 8.7 Hz), 6.67 (s, 2H), 2.22 (s, 3H). *m*/*z* (ESI): 203.0 (M + H)^+^.

Amide formation according to **B2** between **S3-6** and **S4-3** was purified by Flash CC (SiO_2_, eluting with 20–90% EtOAc in heptane) to give **AM-9934** (177 mg, 0.43 mmol, 82% yield) as a light-yellow solid.^1^H NMR (DMSO-*d*_6_, 400 MHz, 340 K) δ 8.9–8.9 (m, 1H), 8.14 (dd, 1H, *J* = 1.8, 8.2 Hz), 7.74 (s, 1H), 7.65 (d, 1H, *J* = 1.3 Hz), 7.5–7.6 (m, 1H), 7.46 (d, 1H, *J* = 8.6 Hz), 7.43 (dd, 1H, *J* = 2.0, 8.5 Hz), 6.23 (s, 2H), 4.81 (s, 2H), 3.28 (d, 2H, *J* = 7.4 Hz), 2.2–2.2 (m, 3H), 1.9–2.0 (m, 1H), 0.82 (br s, 6H). ESI-HRMS: calcd for C_22_H_24_F_3_N_4_O: *m*/*z*: [M + H]^+^ = 417.1897; found [M + H]^+^ = 417.1896.

#### 2-Amino-7-fluoro-*N*-isobutyl-3-methyl-*N*-((5-(trifluoromethyl)pyridin-2-yl)methyl)quinoline-6-carboxamide (**13**)

##### 6-Bromo-7-fluoro-3-methylquinolin-2-amine (**29**)

A solution of tBuOK (1 M in THF, 275 mL, 275 mmol) was added to a round-bottomed flask containing propionitrile (19.7 mL, 275 mmol) and DMSO (300 mL). The mixture was cooled with an ice bath, and then 2-amino-5-bromo-4-fluorobenzaldehyde (30 g, 138 mmol) was added in portions. The reaction was heated at 50 °C for 12 h, cooled to RT, and quenched by the slow addition of water (1 L). The precipitated white solid was filtered, washed with water (0.5 L), and dried under a vacuum. The white solid was triturated with diethyl ether (0.5 L) and filtered to obtain **29** (25 g, 98 mmol, 71%). ^1^H NMR (DMSO-*d*_6_, 400 MHz) δ 7.97 (d, 1H, *J* = 8.0 Hz), 7.73 (s, 1H), 7.29 (d, 1H, *J* = 10.9 Hz), 6.60 (s, 2H), 2.19 (s, 3H). *m*/*z* (ESI): 255.0 (M + H)^+^.

##### Methyl-2-amino-7-fluoro-3-methylquinoline-6-carboxylate (**30**)

A 250 mL autoclave pressure reactor was loaded with **29** (10 g, 39.2 mmol), dppf (6.5 g, 11.8 mmol), TEA (13.6 mL, 98 mmol), and Pd(OAc)_2_ (1.76 g, 7.84 mmol) in MeOH (60 mL), and DMSO (60 mL). The reaction was stirred at 80 °C under a CO atmosphere (100 psi) for 36 h. The reaction was cooled to RT, diluted with H_2_O (100 mL), and extracted with EtOAc (3 × 100 mL). The combined organic layer was concentrated under reduced pressure, and the residue was absorbed onto a plug of SiO_2_ and purified by Flash CC (SiO_2_, eluting with 70–100% EtOAc in hexanes) to provide **30** (6.0 g, 25.6 mmol, 65% yield) as an off-white solid. ^1^H NMR (DMSO-*d*_6_, 400 MHz) δ 8.24 (d, 1H, *J* = 8.4 Hz), 7.87 (s, 1H), 7.17 (dd, 1H, *J* = 13.4, 0.7 Hz), 6.90 (s, 2H), 3.86 (s, 3H), 2.19 (s, 3H). *m*/*z* (ESI): 235.1 (M + H)^+^.

##### 2-Amino-7-fluoro-3-methylquinoline-6-carboxylic Acid (**S4-4**)

LiOH·H_2_O (3.2 g, 77 mmol) was added to a stirred solution of **30** (6.0 g, 25.6 mmol) in THF (60 mL), MeOH (30 mL), and H_2_O (30 mL). After the reaction was stirred for 12 h at RT the mixture was concentrated, and the residue was acidified with HCl (1 M) to pH ≈ 6. The precipitated brown solid was filtered, washed with MeOH (50 mL), and azeotroped twice with toluene (30 mL) to provide **S4-4** (5 g, 19.0 mmol, 89% yield).^1^H NMR (DMSO-*d*_6_, 400 MHz) δ 8.35 (d, 1H, *J* = 8.0 Hz), 8.13 (s, 1H), 7.99 (br s, 2H), 7.42 (d, 1H, *J* = 12.1 Hz), 2.22 (s, 3H). *m*/*z* (ESI): 221.1 (M + H)^+^.

Amide formation according to **B2** between **S3-6** (38 mg, 0.16 mmol) and **S4-4** (36 mg, 0.16 mmol) and subjected to prep-HPLC (C18, 10 μm eluting with 15–95% MeCN in H_2_O, using 0.1% TFA as an additive) to yield the title product as the TFA salt (41 mg, 77 μmol, 48% yield).^1^H NMR (DMSO-*d*_6_, 400 MHz, 375 K) δ 8.69 (br s, 1H), 8.05 (br s, 1H), 7.9–8.0 (m, 1H), 7.83 (br t, 2H, *J* = 7.5 Hz), 7.40 (d, 1H, *J* = 11.0 Hz), 5.0–6.1 (very br s, 2H), 4.7–4.9 (m, 2H), 3.20 (br s, 2H), 2.3–2.3 (m, 3H), 1.9–2.1 (m, 1H), 0.81 (br s, 6H). ESI-HRMS: calcd for C_22_H_23_F_4_N_4_O: *m*/*z*: [M + H]^+^ = 435.1803; found [M + H]^+^ = 435.1811.

#### 2-Amino-*N*-isobutyl-3-methyl-*N*-((5-(trifluoromethyl)pyridin-2-yl)methyl)-1,7-naphthyridine-6-carboxamide (**14**)

##### 2-Amino-3-methyl-1,7-naphthyridine-6-carboxylic Acid (**S4-5**)

The compound was prepared according to the method described for **S4-3**(but employing methyl 5-amino-4-formylpicolinate instead of methyl 4-amino-3-formylbenzoate) (**32**) produced **S4-5**. ^1^H NMR (TFA-d, 400 MHz) δ 9.74 (s, 1H), 9.17 (s, 1H), 8.61 (s, 1H), 2.81 (s, 3H). *m*/*z* (ESI): 204.1 (M+H)^+^. Amide formation according to **B3** between amine and **S4-5** was purified by prep-HPLC (C18, eluting with 5–95% MeCN in H_2_O, with 0.1% formic acid as an additive) to yield **14** (22 mg, 55% yield). ^1^H NMR (400 MHz, DMSO-*d*_6_) δ ppm 8.61–9.04 (m, 2H) 8.02–8.31 (m, 1H) 7.81 (br s, 2 H) 7.42–7.73 (m, 1H) 6.68 (br s, 2H) 4.79–5.13 (m, 2H) 3.27–3.61 (m, 2H) 3.19 (s, 2H) 2.27 (br s, 3H) 2.01–2.17 (m, 1H) 1.78–1.99 (m, 1H) 0.83–1.05 (m, 3H) 0.60–0.83 (m, 3H). ESI-HRMS: calcd for C_21_H_24_F_3_N_5_O: *m*/*z*: [M + H]^+^ = 419.1933; found [M + H]^+^ = 419.1898.

#### 2-Amino-3-methyl-*N*-(3,3,3-trifluoro-2-methylpropyl)-*N*-((5-(trifluoromethyl)pyridin-2-yl)methyl)quinoline-6-carboxamide (**15**)

##### 3,3,3-Trifluoro-2-methyl-*N*-((5-(trifluoromethyl)pyridin-2-yl)methyl)propan-1-amine (**S3-7**)

Reductive amination according to **A2** between 5-(trifluoromethyl)picolinaldehyde and 2-(trifluoromethyl)propylamine was purified by Flash CC (SiO_2_, eluting with 1 to 20% of MeOH containing 0.5% ammonium hydroxide in DCM to yield **S3-7** (780 mg, 2.7 mmol, 73% yield) as a colorless oil. ^1^H NMR (CDCl_3_, 400 MHz) δ 8.8–8.9 (m, 1H), 7.90 (dd, 1H, *J* = 2.1, 8.2 Hz), 7.48 (d, 1H, *J* = 8.2 Hz), 3.9–4.1 (m, 2H), 2.96 (dd, 1H, *J* = 5.4, 12.3 Hz), 2.65 (dd, 1H, *J* = 7.4, 12.2 Hz), 2.3–2.6 (m, 2H), 1.20 (d, 3H, *J* = 7.1 Hz).

Amide formation according to **B2** between **S3-7** and **S4-3** was purified by prep-HPLC (C18 10 μm, eluting with 10–95% MeCN in H_2_O, with 0.1% TFA as an additive) to yield **rac 15** as the TFA salt (101 mg, 0.17 mmol, 35%). The racemate was optically resolved by prep-SFC (Chiralpak AD 250 × 21 mm, 5 μm, eluting with 65% liquid CO_2_ and 35% iPrOH, using 0.2% TEA as an additive) to give **15** as the first eluting peak (56 mg, > 95% ee) and the enantiomorph as the second eluting peak (38 mg, >95% ee).

Thereafter, **15** was further purified by prep HPLC (C18 10 μm column, eluting with 10 to 85% MeCN in H_2_O, with 0.1% TFA as an additive) to yield **15** (35 mg, > 95% ee, 35% yield) as the TFA salt. ^1^H NMR (DMSO-*d*_6_, 400 MHz, 340 K) δ 8.9–9.0 (m, 1H), 8.3–8.6 (m, 2H), 8.2–8.2 (m, 1H), 8.13 (br d, 1H, *J* = 7.6 Hz), 7.8–7.9 (m, 1H), 7.67 (dd, 2H, *J* = 8.3, 12.8 Hz), 7.3–7.6 (m, 1H), 4.7–4.9 (m, 2H), 3.68 (dd, 1H, *J* = 6.4, 14.2 Hz), 3.5–3.6 (m, 1H), 2.9–3.1 (m, 1H), 2.3–2.4 (m, 3H), 1.0–1.3 (m, 3H). ESI-HRMS: calcd for C_22_H_21_F_6_N_4_O: *m*/*z*: [M + H]^+^ = 471.1609; found [M + H]^+^ = 471.1620.

##### (*S*)-2-Amino-3-methyl-*N*-(3,3,3-trifluoro-2-methylpropyl)-*N*-((5-(trifluoromethyl)pyridin-2-yl)methyl)quinoline-6-carboxamide

^1^H NMR (CD_3_OD, 400 MHz) δ: 8.91 (s, 1H), 7.9–8.3 (m, 3H), 7.3–7.9 (m, 3H), 4.9–5.1 (m, 2H), 3.5–4.0 (m, 2H), 2.8–3.1 (m, 1H), 2.38 (br s, 3H), 0.9–1.4 (m, 3H). *m*/*z* (ESI): 471.2 (M + 1)^+^.

#### 2-Amino-*N*-(2-fluorobenzyl)-3-methyl-*N*-((5-(trifluoromethyl)pyridin-2-yl)methyl)quinoline-6-carboxamide (**16**)

Amide formation according to **B2** between **S3-4** and **S4-3** was subjected to Flash CC (KP-Amino D, eluting with 0–100% EtOAc in heptane) to yield **16** (63 mg, 0.13 mmol, 38% yield). ^1^H NMR (DMSO-*d*_6_, 400 MHz, 330 K) δ 8.9–8.9 (m, 1H), 8.12 (dd, 1H, *J* = 2.0, 8.2 Hz), 7.74 (d, 1H, *J* = 1.8 Hz), 7.72 (s, 1H), 7.50 (dd, 2H, *J* = 1.9, 8.6 Hz), 7.45 (d, 1H, *J* = 8.6 Hz), 7.38 (t, 1H, *J* = 7.2 Hz), 7.3–7.4 (m, 1H), 7.19 (dt, 1H, *J* = 1.1, 7.4 Hz), 7.1–7.2 (m, 1H), 6.31 (s, 2H), 4.76 (br s, 2H), 4.75 (s, 2H), 2.22 (d, 3H, *J* = 0.8 Hz). *m*/*z* (ESI): 469.1652 (M+1)^+^. ESI-HRMS: calcd for C_25_H_21_F_4_N_4_O: *m*/*z*: [M + H]^+^ = 469.1646; found [M + H]^+^ = 469.1652.

#### 2-Amino-*N*-((3-fluoropyridin-2-yl)methyl)-3-methyl-*N*-((5-(trifluoromethyl)pyridin-2-yl)methyl)quinoline-6-carboxamide (**17**)

Reductive amination according to **A2** between 5-(trifluoromethyl)picolinaldehyde and (3-fluoro-2-pyridinyl)methanamine was purified by Flash CC (SiO_2_, eluting with 1 to 8% of MeOH containing 0.5% NH_4_OH in DCM) to yield **S3-8** (430 mg, 1.51 mmol, 44% yield) as a colorless oil.^1^H NMR (CDCl_3_, 400 MHz) δ 8.8–8.9 (m, 1H), 8.39 (td, 1H, *J* = 1.4, 4.7 Hz), 7.88 (dd, 1H, *J* = 1.9, 8.2 Hz), 7.56 (d, 1H, *J* = 8.2 Hz), 7.36 (ddd, 1H, *J* = 1.5, 8.3, 9.5 Hz), 7.21 (td, 1H, *J* = 4.3, 8.5 Hz), 4.0–4.1 (m, 4H), 2.5–3.0 (m, 1H). *m*/*z* (ESI): 286.1 (M + 1)^+^.

Amide formation according to **B2** between **S3-8** and **S4-3** was purified by Flash CC Flash CC (SiO_2_, eluting with 1 to 12% of MeOH in DCM) followed by prep-HPLC (C18, eluting with MeCN in H_2_O 5–95%, using TFA as an additive) to yield **17** (49 mg, 0.07 mmol, 48% yield) as the TFA salt. ^1^H NMR (CD_3_OD, 400 MHz) δ 8.7–8.9 (m, 1H), 8.41 (br s, 1H), 8.20 (s, 1H), 8.0–8.1 (m, 2H), 7.92 (br d, 1H, *J* = 7.9 Hz), 7.5–7.7 (m, 2H), 7.3–7.5 (m, 2H), 4.9–5.0 (m, 4H), 2.38 (s, 3H).ESI-HRMS: calcd for C_24_H_20_F_4_N_5_O: *m*/*z*: [M + H]^+^ = 470.1598; found [M + H]^+^ = 470.1609.

#### (*R*)-2-Amino-*N*-(1-(3-fluoropyridin-2-yl)ethyl)-3-methyl-*N*-((5-(trifluoromethyl)pyridin-2-yl)methyl)quinoline-6-carboxamide (**18**)

##### (*R*)-1-(3-Fluoropyridin-2-yl)-*N*-((5-(trifluoromethyl)pyridin-2-yl)methyl)ethan-1-amine (**(R)-S3-9**)

Reductive amination was performed according to **A2** between 5-(trifluoromethyl)picolinaldehyde and (*R*)-1-(3-fluoropyridin-2-yl)ethan-1-amine hydrochloride. The crude reaction mixture was loaded directly onto silica gel and subjected to Flash CC (SiO_2_, eluting with 1 to 16% MeOH with 0.5% ammonium hydroxide in DCM) to yield **(R)-S3-9** (980 mg, 3.3 mmol, 75% yield) as a colorless oil. ^1^H NMR (CDCl_3_, 400 MHz) δ 8.8–8.8 (m, 1H), 8.41 (td, 1H, *J* = 1.3, 4.7 Hz), 7.86 (dd, 1H, *J* = 2.1, 8.2 Hz), 7.53 (d, 1H, *J* = 8.2 Hz), 7.34 (ddd, 1H, *J* = 1.4, 8.3, 9.6 Hz), 7.19 (td, 1H, *J* = 4.3, 8.5 Hz), 4.30 (dq, 1H, *J* = 1.3, 6.8 Hz), 3.88 (d, 2H, *J* = 2.5 Hz), 3.42 (br s, 1H), 1.47 (d, 3H, *J* = 6.7 Hz). *m*/*z* (ESI): 300.0 (M + 1)^+^.

Amide formation according to **B3** between **S4-3** (45 mg, 0.22 mmol) and **(R)-S3-9** was purified by Flash CC (SiO_2_, eluting with 40–100% EtOAc in hexanes). Thereafter, the material was further purified by prep-HPLC (C18 10 μm column, eluting with 10–95% MeCN in H_2_O, using 0.1% TFA as an additive) to yield **18** (28 mg, 48 μmol, 22% yield) as the TFA salt.^1^H NMR (DMSO-*d*_6_, 400 MHz, 340 K) δ 8.7–8.8 (m, 1H), 8.32 (td, 1H, *J* = 1.4, 4.6 Hz), 8.24 (br s, 2H), 7.96 (br s, 2H), 7.7–7.8 (m, 2H), 7.55 (ddd, 1H, *J* = 1.3, 8.4, 10.4 Hz), 7.4–7.5 (m, 1H), 7.35 (td, 1H, *J* = 4.4, 8.5 Hz), 5.3–6.1 (m, 1H), 4.79 (dd, 2H, *J* = 17.5, 19.0 Hz), 3.1–4.2 (m, 2H), 2.34 (s, 3H), 1.62 (br d, 3H, *J* = 6.8 Hz).) ESI-HRMS: calcd for C_25_H_22_F_4_N_5_O: *m*/*z*: [M + H]^+^ = 484.1755; found [M + H]^+^ = 484.1764.

#### (*S*)-2-Amino-*N*-(1-(3-fluoropyridin-2-yl)ethyl)-3-methyl-*N*-((5-(trifluoromethyl)pyridin-2-yl)methyl)quinoline-6-carboxamide (**19**)

##### (*S*)-1-(3-Fluoropyridin-2-yl)-*N*-((5-(trifluoromethyl)pyridin-2-yl)methyl)ethan-1-amine (**(S)-S3-9**)

Reductive amination according to **A2** between 5-(trifluoromethyl)picolinaldehyde and (*S*)-1-(3-fluoropyridin-2-yl)ethan-1-amine was purified by Flash CC (SiO_2_, eluting with 0 to 16% MeOH containing 0.5% ammonium hydroxide in DCM) to yield **(S)-S3-9** (1.1 g, 3.68 mmol, 87% yield) (**1**) as a colorless oil. ^1^H NMR (CDCl_3_, 400 MHz) δ 8.7–8.8 (m, 1H), 8.41 (td, 1H, *J* = 1.3, 4.7 Hz), 7.86 (dd, 1H, *J* = 2.1, 8.2 Hz), 7.52 (d, 1H, *J* = 8.2 Hz), 7.34 (ddd, 1H, *J* = 1.3, 8.3, 9.7 Hz), 7.19 (td, 1H, *J* = 4.2, 8.5 Hz), 4.31 (dq, 1H, *J* = 1.3, 6.8 Hz), 3.8–3.9 (m, 2H), 3.57 (br s, 1H), 1.47 (d, 3H, *J* = 6.7 Hz). *m*/*z* (ESI): 300.0 (M+1)^+^.

Amide formation according to **B3** between **S4-3** (21 mg, 0.104 mmol, LP-126115-4-1) and **(S)-S3-9** was purified first by Flash CC (SiO_2_, eluting with 1–100% MeOH in DCM) followed by prep-HPLC (C18, 10 μm column, eluting with 10–95% MeCN in H_2_O, using 0.1% TFA as an additive) to yield **19** (13 mg, 23 μmol, 22% yield) as the TFA salt. ^1^H NMR (DMSO-*d*_6_, 400 MHz, 360 K) δ 8.7–8.8 (m, 1H), 8.32 (td, 1H, *J* = 1.4, 4.6 Hz), 8.2–8.2 (m, 1H), 8.0–8.2 (m, 1H), 7.97 (dd, 1H, *J* = 1.5, 8.2 Hz), 7.9–7.9 (m, 1H), 7.75 (d, 1H, *J* = 8.5 Hz), 7.69 (d, 1H, *J* = 8.5 Hz), 7.53 (ddd, 1H, *J* = 1.3, 8.4, 10.4 Hz), 7.4–7.4 (m, 1H), 7.33 (td, 1H, *J* = 4.3, 8.5 Hz), 5.6–5.9 (m, 1H), 4.80 (br dd, 2H, *J* = 17.5, 20.5 Hz), 3.1–4.0 (m, 7H), 2.34 (s, 3H), 1.62 (d, 3H, *J* = 6.9 Hz). ESI-HRMS: calcd for C_25_H_22_F_4_N_5_O: *m*/*z*: [M + H]^+^ = 484.1755; found [M + H]^+^ = 484.1761.

#### 2-Amino-3-methyl-*N*-(pyrimidin-2-ylmethyl)-*N*-((5-(trifluoromethyl)pyridin-2-yl)methyl)quinoline-6-carboxamide (**20**)

##### 1-(Pyrimidin-2-yl)-*N*-((5-(trifluoromethyl)pyridin-2-yl)methyl)methanamine (**S3-10**)

Reductive amination according to **A2** between 1-(pyrimidin-2-yl)methanamine and 5-trifluoromethyl-pyridine-2-carbaldehyde was purified with flash CC (SiO_2_, 50% EtOAc in DCM, followed by 2–10% MeOH in DCM) to afford **S3-10** (0.65 g, 2.4 mmol, 42% yield). ^1^H NMR (400 MHz, CD_3_OD) δ 8.8–8.9 (m, 1H), 8.78 (d, 2H, *J* = 5.0 Hz), 8.10 (dd, 1H, *J* = 1.9, 8.2 Hz), 7.69 (d, 1H, *J* = 8.2 Hz,), 7.39 (t, 1H, *J* = 5.0 Hz), 4.10 (s, 2H), 4.09 (s, 2H). *m*/*z* (ESI): 269.0 (M + H)^+^.

Amide formation according to **B2** between **S4-3** and **S3-10** was purified by prep-HPLC (C18, eluting with 10% to 90% CH_3_CN in H_2_O, with 0.1% TFA as an additive) to give **20** (92 mg, 0.17 mmol, 57% yield) as the TFA salt. ^1^H NMR (DMSO-*d*_6_, 400 MHz, 340 K) δ 8.9–8.9 (m, 1H), 8.77 (d, 2H, *J* = 4.9 Hz), 8.2–8.4 (m, 2H), 8.16 (s, 2H), 7.95 (d, 1H, *J* = 1.5 Hz), 7.76 (dd, 1H, *J* = 1.7, 8.5 Hz), 7.6–7.7 (m, 2H), 7.41 (t, 1H, *J* = 4.9 Hz), 4.9–5.0 (m, 2H), 4.8–4.9 (m, 2H), 2.31 (br s, 3H). ESI-HRMS: calcd for C_23_H_20_F_3_N_6_O: *m*/*z*: [M + H]^+^ = 453.1645; found [M + H]^+^ = 453.1649.

#### (*R*)-2-Amino-3-methyl-*N*-(1-(pyrimidin-2-yl)ethyl)-*N*-((5-(trifluoromethyl)pyridin-2-yl)methyl)quinoline-6-carboxamide (**AM-9747**), (*S*)-2-Amino-3-methyl-*N*-(1-(pyrimidin-2-yl)ethyl)-*N*-((5-(trifluoromethyl)pyridin-2-yl)quinoline-6-carboxamide (**21**)

##### 1-(Pyrimidin-2-yl)-*N*-((5-(trifluoromethyl)pyridine-2-yl)methyl)ethan-1-amine (**S3-11**)

Reductive amination according to **A2** between 5-trifluoromethyl-pyridine-2-carbaldehyde and 1-(pyrimidin-2-yl)ethan-1-amine was purified by Flash CC (SiO_2_, 50–80% EtOAc in DCM) to afford **S3-11** (3.13 g, 11.1 mmol, 70% yield) as a brown oil. ^1^H NMR (CD_3_OD, 400 MHz) δ 8.77–8.84 (m, 3H), 8.08 (dd, 1H, *J* = 1.88, 8.15 Hz,), 7.64 (d, 1H, *J* = 8.15 Hz), 7.38 (t, 1H, *J* = 5.02 Hz), 4.07–4.18 (m, 1H), 3.98 (s, 2H), 1.52 (d, 3H, *J* = 6.90 Hz). *m*/*z* (ESI): 283.2 (M + H)^+^.

Amide formation according to **B3** between **S-11** (380 mg, 1.3 mmol) and **S4-3** (330 mg, 1.5 mmol) was purified by Flash CC (SiO_2_, eluting with 50% EtOAc in heptane, followed by 5–8% MeOH in EtOAc) and thereafter Flash CC (C18, 10 to 80% of MeCN in H_2_O, with 0.1% TFA as an additive) gave (*R*,*S*)-2-amino-3-methyl-N-(1-(pyrimidin-2-yl)ethyl)-*N*-((5-(trifluoromethyl)pyridin-2-yl)methyl)quinoline-6-carboxamide as the TFA salt (177 mg, 0.31 mmol, 24% yield). The racemate from above was resolved by prep-SFC (Chiralcel OX, 250 × 30 mm, 5 μm, eluting with 65% CO_2_, 35% MeOH, and 0.2% TEA) yielding **AM-9747** (165 mg, 0.35 mmol, 38% yield) as the first eluting peak and **21** (165 mg, 0.35 mmol, 38% yield) as the second eluting peak.

**AM-9747**^1^H NMR (DMSO-*d*_6_, 400 MHz, 345 K) δ 8.8–8.8 (m, 1H), 8.76 (d, 2H, *J* = 4.8 Hz), 8.06 (dd, 1H, *J* = 2.1, 8.3 Hz), 7.7–7.8 (m, 1H), 7.7–7.7 (m, 1H), 7.5–7.6 (m, 2H), 7.47 (br d, 1H, *J* = 8.6 Hz), 7.36 (t, 1H, *J* = 4.8 Hz), 6.23 (s, 2H), 5.50 (br q, 1H, *J* = 7.2 Hz), 4.94 (d, 1H, *J* = 16.9 Hz), 4.63 (br d, 1H, *J* = 16.9 Hz), 2.24 (s, 3H), 1.62 (d, 3H, *J* = 7.2 Hz).^13^C NMR (DMSO-*d*_6_, 101 MHz, 345 K) δ 171.7, 168.0, 162.9, 158.1, 156.9 (s, 2C), 146.90, 144.94 (q, *J* = 4.1 Hz), 135.15, 133.25 (q, *J* = 3.1 Hz), 128.7, 126.1, 125.0, 124.4, 123.5 (q, *J* = 271.8 Hz), 123.0 (q, *J* = 32.5 Hz), 122.4, 121.0, 120.6, 119.5, 58.7, 48.2, 17.0, 16.8. ESI-HRMS: calcd for C_24_H_22_F_3_N_6_O: *m*/*z*: [M + H]^+^ = 467.1802; found [M + H]^+^ = 467.1805.

**21**^1^H NMR (DMSO-*d*_6_, 400 MHz, 345 K) δ 8.8–8.8 (m, 1H), 8.76 (d, 2H, *J* = 4.9 Hz), 8.06 (dd, 1H, *J* = 2.0, 8.3 Hz), 7.76 (br s, 1H), 7.73 (br s, 1H), 7.5–7.6 (m, 2H), 7.47 (d, 1H, *J* = 8.5 Hz), 7.36 (t, 1H, *J* = 4.9 Hz), 6.25 (s, 2H), 5.4–5.6 (m, 1H), 4.93 (d, 1H, *J* = 16.9 Hz), 4.62 (br d, 1H, *J* = 16.8 Hz), 2.23 (s, 3H), 1.62 (d, 3H, *J* = 7.0 Hz). ESI-HRMS: calcd for C_24_H_22_F_3_N_6_O: *m*/*z*: [M + H]^+^ = 467.1802; found [M + H]^+^ = 467.1807.

### MTase-Glo Assay

The PRMT5 inhibitory activity of test compounds was determined using the MTase-Glo assay (Promega), which monitors the product (S-adenosyl homocysteine or SAH) of methyltransferase reactions. The PRMT5MTase-Glo assay was conducted in a 384-well white ProxiPlate (PerkinElmer) in a total volume of 16 μL. The PRMT5 enzymatic reaction (in 8 μL) contained 4 nM PRMT5:MEP50 (Reaction Biology Corp, catalog no.: RD-11–292), 5 μM SAM (Promega), 2.5 μM FL-Histone H2A (BPS Bioscience, catalog no.: 52021), 1 μM MTA (Sigma) and 1/2 log serially diluted compounds in a reaction buffer of 50 mM Tris (pH 8.0), 50 mM NaCl, 0.01% Tween 20, 0.01% BSA, and 1 mM TCEP. Test compounds were preincubated with PRMT5:MEP50, SAM, and MTA for 1 h before the addition of FL-Histone H2A to initiate the PRMT5 reaction. The reaction was allowed to proceed for 20 h at RT and was then terminated by the addition of 1 μL of 9X MTase-Glo Reagent (Promega). After a 30 min incubation at RT, 8 μL of MTase-Glo Detection Solution (Promega) was added and the plate was incubated at RT for an additional 30 min. The light signal corresponding to the amount of SAH produced by the PRMT5 reaction was subsequently measured by using an Envision multimode reader (PerkinElmer). IC_50_ and K_I_ values were obtained by analyzing dose–response curves using GraphPad Prism. The values reported were an average of *n* ≥ 2, if not otherwise noted.

### HCT116 MTAP-WT and MTAP-del Proliferation Assay

HCT116 MTAP-WT and -del cells (HD PAR-034 and HD R02-033 from Horizon Discovery) were seeded in white-opaque 384-well tissue culture plates (Corning #3570) in RPMI 1640 media +10% fetal bovine serum with 400 cells per well. Plates were thereafter incubated overnight at 37 °C and 5% CO_2_. Cells were then treated with a 14- or 15-point serial dilution of the compound, using a top concentration of 10 μM, employing 1:2 serial dilution steps, and a DMSO-only control using a Tecan D300e. Cells were incubated at 37 °C with 5% CO_2_ in the presence of the compound for 6 days. Effects on cell viability were measured with the CellTiter-Glo Luminescent Cell Viability Assay (Promega #G7571) using the manufacturer’s recommendation (35 μL reagent). Assay plates were read on an EnVision Multilabel Reader using the Ultra-Sensitive luminescence module. IC_50_ values were calculated with GraphPad Prism v 5.01 using a four-parameter symmetrical sigmoidal dose–response least-squares fit. The values reported were an average of *n* ≥ 3 experiments.

### MDCKII-MDR1 Permeability Assay Method A

Permeability study and P-glycoprotein substrate assessment were performed at Fidelta/Selvita employing MDCKII-hMDR1cells (Solvo Biotechnology) with overexpressed human MDR1 gene, coding for P-glycoprotein. They were grown in a controlled atmosphere (37 °C, 95% air, 5% CO_2_, 90% relative humidity) in DMEM with 10% FBS, 1% glutamax-100, 1% MEM Nonessential amino acids, 1% antibiotic/antimycotic. For the permeability experiments, cells were seeded on 96-well insert plates in a final concentration of 0.25 × 10^6^ cells/mL. The cells were fed with fresh medium 24 h post-seeding and cultured for 3 days before use. On the day of the experiment, the cell monolayers were washed and equilibrated with transport medium D-PBS containing 1% of DMSO (with 0.1% BSA, pH = 7.4), without a P-gp inhibitor. Incubation was carried out for 30 min (45 min with elacridar) at 37 °C, 95% humidity. BSA was used to minimize nonspecific binding to plastic labware and was added to the receiver compartment only.

Transport assays were conducted in two directions: apical to basolateral (A-B) and basolateral to apical (B-A), with the following assay controls: amprenavir and talinolol (low permeability and P-gp substrates), diclofenac (high permeability), and Lucifer yellow (cell monolayer integrity control). Each compound was tested in duplicate using the compound’s donor solution consisting of D-PBS with 1% DMSO, Lucifer yellow (100 μM), and the test compound (1 μM). Monolayers were incubated for 120 min at 37 °C with gentle shaking (350 rpm). Apical and basolateral compartments were sampled at the end of the transport experiment. Aliquots were also taken from the donor solution at t = 0, to determine recovery at the end of the experiment.

The compound’s concentrations (the ratio between compound and internal standard peak area) were determined by LC-MS/MS (Nexera X2 UHPLC system (Shimadzu) coupled to a triple quadrupole mass spectrometer API 4000 or API 4500 (AB Sciex)). Instrument control and data acquisition were performed using DiscoveryQuant version 3.0.1 software (AB Sciex).

The apparent permeability *P*_app_ in (×10^–6^ cm s^–1^) was calculated according to the equation:
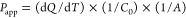


in which d*Q*/d*t* is the permeability rate; C_0_ is the measured initial concentration in the donor compartment; and *A* is the membrane surface area of the cell monolayer. The efflux ratio was calculated between *P*_app_ values from both transport directions (A-B and B-A). Recovery was calculated as % of compound detected in donor and acceptor compartments at the end of transport assay divided by the compound detected in the donor solution at *t* = 0 min.

The efflux ratio in the absence of the P-gp inhibitor was calculated from *P*_app_ values, using the following Equation:
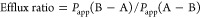


### MDCKII-MDR1 and MDCKII Permeability Assays Method B

P-glycoprotein efflux transport assay was investigated on the human MDR1 overexpressing MDCK cell line (NIH hMDR1-MDCK) commonly used across the industry. It was procured from NIH (Bethesda, MD) and maintained in Dulbecco’s modified Eagle’s medium (DMEM) with 4.5 g/L of glucose and 1 mM or 110 mg/L sodium pyruvate (Invitrogen) supplemented with 10% FBS (Invitrogen), 5 mM l-glutamine (Invitrogen), 50 units/mL penicillin (Invitrogen), 50 ug/mL streptomycin (Invitrogen), and 80 ng/mL colchicine (Sigma). BCRP efflux transport assay was investigated on a human BCRP overexpressing MDCKII cell line (hBCRP-MDCKII). It was engineered using the Flp-In System (Thermo Fisher Scientific) and maintained in DMEM (with Glutamax, 4.5 g/L glucose) containing 10% FBS, 1 mM sodium pyruvate, and 0.1 mM NEAA supplemented with the selection agent hygromycin B (100 μg/mL). For the efflux transport experiments, cells were seeded on 96-well Millicell insert plates (EMD Millipore) at a final density of 0.5 × 10^5^ cells/well (NIH hMDR1-MDCK) or 1 × 10^5^ (hBCRP-MDCKII). Cells were fed with fresh medium daily and maintained at 37 °C, 5% CO2, and 95% humidity for 5 days before use. On the day of the experiment, the cell monolayers were washed and equilibrated with transport buffer HBSS (1X with calcium and magnesium) containing 10 mM HEPES at pH 7.4 and 0.1% BSA.

Transport assays were conducted in two directions: apical to basolateral (A-B) and basolateral to apical (B-A), with the following assay controls: atenolol and labetalol (low permeability and P-gp substrates for NIH hMDR1-MDCK); atenolol and prazosin (low permeability and BCRP substrate for hBCRP-MDCKII). Prior to running the experiment, media was aspirated, and cells were washed twice with transport buffer. A 96-well Millicell feeder/receiver tray (EMD Millipore) was used for basolateral dosing and collection. Briefly, test articles were dosed in the apical chamber when measuring A-B permeability or in the basolateral chamber when measuring B to A permeability. Each compound (1 μM) was tested in triplicate. Monolayers were incubated for 120 min at 37 °C. Apical and basolateral compartments were sampled at the end of the experiment for LC-MS/MS analysis. Aliquots were also taken from the donor solution at *t* = 0, to determine recovery at the end of the experiment. Briefly, samples from transport studies were analyzed on a Nexara LC (Shimadzu) coupled to a Triple Quad 6500 mass spectrometer (Sciex, Redwood City, CA). Sciex 6500 was operated in positive electrospray ionization mode with a source voltage of 5000 V and temperature of 500 °C. Optimal MRM (Multiple Reaction Monitoring) transitions were selected using integrated DiscoveryQuant software (version 3.0.1, Sciex) for each compound. A sample volume of 5 μL was injected using a Shimadzu SIL30ACMP temperature-controlled autosampler. Analytes were separated using a Cadenza CD-C18 column (Imtakt Corp., 30 × 2 mm, 5 mm) maintained at 40 °C. The mobile phase consisted of water with 0.1% formic acid as solvent A and acetonitrile with 0.1% formic acid as solvent B. The flow rate was set at 1.2 mL/min and the gradient was 90% A/10% B for 0.2 min, then ramping up to 1% A/99% B at 0.6 min, holding at 99% B until 0.9 min, and then ramping down to 90% A/10% B at 0.95 min.

The apparent permeability *P*_app_ in (×10^–6^ cm s^–1^) was calculated according to the equation:
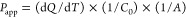
where d*Q*/d*t* is the permeability rate; *C*_0_ is the measured initial concentration in the donor compartment; and *A* is the membrane surface area of the cell monolayer. The efflux ratio was calculated between *P*_app_ values from both transport directions (A-B and B-A). Recovery was calculated as % of compound detected in the donor and acceptor compartments at the end of the transport assay divided by the compound detected in the donor solution at *t* = 0 min.

The efflux ratio was calculated from *P*_app_ values, using the following Equation:
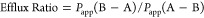


### Metabolic Stability in Liver Microsomes

The metabolic stability of compounds was assessed by Fidelta/Selvita in human and mouse liver microsomes (Corning, Tewksbury, MA, USA). Compounds, in a final concentration of 1 μM, were incubated in phosphate buffer (50 mM, pH 7.4) for 60 min at 37 °C together with liver microsomes and NADPH generating system (nicotinamide adenine dinucleotide phosphate (NADP, 0.5 mM), glucose-6-phosphate (G6P, 5 mM), glucose-6-phosphate dehydrogenase (1.5 U mL^–1^) and magnesium chloride (0.5 mM)). Also, compounds were incubated without the presence of the NADPH cofactor, as a buffer stability control. The metabolic activity of liver microsomes was verified by including testosterone and propranolol as positive controls and caffeine as a negative control. Sampling was performed at six time points (0, 10, 20, 30, 45, and 60 min), followed by reaction termination by the addition of an acetonitrile/methanol mixture (2:1, *v/v*) containing diclofenac as an internal standard. Samples were analyzed by LC-MS/MS as previously mentioned for the MDCKII-MDR1 permeability assay.

Metabolic stability, expressed as the percentage of the remaining parent compound, was calculated from the ratio of the peak area of the remaining compound and the peak area of the analytical internal standard, after different times of incubation compared to the same ratio at time 0 min (100%). The in vitro half-life (*t*_1/2_) was calculated in GraphPadPrism software from % remaining vs time regression using nonlinear regression fit (one phase exponential decay) with the following constrain parameters: Span = 100, Plateau = 0, K = no constraint).

In vitro intrinsic clearance, CL_int_ was calculated from half-life using the following equation:
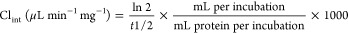
where 52.5 mg protein/g liver is used as a constant. Predicted in vivo hepatic clearance, in vivo CL_h_ was calculated as follows:
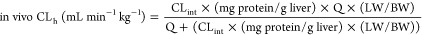
where: LW/BW = liver weight/body weight (g kg^–1^); 25.7 (human), 87.5 (mouse); Q = LBF = liver blood flow (mL min^–1^ kg^–1^); 21 (human), 131 (mouse). Predicted in vivo hepatic clearance can be expressed as %LBF and calculated as follows:
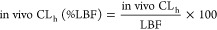


### HCT116 SDMA Imaging Assays

HCT116 cells were seeded at 1500 cells/well (for MTAP-WT) and 2000 cells/well (for MTAP-del) in a 96-well imaging plate, at a volume of 90 mL per well, and then incubated overnight at 37 °C. The next day, the cells were treated with a 9-point serial dilution of the compound with a top concentration of 1 μM for MTAP-WT cells and 0.1 μM for MTAP-del cells, respectively, followed by eight 1:3 serial dilution steps and a DMSO-only control. Cells were then returned to the incubator at 37 °C and 5% CO_2_ for 3 days. Media was then removed from each well, and the cells were fixed by adding 100 mL of formaldehyde (4%) and incubated for 15 min at RT. After fixation, the cells were permeabilized in 100 mL of Wash Buffer (1% BSA, 0.2% Triton X-100, 1 × PBS) for 15 min at RT. Wash Buffer was then replaced with 100 mL per well of Blocking Buffer (2 drops of horse serum (Vector Lab, Burlingame, CA) in 5 mL Wash Buffer) and incubated for 1 h at RT. Cells were then stained with an anti-SDMA polyclonal antibody (CST#13222S at 1:2000 dilution) in a wash buffer and incubated at RT for 2 h. Cells were washed with 200 mL of Wash Buffer and stained with secondary antibodies (goat antirabbit-alexa-488 Cat #A11034, Invitrogen at 1:2000 dilution) and Hoechst 33342 DNA dye (1:5000 dilution) for 1 h at RT in the dark. Cells were washed with 150 mL of Wash Buffer 3 times. Plates were sealed (PerkinElmer, Waltham, MA), and imaging data was acquired using Cellomics ArrayScan VTI HCS Reader (SN03090745F, Thermo Fisher Scientific) running the SpotDetector.V4 Assay protocol (Ver 6.6.0 (Build 8153) with a 10X objective, 16-fields per well). The data outputs include: (1) Valid Object Count (Channel 1. Hoechst 33342 DNA dye). This represents the total valid nuclear object count per well (based on Object. Area and Object. VarIntensity were used to set the range for valid objects, objects outside this range were rejected). (2) EventType1 ObjectCount (channel 2. SDMA/Alexa-488 antibody). This represents the total valid SDMA positive object count based on the set fluorescence intensity threshold. (3) % EventType1 Objects. This represents the percentage of SDMA positive objects [(EventType1ObjectCount (2)/Valid Object Count (1)) × 100]. (4) MEAN_AvgintenCh2. This represents the mean average fluorescence intensity of channel 2 for the valid SDMA positive object count. Percent of control (POC) values were calculated from the Mean Average Fluorescence Intensity as follows: POC = 100 × (Treatment/Vehicle). Mean POC values were then calculated for each treatment condition and used to fit the dose–response curves by applying a four-parameter logistic curve in the GraphPad Prism software.

### Ethics Statement

All animal experimental protocols were approved by the Amgen Institutional Animal Care and Use Committee (IACUC) and were conducted in accordance with the guidelines set by the Association for Assessment and Accreditation of Laboratory Animal Care (AAALAC). Mice were housed in an environmentally controlled room (temperature 23 ± 2 °C, relative humidity 50 ± 20%) on a 12 h light/dark cycle. Mice were fed commercial rodent chow and water ad libitum. Mice with a tumor size exceeding 2,000 mm^3^ were removed from the study and euthanized.

### In Vivo Pharmacology

Mice were housed in sterilized filter-capped cages and maintained under aseptic and pathogen-free conditions. All studies utilized 4- to 8-week-old female Athymic Nude, female CB17 SCID mice (Charles River Laboratories), or female NOD/SCID mice (Jackson Laboratories). **AM-9747** was formulated in 2% hydroxypropyl methylcellulose and 1% Tween-80 at pH 2.0. **AM-9747** was stored at 2–8 °C and protected from light. **AM-9747** was mixed well prior to PO administration.

Tumor dimensions were assessed twice weekly with a Pro-Max electronic digital caliper (Japan Micrometer Mfg. Co. LTD), and tumor volume was calculated using the formula: length x width x height and expressed as mm^3^ using StudyDirector. Body weight was measured twice per week using an analytical laboratory scale.

The percentage of tumor growth inhibition (%TGI) was calculated as %TGI relative to the vehicle alone: %TGI = 100 – [(Treated – Initial Volume)/(Control – Initial Volume) × 100]. The percentage of tumor regression (%TR) was calculated as %TR compared final tumor volume to the initial tumor volume: %TR = 100 – [(Final Volume)/(Initial Volume) × 100]. Tumor efficacy data expressed as mean tumor volume ± standard error of the mean (SEM) for each group were plotted as a function of time (days).

### HCT116 MTAP-WT, and -del Pharmacodynamic Assay

Athymic Nude mice were implanted bilaterally with HCT116 MTAP-WT and HCT 116 MTAP-del cells (2.0 × 10^06^ with Matrigel). Animals with established tumors were sorted into groups (*n* = 5) with similar tumor volumes. Animals were administered vehicle or **AM-9747** (1, 3, 10, and 30 mg/kg) orally once a day for 4 days. Tumor and blood plasma samples were collected 4 h after the fourth dose and processed for PD (SDMA) or PK (plasma, tumor). For SDMA analysis see below process: SDMA ELISA. Pharmacodynamic and pharmacokinetic data were plotted on the same graph and expressed as mean plus SEM using GraphPad Prism 7.05 software. Statistical significance was computed versus matched control by an Ordinary one-way ANOVA at a significance level of 0.05 with Dunnett’s multiple comparisons test.

### CDX Studies

DOHH-2 (1.0 × 10^07^) and Bx-PC3 (5.0 × 10^06^ with Matrigel) cells were injected subcutaneously in the right flank of female CB17 SCID mice (*N* = 10/group). HCT116 MTAP-WT or HCT116 MTAP-del (2.0 × 10^06^ with Matrigel), cells were injected subcutaneously in the right flank of female Athymic Nude mice (*N* = 10/group). Treatment began when tumors were ∼171 mm^3^ (HCT116 MTAP-WT), ∼171 mm^3^ (HCT116 MTAP-del), ∼155 mm^3^ (DOHH-2), and ∼120 mm^3^ (Bx-PC3). In the dose–response studies, mice received oral doses of either vehicle (QD) or **AM-9747** (3, 10, 30, 100 mg/kg QD for HCT116 MTAP-WT and HCT116 MTAP-del; 10, 30, and 1000 mg/kg QD for DOHH-2; 10, 30, and 100 mg/kg QD for Bx-PC3. Tumor dimensions were assessed twice weekly with a Pro-Max electronic digital caliper (Japan Micrometer Mfg. Co. LTD), and tumor volume was calculated using the formula: length × width × height and expressed as mm^3^ using StudyDirector. Data are expressed as the mean ± SEM.

### PDX Studies

PA5415 pancreatic and ES11082 esophageal PDX studies were performed by Crown Biosciences (San Diego, CA). NOD/SCID mice were implanted subcutaneously with 2 × 2 mm tumor chunks. Mice were randomized when tumor volumes were ∼100–150 mm^3^ (*N* = 10/group) and received either vehicle (QD) or **AM-9747** (100 mg/kg QD) after randomization. Tumor dimensions were calculated twice weekly using the formula: width × length × height and expressed in mm^3^.

### SDMA ELISA Assay

The SDMA inhibition level in the terminal tumor was assessed using an ELISA assay. Tumors from dose–response CDX studies were collected and stored in a Covaris Tissue Tube (520001 TT1) to be used in conjunction with the CryoPrep. Tumor samples were snap-frozen in liquid nitrogen and placed on dry ice. Prior to pulverization, a transfer tube (520010 TC13 13 × 65 mm tube [glass]) was attached to the top TT1 tube assembly and then placed into the CryoPrep where the sample was pulverized using a hammer and anvil mechanism. The pulverized sample was then transferred into the glass tube and placed on dry ice. The sample was then lysed using RIPA buffer (50 mM Tris-HCl, pH7.5, 1% Igepal, 0.5% sodium deoxycolate, 150 mM NaCl, 0.1% SDS) supplemented with 1× HALT phosphatase and phosphatase inhibitor. Then, freshly diluted 1× lysis buffer was added at 1 mL/sample tissue, and previously pulverized tumor tissue was resuspended. Samples were lysed on ice for 30 min. The suspension was transferred to a 2 mL Eppendorf tube and then centrifuged at 14,000 rpm at 4 °C for 10 min. The supernatant was then transferred into a new 2 mL Eppendorf tube. The protein concentration of each lysate was then determined using a BCA assay. Lysates were normalized for total protein/well and loaded onto MSD plates at 480 ng/well in lysis buffer for the SDMA ELISA plate. 100 μL of lysates (480 ng/well in duplicates) were transferred to the 96-well high binding plates; plates were then shaken for 2 h at RT (RT) and then washed 4 times with 200 μL 1× PBS 0.1% Tween 20 Wash Buffer. SuperBlock Solution (Thermo Scientific) was added 150 μL per well into the SDMA plates. Plates were then shaken for 2 h at RT (RT) and then washed 4 times with 200 mL 1× PBS 0.1% Tween 20 Wash Buffer. Then 100 μL of the anti-sDMA antibody diluted 1:2000 in 1× PBST + 1% BSA was added to the wells and incubated covered overnight at 4 degrees. Plates were washed 4 times with 200 μL 1× PBS 0.1% Tween 20 Wash Buffer and then 100 mL per well of secondary antibody (antirabbit IgG horseradish peroxidase conjugate, 1:4000 diluted in 1× PBST + 1% BSA) was added and incubated covered for 1 h at RT (RT) while shaking. Plates were washed 4 times with 200 μL 1× PBS 0.1% Tween 20 Wash Buffer and then 100 μL per well of luminata Read Buffer was added and incubated covered for 15 min before each plate was immediately analyzed on Envision reader (PerkinElmer, Waltham, MA) for luminescence detection. Because the lysates were normalized for total protein, the signals were then used to show the treatment group levels SDMA protein inhibition level compared to the vehicle group in 480 ng of a given tumor sample.

### Measurement of **AM-9747** in Plasma and Tumor Samples

Blank tumors and tumors collected from the PD study were homogenized in DI water at a ratio of 4 mL water per 1 g tumor using Precellys bead-based homogenizer at 5500 × 20 s for 3 cycles. **AM-9747** was spiked into blank mouse plasma or blank tumor homogenate to generate the calibration curve samples. Plasma and tumor homogenate samples from high-dose groups were further diluted with a blank matrix before quenching in order to be quantified within the linear range. Twenty-five μL of plasma or tumor homogenate samples or calibration curve samples were added with 200 μL of quench solvent which consisted of 10 ng/mL internal standard (IS, Amgen internal compound) in acetonitrile. The mixture was vortexed at 1300 rpm for 15 min and then centrifuged at 4500 rpm for 15 min. The supernatant was transferred to a new plate and subjected to liquid chromatography–selected reaction monitoring mass spectrometry (LC-SRM MS) analysis. Chromatographic separation was performed using a Phenomenex kinetex C18 column (2.0 × 50 mm; 1.7 μm; column temperature maintained at 40 °C) on a Shimadzu Nexera system. Solvent A contained 0.1% formic acid in water; solvent B contained 0.1% formic acid in acetonitrile. The flow rate was 400 μL/min, and the gradient was set as follows: 5%B from 0 to 0.5 min, ramped up to 95%B from 0.5 to 2.2 min, maintained at 95%B from 2.2 to 2.8 min, and then ramped down to 5%B at 2.9 min for equilibration. Detection and quantitation of AM-9747 were carried out on a QTRAP 6500 mass spectrometer (Sciex) equipped with an electrospray ionization source, operating in positive ionization mode. For SRM MS analysis, the following transitions were monitored: AM-9747 (467.1.0/185.0), IS (604.2/268.3). Data was analyzed using the Quantitate module embedded in Analyst 1.7 (Sciex). Peaks were integrated and the peak area ratios of analyte to internal standard were used for quantitation. Linear regression (1/×2 weighting, *r*2 > 0.99) was achieved with LLOQ of 0.3 ng/mL and ULOQ of 5000 ng/mL for both plasma and tumor homogenate samples.

### Statistical Analysis

For in vitro studies, statistical analysis was performed using GraphPad Prism (GraphPad). Unless noted, data are presented as the mean and SE. One-way ANOVA, two-way ANOVA, Pearson correlation coefficient, and unpaired *t-*test were used as appropriate. *P*-values were considered significant at *, *P* < 0.05; **, *P* < 0.01; ***, *P* < 0.001; and ****, *P* < 0.0001. For animal studies, statistical analysis was performed on treatment groups relative to vehicle alone using Linear Mixed Effects 931 Models implemented within the custom-developed application IVEA (v2.01.00.01) using R 4.1.1 (cran.r-project.org 932and nlme 3.1.153) package with Dunnett’s correction applied for multiplicity. Statistical significance was reported with a *P* value of ≤ 0.05, otherwise considered as not significant (ns).

## Supporting Information Available

Detailed descriptions of **DEL91**, solid and solution phase synthesis of **5**, molecular modeling details, protein expression, and purification, data collection and refinement statistics, SDMA levels after repeated (21 day) dosing, Eurofins (Cerep) and DiscoverX Kinime targets tested, *c*Log *P* and UPLC-UV of final compounds, ^1^H NMR of **AM-9934**, and ^1^H NMR, ^13^C NMR, COSY, and NOESY of **AM-9747** (PDF)
Molecular formulation strings (CSV)
Complex of AM-9747+PRMT5:MEP50+MTA (9MRE); assigned Arabic number for ligand ID: A1BQW, and authors will release the atomic coordinates upon article publication (PDB)

## Supplementary Material

jm4c03101_si_002.csv

jm4c03101_si_003.pdb

## Dedication

^#^ Passed away February 22nd, 2023.

## Abbreviations Used

BB: building block
BCRP: breast cancer resistance protein
CDKN2A: cyclin-dependent kinase inhibitor 2A
CDX: cell derived xenograft
CL: clearance
CLND: chemiluminescent nitrogen detection
DEL: DNA-encoded library
DEL-SAFIR: preliminary structure affinity relationship based upon the DEL output
DCM: dichloromethane
DIPEA: *N*,*N*-diisopropylethylamine
DMTMM: 4-(4,6-dimethoxy-1,3,5-triazin-2-yl)-4-methylmorpholinium
DMF: dimethylformamide
DMAc: dimethylacetamide
EDC: 1-ethyl-3-(3-(dimethylamino)propyl)carbodiimide
EWG: electron withdrawing group
F%: percent bioavailability
HATU: hexafluorophosphate azabenzotriazole tetramethyl uronium
HOAt: 1-hydroxy-7-azabenzotriazole
HPMC: hydroxypropyl methylcellulose
IV: intra venous
LHS: left-hand side
MTA: methylthioadenosine
MDR1: multidrug resistance protein 1
MEP50: methylosome protein 50
MDCK: Madin–Darby canine kidney
MS: mass spectrometry
MTAP-del/MTAP null: methylthioadenosine phosphorylase deleted
MTAP-WT: methylthioadenosine phosphorylase wild type
ND: not determined
NOD/SCID: nonobese diabetic/severe combined immunodeficiency
PCR: polymerase chain reaction
PD: pharmacodynamic
PDX: patient derived xenograft
POS: position
PRMT5: protein arginine methyltransferase 5
PSA: polar surface area
PyBroP: bromotripyrrolidinophosphonium hexafluorophosphate
Q2A: quinolin-2-amine
QD: once daily
RHS: right-hand side
RT: room temperature
SAM: S-adenosylmethionine
SAR: structure activity relationship
SDMA: symmetric dimethylation of arginine
SNAr: nucleophilic aromatic substitution
TFA: trifluoroacetic acid
TGI: tumor growth inhibition
TR: tumor regression
TEA: triethylamine
UPLC: ultraperformance liquid chromatography
Vdss: volume of distribution at the solid state

## References

[ref1] Wu Q.; Schapira M.; Arrowsmith C. H.; Barsyte-Lovejoy D. Protein arginine methylation: from enigmatic functions to therapeutic targeting. Nat. Rev. Drug Discov 2021, 20 (7), 509–530. 10.1038/s41573-021-00159-8. 33742187

[ref2] Peng C.; Wong C. C. The story of protein arginine methylation: characterization, regulation, and function. Expert Rev. Proteomics 2017, 14 (2), 157–170. 10.1080/14789450.2017.1275573. 28043171

[ref3] Fuhrmann J.; Clancy K. W.; Thompson P. R. Chemical biology of protein arginine modifications in epigenetic regulation. Chem. Rev. 2015, 115 (11), 5413–5461. 10.1021/acs.chemrev.5b00003. 25970731 PMC4463550

[ref4] Blanc R. S.; Richard S. Arginine Methylation: The Coming of Age. Mol. Cell 2017, 65 (1), 8–24. 10.1016/j.molcel.2016.11.003. 28061334

[ref5] Guccione E.; Richard S. The regulation, functions and clinical relevance of arginine methylation. Nat. Rev. Mol. Cell Biol. 2019, 20 (10), 642–657. 10.1038/s41580-019-0155-x. 31350521

[ref6] Musiani D.; Bok J.; Massignani E.; Wu L.; Tabaglio T.; Ippolito M. R.; Cuomo A.; Ozbek U.; Zorgati H.; Ghoshdastider U.; et al. Proteomics profiling of arginine methylation defines PRMT5 substrate specificity. Sci. Signal. 2019, 12 (575), eaat8388 10.1126/scisignal.aat8388. 30940768

[ref7] Antonysamy S.; Bonday Z.; Campbell R. M.; Doyle B.; Druzina Z.; Gheyi T.; Han B.; Jungheim L. N.; Qian Y.; Rauch C.; et al. Crystal structure of the human PRMT5:MEP50 complex. Proc. Natl. Acad. Sci. U. S. A. 2012, 109 (44), 17960–17965. 10.1073/pnas.1209814109. 23071334 PMC3497828

[ref8] Kalev P.; Hyer M. L.; Gross S.; Konteatis Z.; Chen C. C.; Fletcher M.; Lein M.; Aguado-Fraile E.; Frank V.; Barnett A.; et al. MAT2A Inhibition Blocks the Growth of MTAP-Deleted Cancer Cells by Reducing PRMT5-Dependent mRNA Splicing and Inducing DNA Damage. Cancer Cell 2021, 39 (2), 209–224.e211. 10.1016/j.ccell.2020.12.010. 33450196

[ref9] Kryukov G. V.; Wilson F. H.; Ruth J. R.; Paulk J.; Tsherniak A.; Marlow S. E.; Vazquez F.; Weir B. A.; Fitzgerald M. E.; Tanaka M.; et al. MTAP deletion confers enhanced dependency on the PRMT5 arginine methyltransferase in cancer cells. Science 2016, 351 (6278), 1214–1218. 10.1126/science.aad5214. 26912360 PMC4997612

[ref10] Marjon K.; Cameron M. J.; Quang P.; Clasquin M. F.; Mandley E.; Kunii K.; McVay M.; Choe S.; Kernytsky A.; Gross S.; et al. MTAP Deletions in Cancer Create Vulnerability to Targeting of the MAT2A/PRMT5/RIOK1 Axis. Cell Rep 2016, 15 (3), 574–587. 10.1016/j.celrep.2016.03.043. 27068473

[ref11] Mavrakis K. J.; McDonald E. R. 3rd; Schlabach M. R.; Billy E.; Hoffman G. R.; deWeck A.; Ruddy D. A.; Venkatesan K.; Yu J.; McAllister G.; et al. Disordered methionine metabolism in MTAP/CDKN2A-deleted cancers leads to dependence on PRMT5. Science 2016, 351 (6278), 1208–1213. 10.1126/science.aad5944. 26912361

[ref12] Zheng J.; Li B.; Wu Y.; Wu X.; Wang Y. Targeting Arginine Methyltransferase PRMT5 for Cancer Therapy: Updated Progress and Novel Strategies. J. Med. Chem. 2023, 66 (13), 8407–8427. 10.1021/acs.jmedchem.3c00250. 37366223

[ref13] Wu Y.; Wang Z.; Zhang J.; Ling R. Elevated expression of protein arginine methyltransferase 5 predicts the poor prognosis of breast cancer. Tumour Biol. 2017, 39 (4), 1010428317695917 10.1177/1010428317695917. 28381188

[ref14] Tan L.; Xiao K.; Ye Y.; Liang H.; Chen M.; Luo J.; Qin Z. High PRMT5 expression is associated with poor overall survival and tumor progression in bladder cancer. Aging (Albany NY) 2020, 12 (9), 8728–8741. 10.18632/aging.103198. 32392182 PMC7244052

[ref15] Jing P.; Zhao N.; Ye M.; Zhang Y.; Zhang Z.; Sun J.; Wang Z.; Zhang J.; Gu Z. Protein arginine methyltransferase 5 promotes lung cancer metastasis via the epigenetic regulation of miR-99 family/FGFR3 signaling. Cancer Lett. 2018, 427, 38–48. 10.1016/j.canlet.2018.04.019. 29679612

[ref16] Chen H.; Lorton B.; Gupta V.; Shechter D. A TGFbeta-PRMT5-MEP50 axis regulates cancer cell invasion through histone H3 and H4 arginine methylation coupled transcriptional activation and repression. Oncogene 2017, 36 (3), 373–386. 10.1038/onc.2016.205. 27270440 PMC5140780

[ref17] Barczak W.; Jin L.; Carr S. M.; Munro S.; Ward S.; Kanapin A.; Samsonova A.; La Thangue N. B. PRMT5 promotes cancer cell migration and invasion through the E2F pathway. Cell Death Dis 2020, 11 (7), 572 10.1038/s41419-020-02771-9. 32709847 PMC7382496

[ref18] Duncan K. W.; Rioux N.; Boriack-Sjodin P. A.; Munchhof M. J.; Reiter L. A.; Majer C. R.; Jin L.; Johnston L. D.; Chan-Penebre E.; Kuplast K. G.; et al. Structure and Property Guided Design in the Identification of PRMT5 Tool Compound EPZ015666. ACS Med. Chem. Lett. 2016, 7 (2), 162–166. 10.1021/acsmedchemlett.5b00380. 26985292 PMC4753547

[ref19] Duncan K. W.; Chesworth R.; Boriack-Sjodin P. A.; Munchhof M. J.; Jin L. PRMT5 inhibitors and uses thereof. US8993555B2, 2015.

[ref20] Chan-Penebre E.; Kuplast K. G.; Majer C. R.; Boriack-Sjodin P. A.; Wigle T. J.; Johnston L. D.; Rioux N.; Munchhof M. J.; Jin L.; Jacques S. L.; et al. A selective inhibitor of PRMT5 with in vivo and in vitro potency in MCL models. Nat. Chem. Biol. 2015, 11 (6), 432–437. 10.1038/nchembio.1810. 25915199

[ref21] Brehmer D.; Beke L.; Wu T.; Millar H. J.; Moy C.; Sun W.; Mannens G.; Pande V.; Boeckx A.; van Heerde E.; et al. Discovery and Pharmacological Characterization of JNJ-64619178, a Novel Small-Molecule Inhibitor of PRMT5 with Potent Antitumor Activity. Mol. Cancer Ther 2021, 20 (12), 2317–2328. 10.1158/1535-7163.MCT-21-0367. 34583982 PMC9398174

[ref22] Siu L. L.; Rasco D. W.; Vinay S. P.; Romano P. M.; Menis J.; Opdam F. L.; Heinhuis K. M.; Egger J. L.; Gorman S. A.; Parasrampuria R.; et al. METEOR-1: A phase I study of GSK3326595, a first-in-class protein arginine methyltransferase 5 (PRMT5) inhibitor, in advanced solid tumours. Ann. Oncol. 2019, 30, v159 10.1093/annonc/mdz244.

[ref23] Rodon Ahnert J.; Perez C. A.; Wong K. M.; Maitland M. L.; Tsai F.; Berlin J.; Liao K. H.; Wang I. M.; Markovtsova L.; Jacobs I. A.; et al. PF-06939999, a potent and selective PRMT5 inhibitor, in patients with advanced or metastatic solid tumors: A phase 1 dose escalation study. J. Clin. Oncol. 2021, 39 (15_suppl), 3019–3019. 10.1200/JCO.2021.39.15_suppl.3019.

[ref24] Hoffman J. L. Chromatographic analysis of the chiral and covalent instability of S-adenosyl-L-methionine. Biochemistry 1986, 25 (15), 4444–4449. 10.1021/bi00363a041. 3530324

[ref25] Subhi A. L.; Diegelman P.; Porter C. W.; Tang B.; Lu Z. J.; Markham G. D.; Kruger W. D. Methylthioadenosine phosphorylase regulates ornithine decarboxylase by production of downstream metabolites. J. Biol. Chem. 2003, 278 (50), 49868–49873. 10.1074/jbc.M308451200. 14506228

[ref26] Karkhanis V.; Hu Y. J.; Baiocchi R. A.; Imbalzano A. N.; Sif S. Versatility of PRMT5-induced methylation in growth control and development. Trends Biochem. Sci. 2011, 36 (12), 633–641. 10.1016/j.tibs.2011.09.001. 21975038 PMC3225484

[ref27] Friesen W. J.; Wyce A.; Paushkin S.; Abel L.; Rappsilber J.; Mann M.; Dreyfuss G. A novel WD repeat protein component of the methylosome binds Sm proteins. J. Biol. Chem. 2002, 277 (10), 8243–8247. 10.1074/jbc.M109984200. 11756452

[ref28] Pettus L. H.; Tamayo N. A.; Bourbeau M. P.; Beylkin D.; Booker S. K.; Butler J.; Kaller M. R.; Kohn T. J.; Lanman B. A.; Li K.; . Discovery of AMG 193, an MTA-Cooperative PRMT5 Inhibitor for the Treatment of MTAP-Null Cancers. unpublished results 2024. 10.1021/acs.jmedchem.4c03121 40146197

[ref29] Smith C. R.; Aranda R.; Bobinski T. P.; Briere D. M.; Burns A. C.; Christensen J. G.; Clarine J.; Engstrom L. D.; Gunn R. J.; Ivetac A.; et al. Fragment-Based Discovery of MRTX1719, a Synthetic Lethal Inhibitor of the PRMT5*MTA Complex for the Treatment of MTAP-Deleted Cancers. J. Med. Chem. 2022, 65 (3), 1749–1766. 10.1021/acs.jmedchem.1c01900. 35041419

[ref30] Cottrell K. M.; Briggs K. J.; Whittington D. A.; Jahic H.; Ali J. A.; Davis C. B.; Gong S.; Gotur D.; Gu L.; McCarren P.; et al. Discovery of TNG908: A Selective, Brain Penetrant, MTA-Cooperative PRMT5 Inhibitor That Is Synthetically Lethal with MTAP-Deleted Cancers. J. Med. Chem. 2024, 67 (8), 6064–6080. 10.1021/acs.jmedchem.4c00133. 38595098 PMC11056935

[ref31] Briggs K. J.; Tsai A.; Zhang M.; Tonini M. R.; Haines B.; Huang A.; Cottrell K. M. TNG462 is a potential best-in-class MTA-cooperative PRMT5 inhibitor for the treatment of MTAP-deleted solid tumors. AACR Cancer Res. 2023, 2023, 4970 10.1158/1538-7445.AM2023-4970.

[ref32] https://www.cancer.gov/research/participate/clinical-trials/intervention/prmt5-inhibitor-azd3470 (accessed Jan 21, 2025).

[ref33] Smith J. M.; Barlaam B.; Beattie D.; Bradshaw L.; Chan H. M.; Chiarparin E.; Collingwood O.; Cooke S. L.; Cronin A.; Cumming I.; et al. Discovery and In Vivo Efficacy of AZ-PRMT5i-1, a Novel PRMT5 Inhibitor with High MTA Cooperativity. J. Med. Chem. 2024, 67 (16), 13604–13638. 10.1021/acs.jmedchem.4c00097. 39080842

[ref34] Gironda-Martinez A.; Donckele E. J.; Samain F.; Neri D. DNA-Encoded Chemical Libraries: A Comprehensive Review with Succesful Stories and Future Challenges. ACS Pharmacol Transl Sci. 2021, 4 (4), 1265–1279. 10.1021/acsptsci.1c00118. 34423264 PMC8369695

[ref35] Andersson J.; Cowland S.; Vestergaard M.; Yang Y.; Liu S.; Fang X.; Mukund S.; Ghimire-Rijal S.; Jacso T.; Sarvary I.; . Discovery of MTA-cooperative PRMT5 inhibitors from co-factor directed DNA-Encoded Library screens. unpublished results 2024. 10.1073/pnas.2425052122 PMC12107103 40377999

[ref36] Goodnow R. A. Jr.; Dumelin C. E.; Keefe A. D. DNA-encoded chemistry: enabling the deeper sampling of chemical space. Nat. Rev. Drug Discov 2017, 16 (2), 131–147. 10.1038/nrd.2016.213. 27932801

[ref37] Su W.; Ge R.; Ding D.; Chen W.; Wang W.; Yan H.; Wang W.; Yuan Y.; Liu H.; Zhang M.; et al. Triaging of DNA-Encoded Library Selection Results by High-Throughput Resynthesis of DNA-Conjugate and Affinity Selection Mass Spectrometry. Bioconjug Chem. 2021, 32 (5), 1001–1007. 10.1021/acs.bioconjchem.1c00170. 33914520

[ref38] Xia B.; Franklin G. J.; Lu X.; Bedard K. L.; Grady L. C.; Summerfield J. D.; Shi E. X.; King B. W.; Lind K. E.; Chiu C.; et al. DNA-Encoded Library Hit Confirmation: Bridging the Gap Between On-DNA and Off-DNA Chemistry. ACS Med. Chem. Lett. 2021, 12 (7), 1166–1172. 10.1021/acsmedchemlett.1c00156. 34267887 PMC8274064

[ref39] Hitchcock S. A. Structural modifications that alter the P-glycoprotein efflux properties of compounds. J. Med. Chem. 2012, 55 (11), 4877–4895. 10.1021/jm201136z. 22506484

[ref40] Shah P.; Westwell A. D. The role of fluorine in medicinal chemistry. J. Enzyme Inhib Med. Chem. 2007, 22 (5), 527–540. 10.1080/14756360701425014. 18035820

[ref41] Talele T. T. The ″Cyclopropyl Fragment″ is a Versatile Player that Frequently Appears in Preclinical/Clinical Drug Molecules. J. Med. Chem. 2016, 59 (19), 8712–8756. 10.1021/acs.jmedchem.6b00472. 27299736

[ref42] Bauer M. R.; Di Fruscia P.; Lucas S. C. C.; Michaelides I. N.; Nelson J. E.; Storer R. I.; Whitehurst B. C. Put a ring on it: application of small aliphatic rings in medicinal chemistry. RSC Med. Chem. 2021, 12 (4), 448–471. 10.1039/D0MD00370K. 33937776 PMC8083977

[ref43] Belmontes B.; Slemmons K. K.; Su C.; Liu S.; Policheni A. N.; Moriguchi J.; Tan H.; Xie F.; Aiello D. A.; Yang Y.; et al. AMG 193, a Clinical Stage MTA-Cooperative PRMT5 Inhibitor, Drives Antitumor Activity Preclinically and in Patients With MTAP-Deleted Cancers. Cancer Discov 2025, 15, 139 10.1158/2159-8290.CD-24-0887. 39282709 PMC11726016

[ref44] Engstrom L. D.; Aranda R.; Waters L.; Moya K.; Bowcut V.; Vegar L.; Trinh D.; Hebbert A.; Smith C. R.; Kulyk S.; et al. MRTX1719 Is an MTA-Cooperative PRMT5 Inhibitor That Exhibits Synthetic Lethality in Preclinical Models and Patients with MTAP-Deleted Cancer. Cancer Discovery 2023, 13 (11), 2412–2431. 10.1158/2159-8290.CD-23-0669. 37552839 PMC10618744

[ref45] Liu Y.; Wu W.; Cai C.; Zhang H.; Shen H.; Han Y. Patient-derived xenograft models in cancer therapy: technologies and applications. Signal Transduct Target Ther 2023, 8 (1), 160 10.1038/s41392-023-01419-2. 37045827 PMC10097874

[ref46] Xin Z.; Gogsig T. M.; Lindhardt A. T.; Skrydstrup T. An efficient method for the preparation of tertiary esters by palladium-catalyzed alkoxycarbonylation of aryl bromides. Org. Lett. 2012, 14 (1), 284–287. 10.1021/ol203057w. 22168208

[ref47] Allen J. R.; Amegadzie A.; Beylkin D. J.; Booker S.; Bourbeau M. P.; Butler J. R.; Frohn M. J.; Glad S. O. S.; Husemoen B. W.; Kaller M. R. Novel PRMT5 Inhibitors. WO2021/163344A1, 2021.

[ref48] Pendyala Satya Kishore R. G.; Rama Kishore Putta V. P.; Polina S.; Singh V.; Malakar C. C.; Pujar P. P. Potassium tert-Butoxide-Mediated Synthesis of 2-Aminoquinolines from Alkylnitriles and 2-Aminobenzaldehyde Derivatives. ChemistrySelect 2022, 7, e202204238 10.1002/slct.202204238.

