# Supplementary material for: From DNA-Encoded Library Screening to AM-9747: An MTA-Cooperative PRMT5 Inhibitor with Potent Oral In Vivo Efficacy
Source: J Med Chem. 2025 Mar 18;68(6):6534–57. doi: 10.1021/acs.jmedchem.4c03101 (PMC11956014; doi:10.1021/acs.jmedchem.4c03101)
Supplement: Supplementary file 1 — jm4c03101_si_001.pdf [file jm4c03101_si_001.pdf]

SUPPORTING INFORMATION

From DNA-Encoded Library Screening to AM-9747 - an MTA-Cooperative PRMT5 Inhibitor with Potent Oral *in vivo* Efficacy.

Ian Sarvary [a*], Mikkel Vestergaard[a], Loris Moretti [a], Jan Andersson[a], Jorge Peiró Cadahía[a], Sanne Cowland[a], Thomas Flagstad[a], Thomas Franch[a], Alex Gouliaev[a], Gitte Husemoen[a], Tomas Jacso[a], Titi Kronborg[b], Aleksandra Kuropatnicka[a], Anna Nadali[a], Mads Madsen[a], Søren Nielsen[a], David Pii[b], Søren Ryborg[a], Camillia Soede[b], Jennifer R. Allen[c], Matthew Bourbeau[c], Kexue Li[c†], Qingyian Liu[c], Mei-Chu Lo[d], Franck Madoux[c], Narbe Mardirossian[c], Jodi Moriguchi[c], Rachel Ngo[d], Chi-Chi Peng[d], Liping Pettus[c], Nuria Tamayo[c], Paul Wang[c], Rajiv Kapoor[e], Brian Belmontes[c], Sean Caenepeel[c], Paul Hughes[c], Siyuan Liu[c], Katherine K. Slemmons[c], Yajing Yang[c], Fang Xie[d], Sudipa Ghimire-Rijal[c], Susmith Mukund[c], and Sanne Glad[a]

a) Amgen Research, Amgen Inc, Rønnegade 8, DK-2100 Copenhagen, Denmark.
b) Amgen Research, Amgen Inc, Fruebjergvej 3, DK-2100 Copenhagen, Denmark.
c) Amgen Research, Amgen Inc, One Amgen Center Drive, Thousand Oaks, CA 91320, USA.
d) Amgen Research, Amgen Inc, 750 Gateway Blvd, South San Francisco, CA 94080, USA.
e) Amgen Research, Syngene-Amgen Research & Development Center, Biocon Park, Bangalore, 560099, India.
†) Passed away February 22nd, 2023.
*) isarvary@amgen.com

Table of contents

Production of DEL91	S3
Solid phase synthesis of **5** (**5-**solid)	S6
Solution phase synthesis of **5** (**5-**solution)	S8
Molecular modeling of protein-ligand complexes	S10
Protein expression and purification	S12
Data collection and refinement statistics	S14
SDMA levels after repeated dosing	S15
Eurofins (Cerep) screening	S16
Eurofins DiscoverX KINOMEscan™	S18
cLogP of final compounds	S19
UPLC-UV compound **5-solid**	S20
UPLC-UV compound **5-solution** & **6**	S21
UPLC-UV compound **AM-9959** & **7**	S22
UPLC-UV compound **8** & **9**	S23
UPLC-UV compound **10** & **11**	S24
UPLC-UV compound **12** & **AM-9934**	S25
UPLC-UV compound **13** & **14**	S26
UPLC-UV compound **15** & **16**	S27
UPLC-UV compound **17** & **18**	S28
UPLC-UV compound **19** & **20**	S29
UPLC-UV compound **AM-9747** & **21**	S30
^{1}H NMR of **AM-9934**	S31
^{1}HNMR of **AM-9747**	S32
^{13}C NMR of **AM-9747**	S34
COSY of **AM-9747**	S35
NOESY of **AM-9747**	S36
References	S37

Production of DEL91.

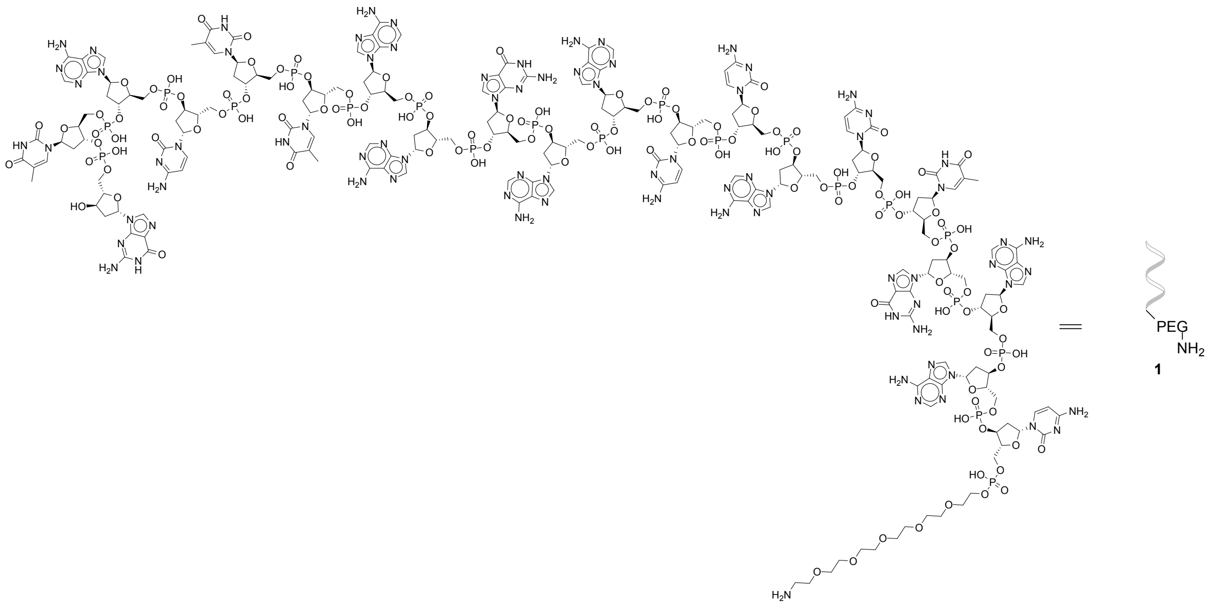

Figure S1. The structure of the head piece **1**.

All on-DNA reactions were heated using PCR thermocyclers.

Size exclusion chromatography (**SEC**) - DNA-linked compounds were purified on pre-washed Bio-Gel P6 columns and eluted with water 1000 G for 4 mins, whereafter the filtrate was collected.

Precipitation - A 5M NaCl solution was added to the DNA-linked compounds, followed by EtOH and the mixture was then cooled to -80 °C for 15 min. Thereafter, the sample was centrifuged at 4 °C for 30 min at 9400 G, the supernatant was removed, and the pellet was washed with cold EtOH (70%) and again centrifuged at 4 °C for 30 min at 9400 G. After removal of the supernatant, the DNA-compound was dried at 37 °C.

Ligation - DNA codon (0.9 nmol) pairs were pre-annealed 80 °C for 2 minutes and then at room temperature for 15 minutes prior to enzymatic ligation. To the DNA-linked compounds (1.4 nmol) was added pre-annealed tags (0.9 nmol), 4 µL Ligase buffer and 1 µL T4 DNA ligase. The mixture was agitated at room temperature for 15 min, then at 37 °C for 15 min, and then again at room temperature overnight followed by subsequent inactivation of the ligase at 80 °C for 10 min.

<u>DEL- 91 production</u>

DNA1-Headpiece-BB1(2)

Attachment to the DNA-headpiece.

2 nmol of the headpiece **1** was added to 768 different wells in 5 μL 200 mM sodium phosphate buffer (pH 8.0). Then the corresponding **BB1** (100 mM in DMSO, 4 μL, 400 nmol) and DMT-MM (360 mM in H_2O, 1 μL, 360 nmol) was added and the mixture was incubated overnight at room temperature. Thereafter, H_2O (40 μL) was added to the reaction mixture and the sample was purified by SEC, subsequently ligated, and pooled to yield **2**.

DNA1-DNA2-Headpiece-BB1-BB2 (3).

Fmoc deprotection

A solution of 10 % piperidine in water (200 μL) was added to the pooled DNA-BB1 bound to DEAE Sephadex A-25 resin and the reaction was incubated at RT for 30 min whereafter the resin was drained. Another portion of 10 % piperidine in water (200 μL) was added to DNA bound on DEAE and the reaction was agitated at RT for 30 min whereafter the resin was drained and washed (2xH_2O, 3xDMF, 5xH_2O). Thereafter, NaCl (1.5 M) was added and agitated at 60 °C for 1 h and the resin was filtered and again treated with another portion of NaCl solution (1.5 M) at 60 °C for 1 h and filtered. Ethanol was added to the combined NaCl filtrate to yield the Fmoc deprotected **2** as the precipitate.

The Fmoc deprotected **2** was split into 262 wells as and subjected to ligation and purified by SEC and split into 262 wells.

Amide Formation

The material of 176 wells containing **2** was subjected to ligation and purified by SEC. Therafter, the amide coupling was performed with the corresponding 176 **BB2** acids (100 mM in DMSO, 4 μL, 400 nmol), as outlined with **BB1**, and then subjected to SEC purification.

S_NAr Substitution

The 63 **BB2** S_NAr electrophiles (400 mM in DMSO, 6 μL, 2400 nmol) were added to 63 wells containing **2** (10 nmol) in sodium phosphate buffer (12 μL, 1.2 M, pH 8.0) and the reaction agitated for 30 minutes at room temperature followed by 1h at 60 °C, and 75 °C overnight. Thereafter, H_2O (40 μL) was added to the reaction mixtures were purified by SEC.

Sulfonylation

The 23 **BB2** sulfonyl chlorides (100 mM in THF, 2 μL, 200 nmol) were added to 23 wells**2** (10 nmol) in sodium borate buffer (8 μL 100 mM pH 9.0) and agitated overnight at RT. Thereafter, H_2O (40 μL) was added to the reactions and purified by SEC.

The SEC purified compounds from the amidation-, S_NAr- and sulfonylation-reactions above were pooled and precipitated to yield **3**.

DNA3-DNA2-DNA1-Headpiece-BB1-BB2-BB3 (4).

The 480 **BB3** amines (400 mM in DMSO, 6 µL, 2400 nmol) were added to **3** (10 nmol) in sodium phosphate buffer (12 µL, 1.2 M, pH 8.0) and the reaction mixtures were agitated overnight at 80 °C. Thereafter, H_2O (40 µL) was added to the reaction mixture, purified by SEC and subjected to ligation. Thereafter, the compounds were pooled, precipitated, and purified by standard polyacrylamide gel: 6% polyacrylamide gel was casted and pre-ran for 1 hour at 60 W, 20 mg of the library was dissolved in 300 µL 2x loading buffer (98 mL formamide, 20 mL 0.5 M EDTA-solution, pH=8, 250 µL 10% bromophenol blue, 250 µL 10% xylene cyanol), and added to the gel which was afterwards run for at 3000V, 300mA, 60W for 1.5 h yielding **4**.

Primer-DNA3-DNA2-DNA1-Headpiece-BB1-BB2-BB3 (DEL91).

Extension Primer (1000 pmol) in a solution containing isothermal amplification buffer (10 µL), $MgSO_4$ (2 µL 100 mM), dNTP mix (7 µL 10 mM), *Bst* 2.0 WarmStart polymerase (9 µL) was added to **4** (200 pmol) in H_2O (22 µL) and the ligation was agitated at 65 °C for 1 h and finally purified by SEC to produce **DEL91**.

Scheme S1. Solid phase synthesis of 5.[a]

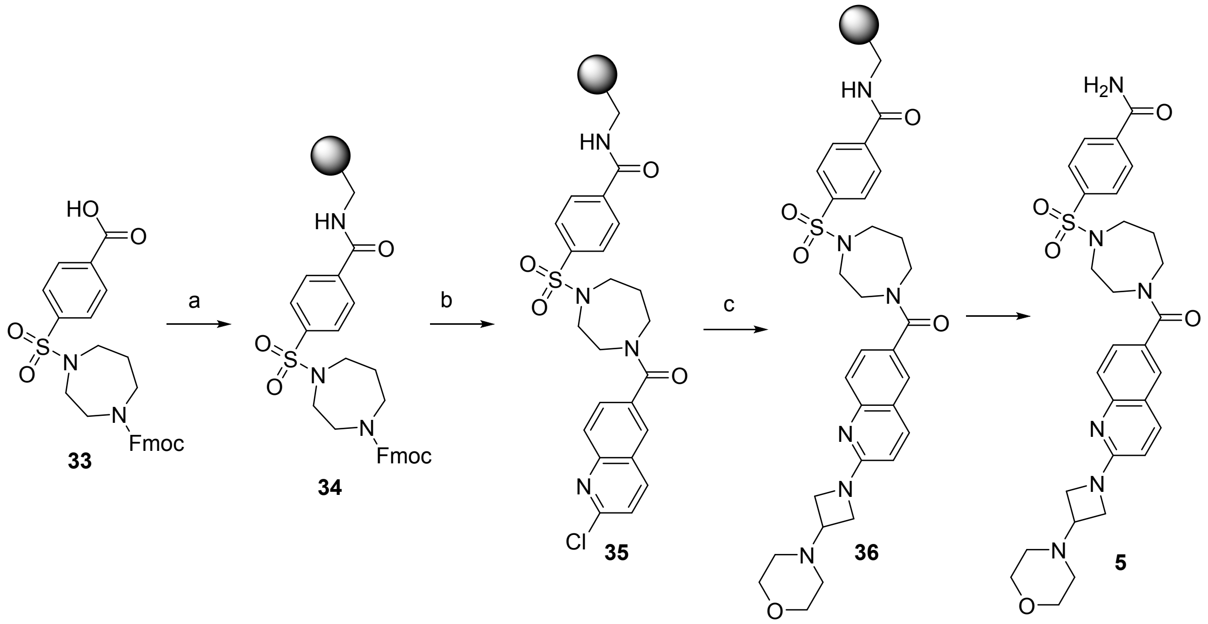

[a] Reagents and conditions: (a) EDC·HCl, HOAt, DIPEA, Rink-amide resin, DMF, 20% piperidine; (b) 2-chloroquinoline-6-carboxylic acid, DMTMM·BF4, DIPEA, DMF, 20% piperidine; (c) 4-(azetidin-3-yl)morpholine hydrochloride, DIPEA, DMF, 80 °C; (d) TFA, DCM, H_2O, rt.

Solid-Phase Synthesis of 4-((4-(2-(3-morpholinoazetidin-1-yl)quinoline-6-carbonyl)-1,4-diazepan-1-yl)sulfonyl)benzamide (5).

Coupling to Resin (**34**)

A solution of 20% Piperidine in DMF was added to Rink amide resin (1.2 µmol) for 2 min, the resin was drained and treated again with a solution of 20% piperidine in DMF for 2 min. Thereafter, the resin was drained and washed (3xDCM, 3xMeOH, 3xDMF, 3xDCM). After that, a solution of 4-((4-(((9H-fluoren-9-yl)methoxy)carbonyl)-1,4-diazepan-1-yl)sulfonyl)benzoic acid (2 equiv), 1-hydroxy-7-azabenzatriazole (2 equiv), and DIPEA (4 equiv) in DMSO:DMF (3:1) was added to the resin, followed by a solution of N, N'-diisopropylcarbodiimide (2 equiv) in DMF, and the reaction was shaken at RT overnight. The reaction solution was drained, and the resin was washed (3xDCM, 3xDMF, 3xDCM). Thereafter a solution of acetic anhydride (10 equiv) and pyridine (20 equiv) in DCM was added the added and shaken at RT for 1h. The reaction solution was drained, and the resin was washed (3xDCM, 3xDMF, 3xDCM).

Amide coupling (**35**)

A solution of 20% Piperidine in DMF 100 was added to the resin for 2 min, the resin was drained and treated again with a solution of 20% piperidine in DMF 100 for 2 min. Thereafter the resin was drained and washed (3xDCM, 3xMeOH, 3xDMF, 3xDCM). After that, a solution of 2-chloroquinoline-6-carboxylic acid (2 equiv) and DIPEA (4 equiv) in DMSO:DMF (2:1), followed by a solution of 4-(4,6-dimethoxy-1,3,5-triazin-2-yl)-4-methyl-morpholinium (DMTMM) (2 equiv) in DMF was added to the resin and the reaction was shaken at RT overnight. The reaction solution was drained, and the resin was washed (3xDCM, 3xMeOH, 3xDMF, 3xDCM).

S_NAr coupling (**36**)

The resin was swelled in DMSO, drained, and then a solution of 1-(azetidin-3-yl)piperidine·2HCl (2.4 equiv) in DMSO, followed by neat DIPEA (6 equiv) and some DMSO, were added to the resin and the reaction was shaken at 80 °C for 48 h. Thereafter, the reaction solution was drained, and the resin was washed (3xDCM, 3xDMF, 3xDCM).

Cleavage from Resin (**5**)

Neat TFA (containing 2.5% H_2O) was added to the resin and the reaction was drained, neat TFA (containing 2.5% H_2O) was added again and thereafter the reaction was shaken at RT for 30 min. The reaction was drained, and the resin was washed (2xMeCN). The combined filtrates were concentrated under reduced pressure and the residue was purified by UPLC to yield **5** (15% yield). Analysis revealed 2 compounds, as shown in the UPLC analysis on page S20.

Major product **5** (88% according to UV @ 254 nm), *m/z* (ESI): found 579.2425, calc 579.2384 $(M+H)^+$.

Minor product **6** (12% according to UV @ 254 nm), m/z (ESI): found 454.1572, calc 454.1544 $(M+H)^+$.

Scheme S2. Solution phase synthesis of 5.[a]

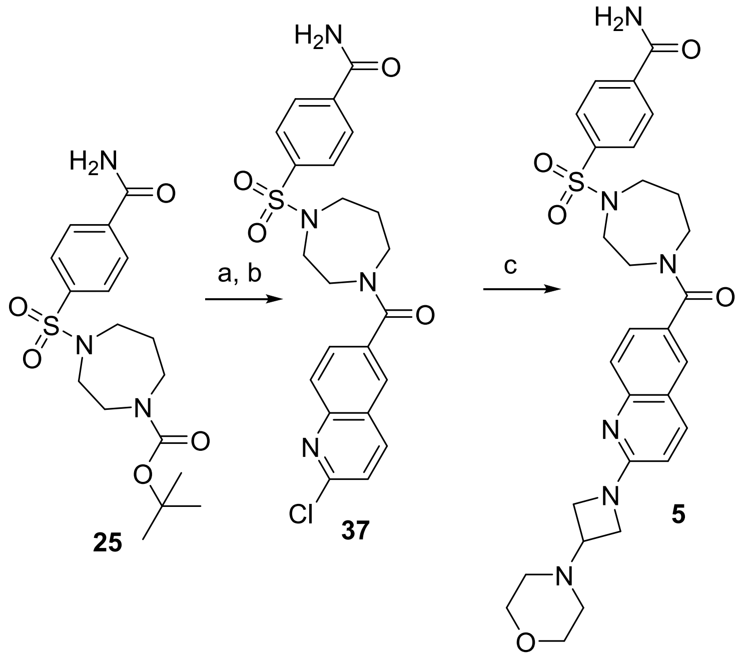

[a] Reagents and conditions: a) TFA, DCM; (b) 2-chloroquinoline-6-carboxylic acid, DMTMM·, DIPEA, DMF; (c) 4-(azetidin-3-yl)morpholine, DIPEA, DMSO, 80 °C.

4-((4-(2-(3-morpholinoazetidin-1-yl)quinoline-6-carbonyl)-1,4-diazepan-1-yl)sulfonyl)benzamide (**5**).

4-((4-(2-chloroquinoline-6-carbonyl)-1,4-diazepan-1-yl)sulfonyl)benzamide (**37**).

TFA (1 mL, 12.98 mmol) was added to a solution of tert-butyl 4-((4-carbamoylphenyl)sulfonyl)-1,4-diazepane-1-carboxylate (107 mg, 0.279 mmol) and DCM (2 mL) after stirring for 10 min the reaction mixture was concentrated under reduced pressure and the crude was used directly in the following step. The crude, 2-chloroquinoline-6-carboxylic acid (57.9 mg, 0.279 mmol), DMTMM· (92 mg, 0.279 mmol) and DMF (3 mL) were mixed and then DIPEA (144 mg, 0.195 mL, 1.116 mmol) was added and the reaction mixture stirred for 16 hours at RT. The mixture was diluted with EtOAc (75 mL) and washed with aq LiCL (5% 2x10 mL), aq $KHSO_4$ (10 %,2x15 mL), sat aq $NaHCO_3$ (2x15 mL), and brine (15 mL). The organic phase was dried (Na_2SO_4), filtered and concentrated under reduced pressure. The residue was purified by Flash CC (SiO_2 HC-D, eluting with 0 % to 100 % EtOAc in heptane, followed by 0 % to 20 % MeOH in EtOAc) to yield **37** (49 mg, 0.104 mmol, 37% yield).

^{1}H NMR (400 MHz, DMSO-d_6) δ ppm 8.46 - 8.52 (m, 1 H), 8.15 - 8.25 (m, 1 H), 7.75 - 8.13 (m, 7 H), 7.63 - 7.71 (m, 2 H), 3.75 - 3.84 (m, 1 H), 3.67 - 3.74 (m, 1 H), 3.44 - 3.56 (m, 2

H), 3.34 - 3.40 (m, 2 H), 3.33 - 3.34 (m, 1 H), 3.32 - 3.33 (m, 1 H), 1.85 - 1.94 (m, 1 H), 1.56 - 1.70 (m, 1 H)

m/z (ESI): $(M+H)^+$ = 472.9.

Compound **37** (45 mg, 95 µmol) and 4-(azetidin-3-yl)morpholine dihydrochloride (82 mg, 0.381 mmol) were added to a solution of DIPEA (0.13 mL, 0.76 mmol) in DMSO (1 mL) and shaken at 80 °C overnight. The reaction mixture was concentrated under (high vacuum). The residue was purified by Flash CC(Amino-D, eluting with 0 % to 100 % EtOAc in heptane, followed by 0 % to 20 % MeOH in EtOAc) and thereafter Flash CC (SiO_2, eluting with 0 % to 100 % MeCN in H_2O, using 0.1 % formic acid as additive) to yield 4-((4-(2-(3-morpholinoazetidin-1-yl)quinoline-6-carbonyl)-1,4-diazepan-1-yl)sulfonyl)benzamide (36 mg, 58 µmol, 61 % yield) as the formic acid salt.

^{1}H NMR (400 MHz, DMSO-d6) δ ppm 8.16 (s, 1 H), 8.06 (d, J=8.5 Hz, 2 H), 8.03 (d, J=8.8 Hz, 1 H), 7.88 (s, 1 H), 7.86 (s, 1 H), 7.69 (s, 1 H), 7.54 (d, J=8.6 Hz, 1 H), 7.44 (d, J=8.6 Hz, 2 H), 6.76 (d, J=8.9 Hz, 1 H), 4.15 (dd, J=8.9, 7.2 Hz, 2 H), 3.95 (dd, J=8.9, 5.1 Hz, 2 H), 3.48 - 3.74 (m, 8 H), 3.20 - 3.48 (m, 6 H), 2.54 (s, 1 H), 2.36 - 2.44 (m, 4 H), 1.65 - 1.88 (m, 2 H). ESI-HRMS: calcd for $C_{29}H_{35}N_6O_5S$: m/z: $[M + H]^+$ = 579.2384; found $[M + H]^+$ = 579.2400.

Molecular modeling of protein-ligand complexes

The crystallographic coordinates of PRMT5:MEP50 in complex with MTA and H4 peptide (PDB code: 5FA5)[1] were downloaded from the Protein Databank and prepared with the "Protein Preparation Workflow" in Maestro[2]. Hydrogen atoms were added to the protein according to the predicted pKa. The H-bond network of various molecular entities found in the crystal structure was then optimized for interaction and finally the Hydrogen atoms positions were energy minimized. The final protein structure was used to prepare inputs (for example, energy grid maps) for various docking software.

Once inputs for various modeling software were prepared, a specific methodology was adopted to predict the ligand-binding pose of the several molecules under investigation. Initially, the docking software Autodock[3], Glide[4] and Plants[5] were run to produce 100 conformations each and their outputs were combined to represent the accessible conformational space of the ligand in the binding site. To identify possible binding modes the docking predictions were then grouped based on the root mean square deviation (RMSD) between conformations. The Autonomous Hierarchical Agglomerative Cluster Analysis Based Protocol (ACIAP) was used for the task[6] which, by means of the KGS penalty function, identifies the optimal cut in the similarity tree, based on the average spread of clusters at each tree level. The medoid conformation for each cluster was kept as well as the best scoring pose for each software. The procedure was implemented in R software.[7]

Selected ligand conformations were minimized within the fixed binding site by means of Amber software package[8], MMFF94 force field parameters were assigned to the ligand atoms. After 1000 of steepest descent plus 1000 of conjugate gradient steps, the resulting conformations were then re-scored with ChemPLP from Plants, X-CSCORE[9] and Drugscore[10]. The resulting values from these scoring functions were combined in a consensus score (with a range from 0 to 1) based on the ranking of the specific conformation for each of the 3 metrics[11].

Finally, to judge the quality of each predicted complex, various aspects of the modeling were exploited: i) the size of docking pose clusters (the more members the better), the RMSD value of the pose before and after the minimization (the lower the better) and the consensus scoring (the closer to 1 the better).

Production, clustering, selection, minimization, and re-scoring of binding conformations were automatized based on a previous workflow implementation[12]. While data handling and execution of third-party software was done in KNIME Analytics Platform[13], the actual computation was performed on a GNU/Linux system through BASH scripts combining

software command line, file format transformations and handling of variables to generalize the process.

The ionization state of the nitrogen in the quinoline ring system was investigated with a pKa calculation at Quantum Mechanical level in Jaguar (through the Maestro software suite[2]). The predicted pKa (6.86) for quinoline was like the experimental value of the close analogue 2-aminopyridine (6.77[14]), suggesting the possible protonation of this nitrogen at physiological pH. Although we considered both states, protonated and unprotonated, of the compound in the modeling investigations, the charged version gave more reliability in terms of interaction metrics and structural attributes.

To explore the binding site characteristics around the ligand, an energy-grid approach was used.[15] Energy maps are calculated with Autogrid[3] based on the interactions between the protein atoms and multiple atomic probes, and the visualization of low-energy grid points unveils regions for possible interactions. Using a carbon atom as probe, favorable hydrophobic contacts are revealed, as Figure 5 in the article.

Protein expression and purification

Protein for x-ray crystallography was recombinantly expressed in a baculovirus expression system according to literature procedures.[16]

Purification was conducted at 4 °C and the following buffers were used:

Lysis buffer: 50mM HEPES pH7.5, 300mM NaCl, 10% (v/v) Glycerol, 1mM TCEP, 0.1% (v/v) Triton X-100

Ni Wash buffer 1: Lysis buffer + 20mM Imidazole pH 8.0

Ni Gradient buffer A: 50mM HEPES pH7.5, 300mM NaCl, 20mM Imidazole pH 8.0, 1mM TCEP

Ni Gradient buffer B: 50mM HEPES pH7.5, 300mM NaCl, 300mM Imidazole pH 8.0, 1mM TCEP

Dialysis buffer: 50mM Tris pH 7.5, 50mM NaCl, 20% Glycerol, 1mM TCEP

MonoQ buffer A: 20mM Bis-Tris pH 6.3, 10% Glycerol, 1mM TCEP

MonoQ buffer B: 20mM Bis-Tris pH 6.3, 10% Glycerol, 1mM TCEP, 1M NaCl

S200 SEC buffer: 50mM Tris pH7.5, 500mM NaCl, 10% Glycerol, 1mM TCEP

TEV PROTEASE: 101493-60; 3 mg/mL

Cell lysis:

Pellets were lysed in **lysis buffer** with constant stirring for 2h, the lysate was centrifuged using t45i rotor at 235,000g for 60 min, and the supernatant was filtered (4 x folded cheesecloth followed by a 0.2 mm membrane filter). The supernatant was loaded onto a NiExcel column, equilibrated with **Ni Wash buffer 1.**

Affinity purification 1 (NiExcel HP):

The supernatant was added to the column and the column was thereafter washed with 5 column volume (CV) of **Ni Wash buffer 1**, followed by a 15 CV wash with **Ni Gradient buffer A**. The bound complex of PRMT5:MEP50 was eluted using a linear gradient (**Ni Gradient buffer A** to **Ni Gradient buffer B**) over 20 CV and the PRMT5:MEP50 containing fractions were pooled. Conc 0.39 mg/mL

TEV cleavage (overnight dialysis):

TEV protease was added (1:100) to the PRMT5:MEP50 solution from above and then mixed well. The was subjected to dialysis (Por, 12,000 – 14,000 Da) in **Dialysis buffer** with constant stirring overnight and then filtered through a 0.2-micron membrane. Conc 0.37 mg/mL

Anion exchange purification (MonoQ 5/50 GL):

The TEV cleaved PRMT5-MEP50 complex was diluted 2X in **MonoQ buffer A** and the pH was further adjusted to ~ pH 6.5 using 1M Bis-Tris pH 6.3 stock solution. This solution was loaded onto the MonoQ column, and the column was thoroughly washed with **MonoQ buffer A**. The bound protein was then eluted using a shallow 40 CV gradient from 0 – 100% **MonoQ buffer B**. Conc 1.8 mg/mL.

Size Exclusion Chromatography:

The Superdex S200 column was equilibrated with **SEC buffer** on a AKTA pure and the pooled PRMT5-MEP50 material from the anionic exchange purification was added.
The fractions thereof were pooled, concentrated to 13.5 mg/mL, flash frozen, and stored at -80 °C for crystallization experiments.

Co-crystal structure determination for AM-9747

PRMT5:MEP50 protein complex at 13.5 mg/mL was mixed with SFG in 1:1.2 molar excess ratio of SFG in 50mM Tris pH7.5, 500mM NaCl, 10% Glycerol, 1mM TCEP and incubated on ice for 15 min prior to crystallization set-up. Protein crystallization was performed using the sitting drop vapor diffusion technique in 96 well trays at 4 °C using Mosquito. Crystals of the PRMT5-MEP50_SFG complex grew from a1:1 mix of this protein complex in 0.1M Na citrate tribasic dihydrate pH 5.6 buffer containing 2% Tascimate pH 5.0, 16% PEG 3350 as precipitant over a period of 1 - 2 weeks. Prior to data collection, the crystals were soaked in a 1:1 molar ratio mixture of **AM-9747** (inhibitor compounds) and MTA in mother liquor supplemented with 20% glycerol as cryo-protectant for 15 minutes at room temperature. The soaked crystals were harvested, and flash frozen in liquid at 100K for data collection.
All data sets were collected on a Pilatus3 6 M silicon pixel detector at the Advanced Light Source Beamline 5.0.2 at wavelength 1.00000 Å and temperature 100 K. The data were integrated and scaled using HKL2000[17]. The structures were solved by molecular replacement using Phaser[18] from the CCP4 program suite[19] with 6CKC as a search model. The structures were refined using Phenix[20]. The structure of PRMT5:MEP50 in complex with MTA and **AM-9747** is determined at 2.25Å resolution with R-factor of 20.9% and Rfree of 24.5%.

Table S1. Data collection and refinement statistics.

	PRMT5-MEP50-MTA-AM-9747
**Data collection**	
Space group	I222
Cell dimensions	
a, *b*, *c* (Å)	104.081 138.135 179.028
α, β, γ (°)	90, 90, 90
Resolution (Å)	41.42 - 2.25 (2.33 - 2.25)
Rmerge	0.12(-6.89)
$I / \sigma I$	30.3(4.4)
Completeness (%)	100(99.9)
Multiplicity	13.4 (12.9)
$CC_{1/2}$	1(0.964)
**Refinement**	
Resolution (Å)	(2.33 - 2.25)
No. reflections	61308
R_{work} / R_{free}	0.20/0.24
No. atoms	
Protein	7385
Ligand/ion	117
Water	440
B-factors	
Protein	55.59
Ligand/ion	54.74
Water	53.28
R.m.s. deviations	
Bond lengths (Å)	0.004
Bond angles (°)	0.74

*Values in parentheses are for highest-resolution shell.

SDMA levels after repeated dosing.

Figure S2.

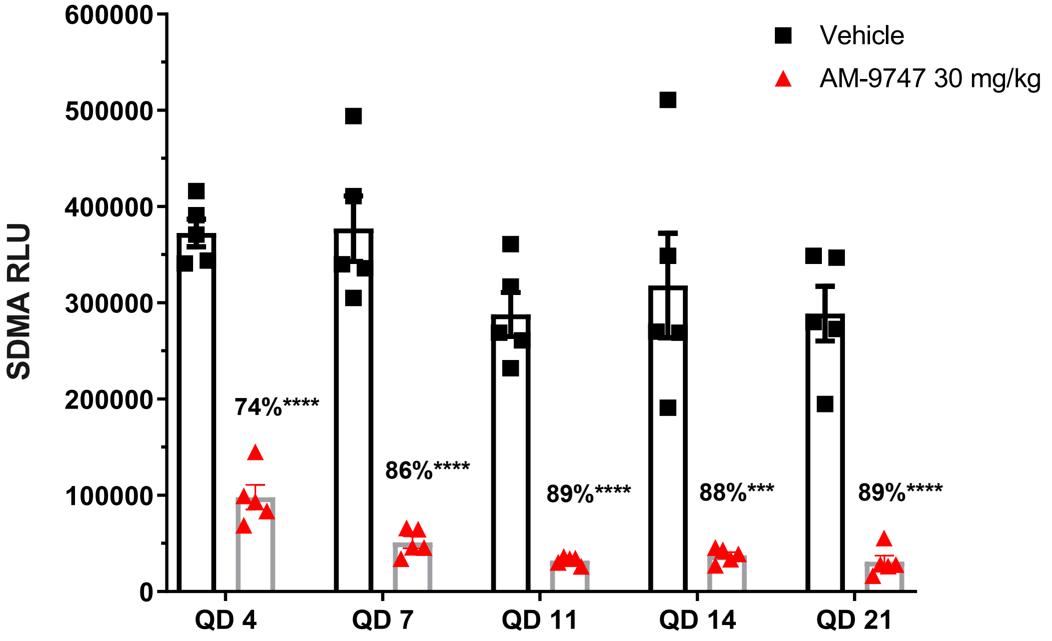

Figure S2. SDMA ELISA analysis of HCT116 MTAP-null tumors. Mice were administered vehicle or **AM-9747** (30 mg/kg) orally (PO) once a day for the duration of the study group. Mice were dosed with either a total of 4, 7, 11, 14, 21 doses and tumors were collected 4 hours post last dose for each group. Percentage of inhibition reported relative to matched vehicle. Data represent mean ± SEM, n = 5 for each group. STATS: One-Way ANOVA w/Dunnett's Comparison to Control p=0.001** , p<0.0001****

Time Course PD Assay: Athymic nude mice were implanted with HCT 116 *MTAP* null cells (2.0E+06 with Matrigel). Animals with established tumors were sorted into groups (n=5) with similar tumor volumes. Animals were administered vehicle or **AM-9747** at 30 mg/kg orally once a day for a total of 4, 7, 11, 14, or 21 total doses. Paired vehicle and **AM-9747** mice were harvest 4 hours post the final dose and processed for PD (SDMA). For SDMA analysis see below process: SDMA Enzyme-linked immunosorbent assay (ELISA). Data were plotted as mean plus SEM using GraphPad Prism 7.05 software. Statistical significance was computed versus matched vehicle by an Ordinary one-way ANOVA at a significance level of 0.05 with Dunnett's multiple comparisons test.

Table S2. Eurofins (Cerep) screening.

Agonist
A2A
A3
α1A
α2A
β1
β2
CB1
CB2
CCK1 (CCKA)
D1
D2L
ETA
H1
H2
kappa (KOP)
M1
M2
M3
δ (DOP)
μ (MOP)
IP (PGI2)
TP (TXA2/PGH2)
5-HT1A
5HT1B
5-HT2A
5-HT2B
V1a
BZD
N neuronal α4β2
GR
AR
CysLT$_1$ (LTD$_4$)

Antagonist
A2A
A3
α1A
α2A
β1
β2
CB1
CB2
CCK1 (CCKA)
D1
D2L
ETA
H1
H2
kappa (KOP)
M1
M2
M3
δ (DOP)
μ (MOP)
IP (PGI2)
TP (TXA2/PGH2)
5-HT1A
5HT1B
5-HT2A
5-HT2B
V1a
NMDA
5-HT3
K_V channel
Ca^{2+} channel (L, dihydropyridine site)
K_{ATP} channel
Na$^+$ channel (site 2)

Other activity
COX1
COX2
PDE3A
PDE4D2
IRK (InsR)
KDR kinase (VEGFR2)
Lck kinase
ROCK1
Acetylcholinesterase
MAO-A
Norepinephrin transporter uptake
Dopamine uptake
5-HT uptake
Potassium Channel hERG
TXA$_2$ synthetase
PDE6
Cathepsin D
ATPase (Na$^+$/K$^+$)

The targets screened at Eurofins (Cerep) for activity of **AM-9747** in concentrations up to 10 μM.

Figure S3. The activity of **AM-9747** upon the μ (MOP) receptor as an agonist.

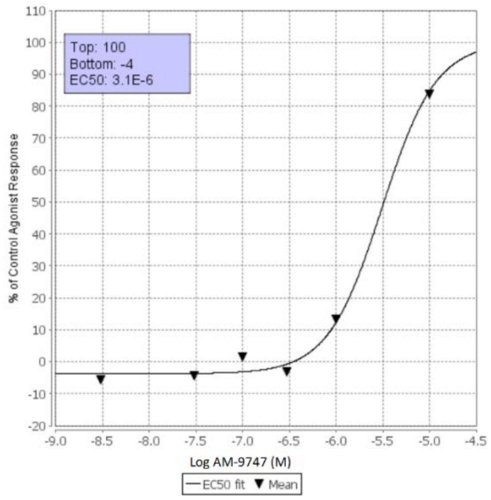

Figure S3. The only significant effect (inhibition or stimulation, ≥50% , at concentrations ≤ 10 μM) of **AM-9747** upon the "Cerep" was upon the μ (MOP) receptor as an agonist, displaying an EC50 of 3.1 μM.

Table S3. The DiscoverX KINOMEscan™.

Gene Symbol	%Ctrl @ 1000nM	Gene Symbol	%Ctrl @ 1000nM
ABL1-nonphosphorylated	80	MARK1	92
ABL1-phosphorylated	87	MEK3	83
AKT1	100	MEK5	84
ALK	59	MET	94
AMPK-alpha1	95	MKNK1	100
AURKA	99	MLK1	95
BMPR1A	93	MST2	100
BRAF	100	MTOR	97
BRK	99	NEK4	97
BTK	92	NEK6	99
CAMK2D	100	NK2	100
CAMK4	100	OSR1	90
CDK2	100	p38-alpha	91
CDK4-cyclinD1	86	p38-gamma	81
CDK8	100	PAK2	95
CHEK1	97	PCTK3	100
CLK4	100	PDGFRB	99
CSNK1D	100	PIK3CA	81
CSNK1G2	96	PIK3CD	64
CSNK2A1	80	PIM1	100
DAPK1	98	PKAC-alpha	100
DDR1	96	PLK1	93
DMPK	97	PLK4	95
DYRK1A	91	PRKCE	91
EGFR	97	PRKD2	94
EPHA2	93	PRKG1	100
EPHB3	100	PRKR	77
ERK2	100	RIPK1	94
ERK4	100	ROCK2	100
ERN1	64	RPS6KA5(Kin.Dom.1-N-terminal)	100
FGFR1	97	RSK1(Kin.Dom.1-N-terminal)	91
FLT3	100	S6K1	96
FYN	67	SRC	100
GAK	100	STK33	88
GRK1	96	SYK	100
GSK3B	94	TAK1	81
HIPK1	78	TAOK2	89
HPK1	99	TBK1	88
IGF1R	96	TGFBR1	87
INSR	94	TGFBR2	100
IRAK4	80	TIE2	82
JAK2(JH1domain-catalytic)	100	TNIK	100
JNK3	92	TRKA	95
KIT	100	TSSK1B	100
LCK	100	TTK	93
LIMK1	78	TYK2(JH1domain-catalytic)	99
LYN	83	VEGFR2	94
MAP3K1	78	WEE1	98
MAP4K4	100	YSK4	94
MAPKAPK2	100	ZAK	88

No significant activity was observed for **AM-9747** upon the 100 kinase screened at DiscoverX KINOMEscan™ for 100 kinases at 1 μM.

Table S4. The cLogP of final compounds

Compd	Structure	CLogP		Compd	Structure	CLogP
5	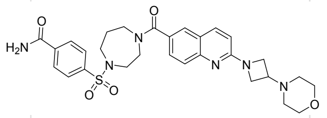	1.44		13	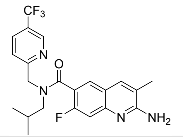	4.32
6	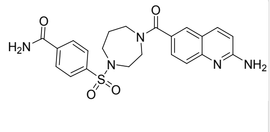	1.00		14	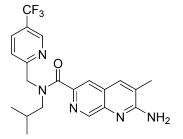	3.31
AM-9959	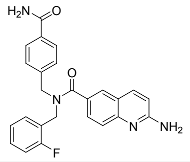	3.13		15	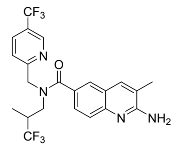	3.77
7	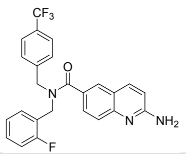	5.00		16	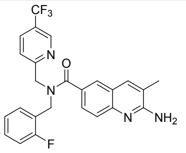	4.60
8	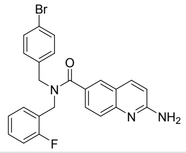	5.48		17	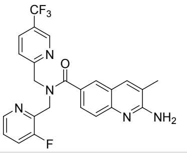	3.19
9	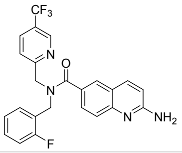	4.15		18	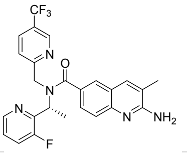	3.49
10	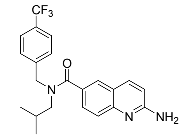	5.05		19	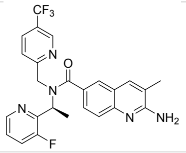	3.49
11	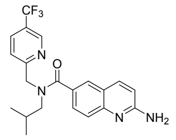	3.70		20	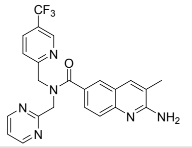	2.00
12	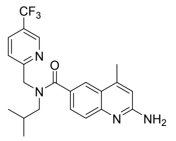	4.19		AM-9747	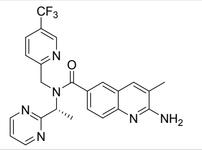	2.31
AM-9934	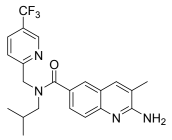	4.14		21	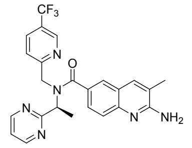	2.31

The cLogP of the tested compounds.

UPLC UV purity @ 254 nm for 5-solid - Solid Phase Synthesis.

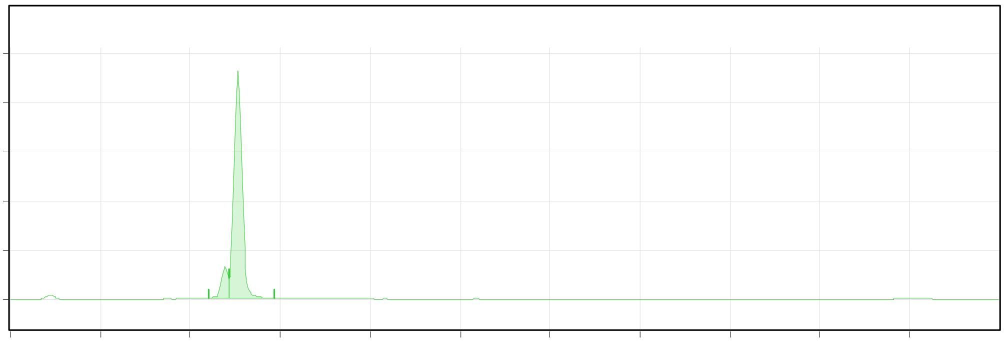

Integration Peak List

Peak	Start	RT	End	Height	Area	Area %
1	1.1	1.192	1.214	326.59	886.05	12.03
2	1.214	1.264	1.467	2338.09	7363	100

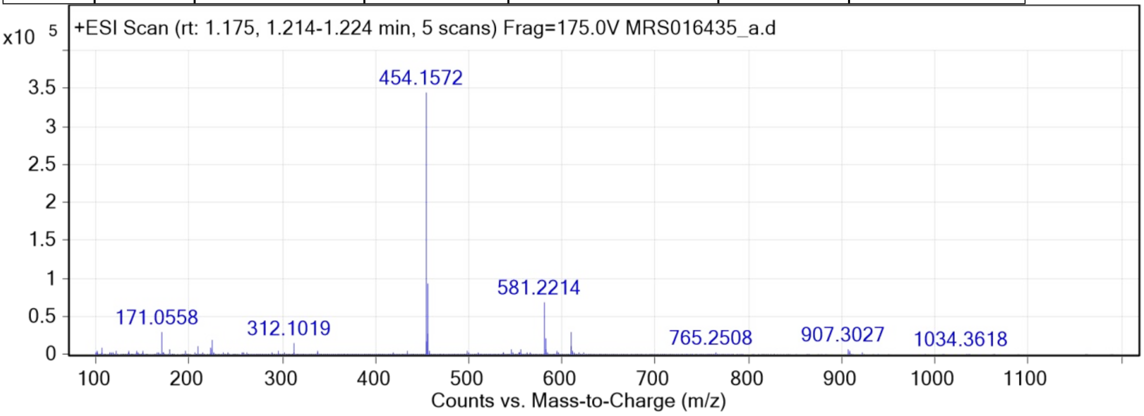

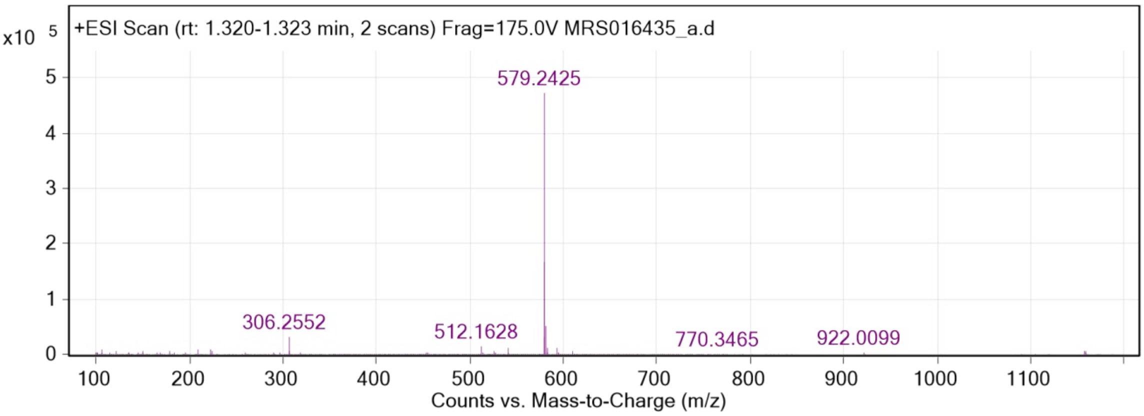

UPLC UV purity @ 254 nm for 5-solution - Solution Phase Synthesis

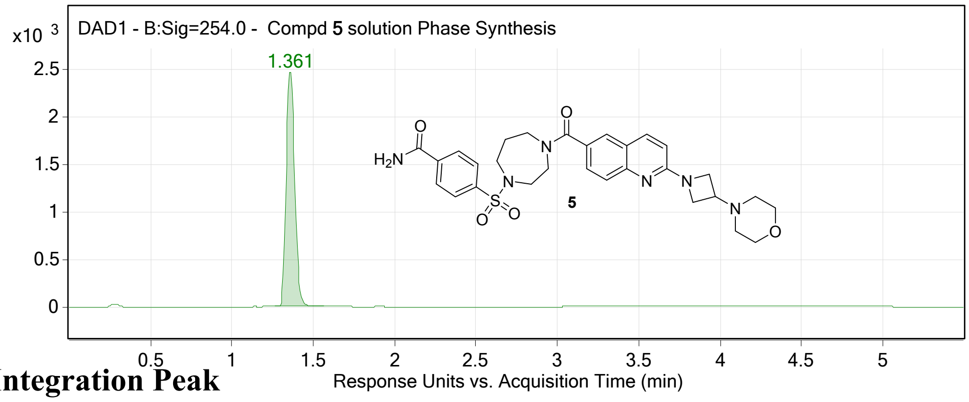

Integration Peak List

Peak	Start	RT	End	Height	Area	Area %
1	1.267	1.361	1.567	2467.76	9159.11	100

UPLC UV purity @ 254 nm for 6.

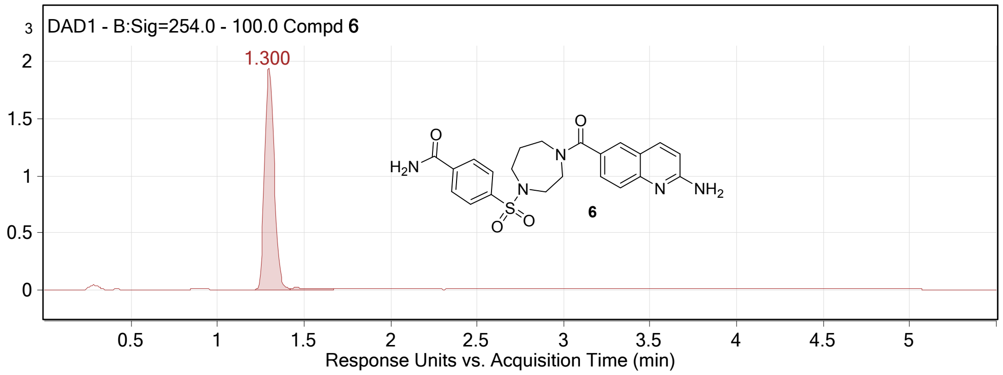

Integration Peak List

Peak	Start	RT	End	Height	Area	Area %
1	1.22	1.3	1.42	1944.87	7562.86	100
2	1.42	1.454	1.667	14.41	95.59	1.26

UPLC UV purity @ 254 nm for AM-9959.

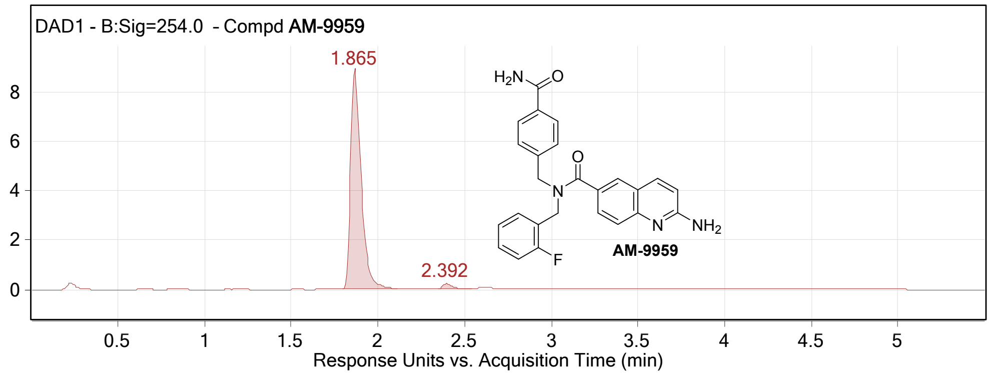

Integration Peak List

Peak	Start	RT	End	Height	Area	Area %
1	1.793	1.865	2.112	896.97	3848.87	100
2	2.348	2.392	2.539	22.03	83.15	2.16

UPLC UV purity @ 254 nm for 7.

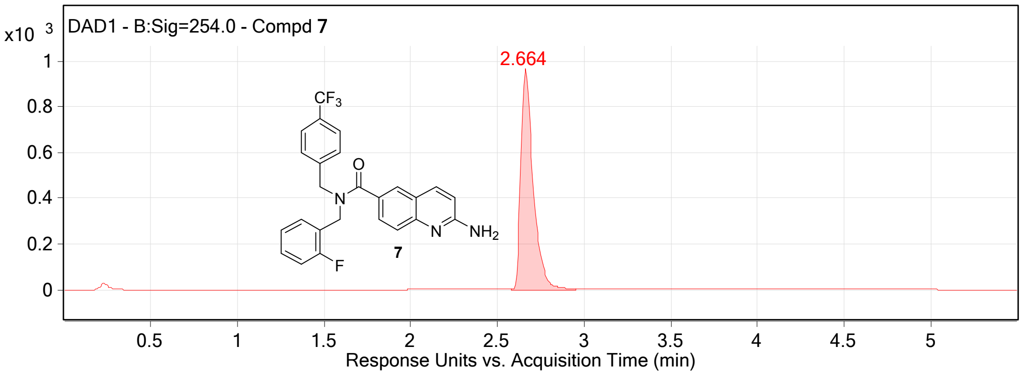

Integration Peak List

Peak	Start	RT	End	Height	Area	Area %
1	2.584	2.664	2.95	967.84	4838.51	100

UPLC UV purity @ 254 nm for 8.

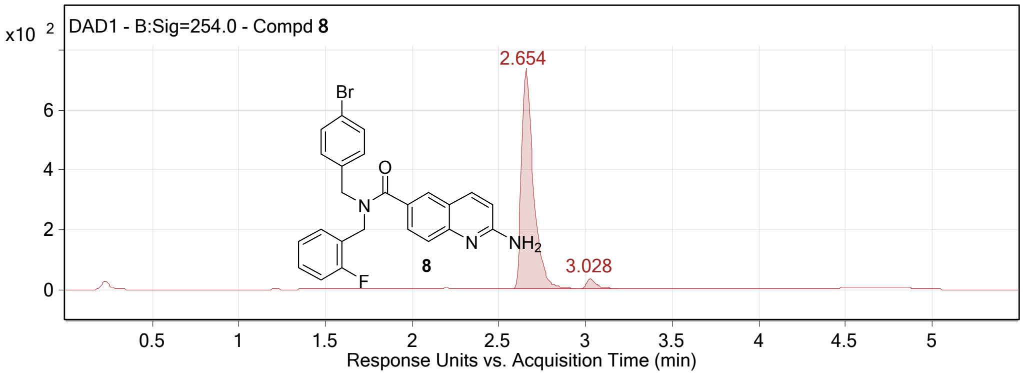

Integration Peak List

Peak	Start	RT	End	Height	Area	Area %
1	2.588	2.654	2.941	737.53	3528.49	100
2	2.974	3.028	3.182	31.24	118.27	3.35

UPLC UV purity @ 254 nm for 9.

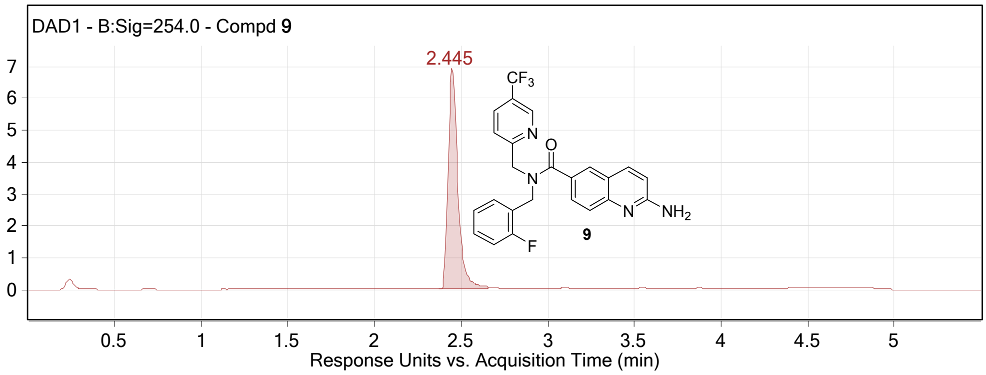

Integration Peak List

Peak	Start	RT	End	Height	Area	Area %
1	2.372	2.445	2.652	692.73	2709.54	100

UPLC UV purity @ 254 nm for 10.

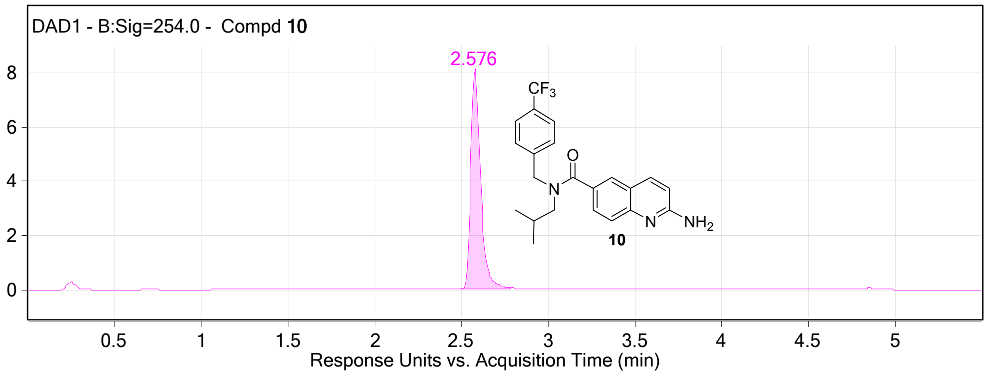

Integration Peak List

Peak	Start	RT	End	Height	Area	Area %
1	2.489	2.576	2.783	814.41	3263.91	100

UPLC UV purity @ 254 nm for 11.

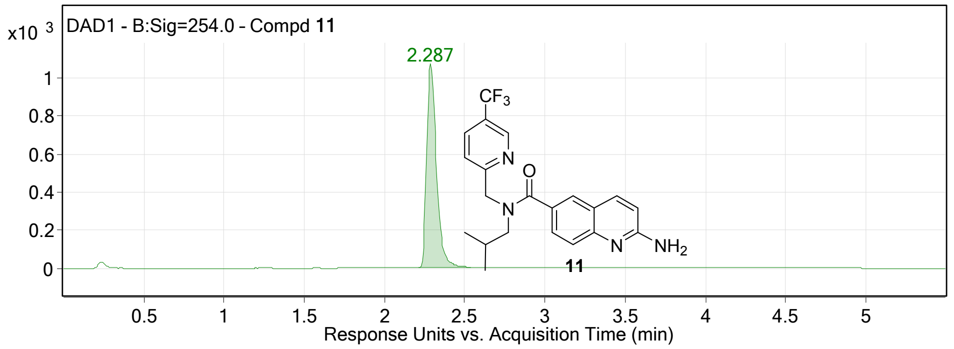

Integration Peak List

Peak	Start	RT	End	Height	Area	Area %
1	2.215	2.287	2.534	1072.76	4581.92	100

UPLC UV purity @ 254 nm for 12.

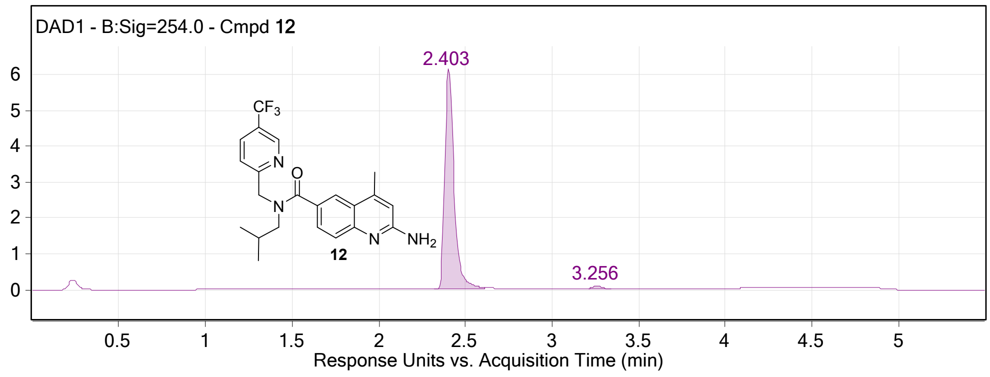

Integration Peak List

Peak	Start	RT	End	Height	Area	Area %
1	2.323	2.403	2.609	613.85	2299.76	100
2	3.209	3.256	3.343	8.02	23.17	1.01

UPLC UV purity @ 254 nm for AM-9934.

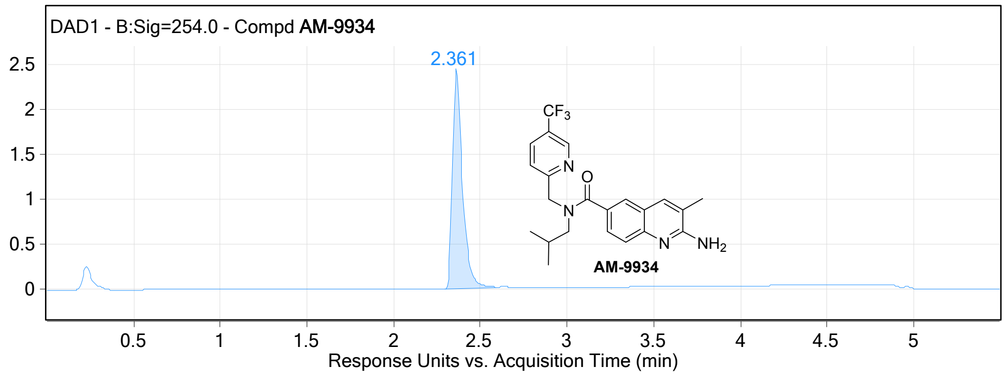

Integration Peak List

Peak	Start	RT	End	Height	Area	Area %
1	2.298	2.361	2.595	243.44	982.05	100

UPLC UV purity @ 254 nm for 13.

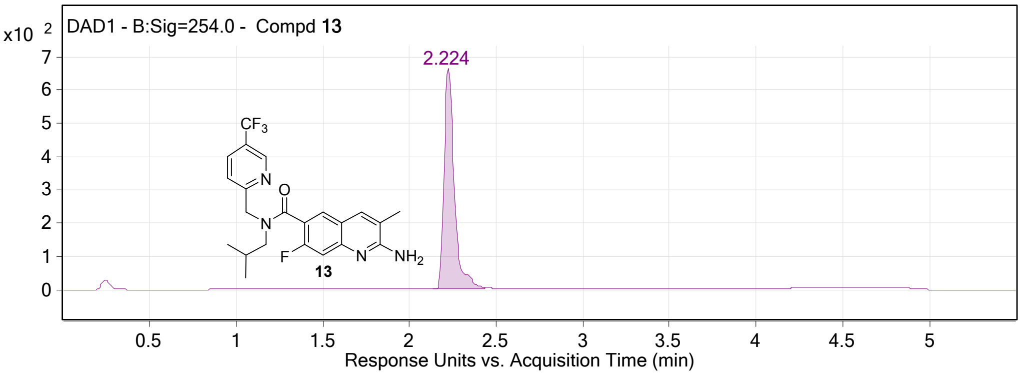

Integration Peak List

Peak	Start	RT	End	Height	Area	Area %
1	2.138	2.224	2.431	663.45	2728.99	100

UPLC UV purity @ 254 nm for 14.

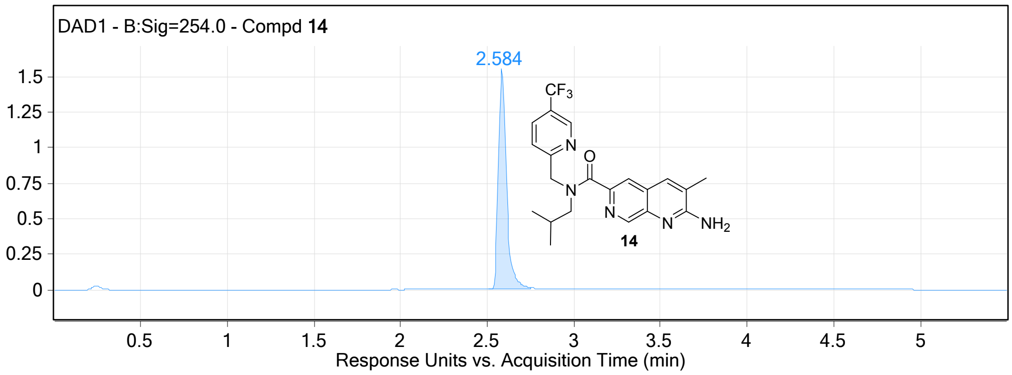

Integration Peak List

Peak	Start	RT	End	Height	Area	Area %
1	2.511	2.584	2.751	1555.78	5169.06	100

UPLC UV purity @ 254 nm for 15.

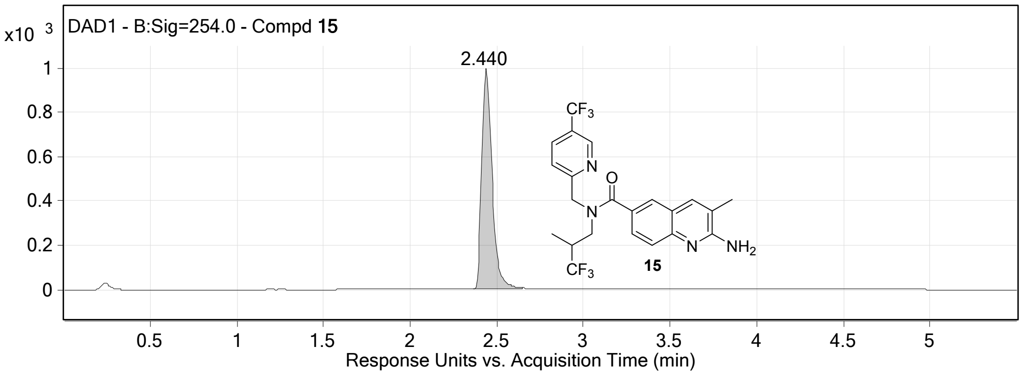

Integration Peak List

Peak	Start	RT	End	Height	Area	Area %
1	2.367	2.44	2.646	996.2	4150.94	100

UPLC UV purity @ 254 nm for 16.

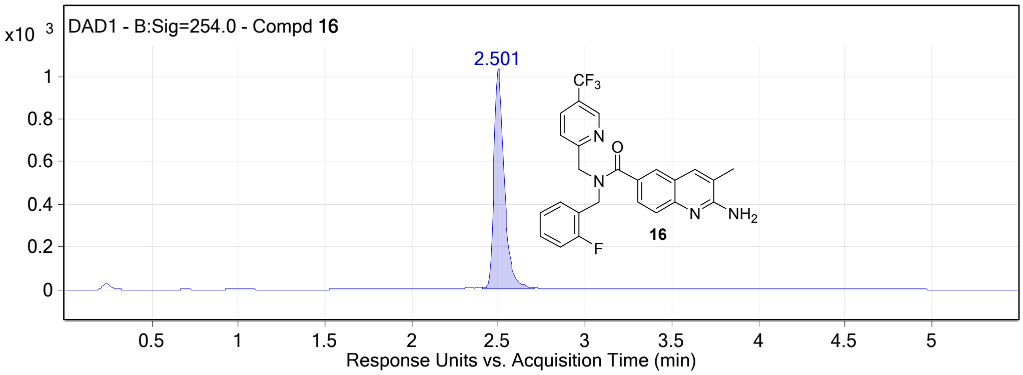

Integration

Peak List

Peak	Start	RT	End	Height	Area	Area %
1	2.408	2.501	2.708	1034.73	4332.86	100

UPLC UV purity @ 254 nm for 17.

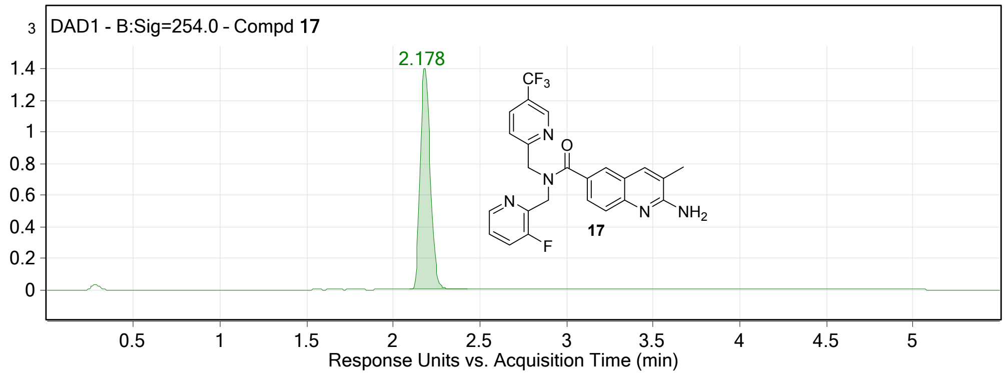

Integration Peak List

Peak	Start	RT	End	Height	Area	Area %
1	2.098	2.178	2.425	1400.15	5708.32	100

UPLC UV purity @ 254 nm for 18.

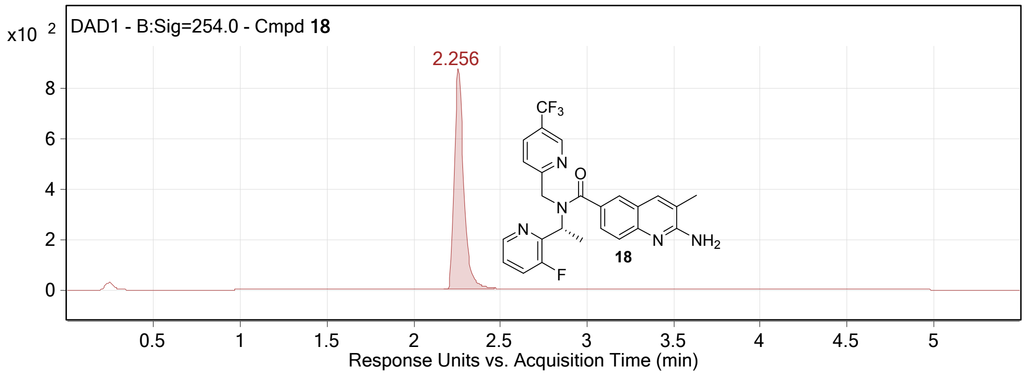

Integration Peak List

Peak	Start	RT	End	Height	Area	Area %
1	2.176	2.256	2.463	877.72	3396.37	100

UPLC UV purity @ 254 nm for 19.

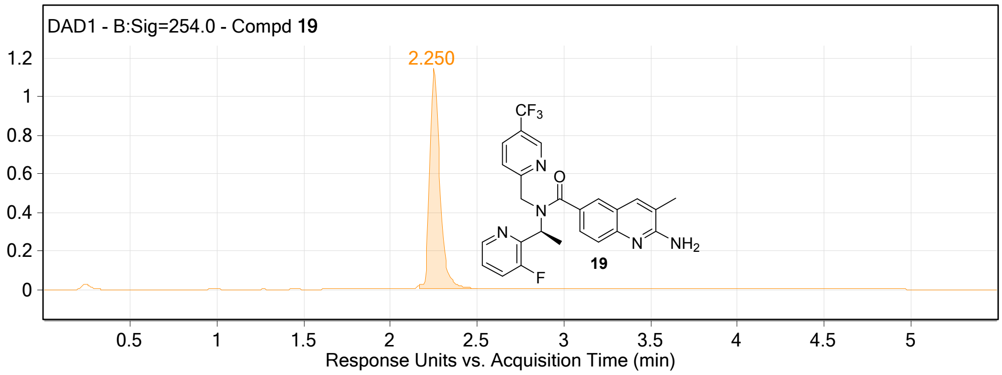

Integration Peak List

Peak	Start	RT	End	Height	Area	Area %
1	2.163	2.25	2.497	1145.72	4752.77	100

UPLC UV purity @ 254 nm for 20

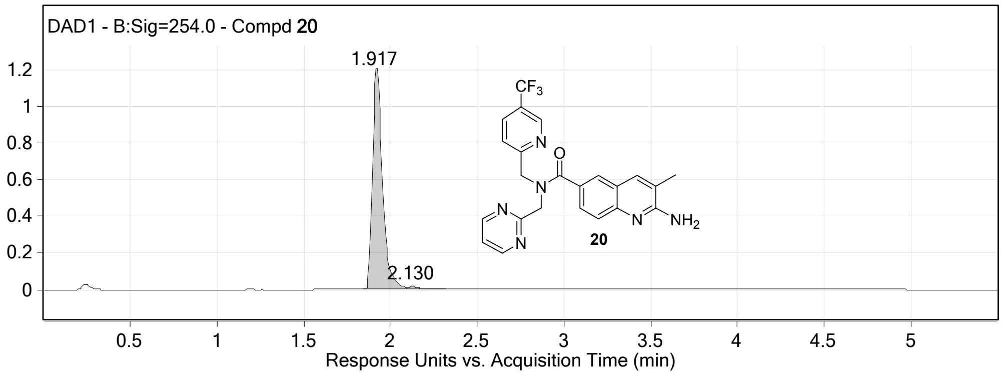

Integration Peak List

Peak	Start	RT	End	Height	Area	Area %
1	1.843	1.917	2.097	1205.05	4894.69	100
2	2.097	2.13	2.317	14.88	64.93	1.33

UPLC UV purity @ 254 nm for AM-9747.

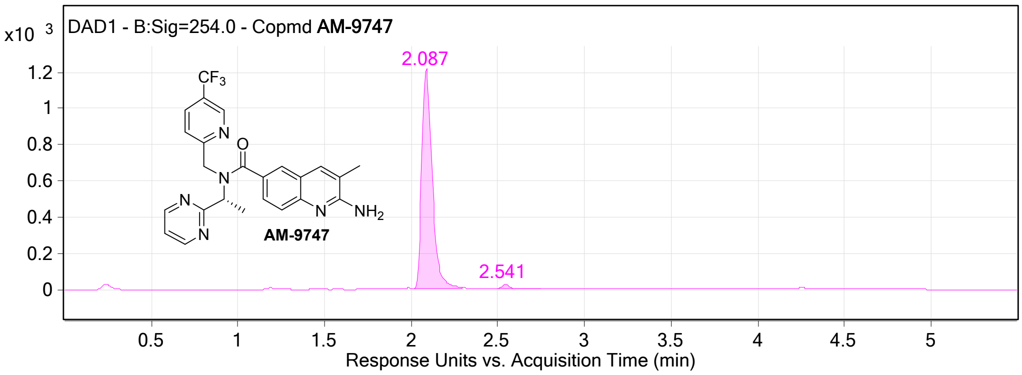

Integration Peak List

Peak	Start	RT	End	Height	Area	Area %
1	2.014	2.087	2.294	1219.21	5109.91	100
2	2.494	2.541	2.748	25.97	94.26	1.84

UPLC UV purity @ 254 nm for 21.

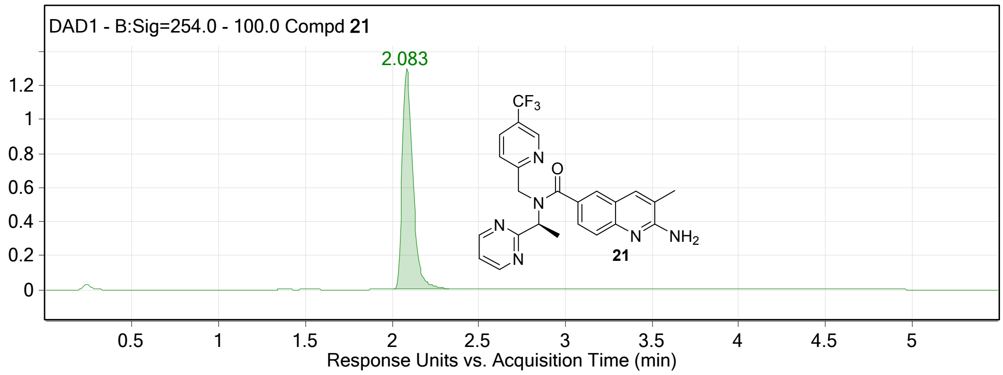

Integration Peak List

Peak	Start	RT	End	Height	Area	Area %
1	2.009	2.083	2.329	1299.94	5543.08	100

^{1}H NMR of AM-9934.

Acquisition Time (sec)	4.1943	*Comment*	Z167430_0029 (CPP1.1 BBO 400S1 BB-H&F-D-05 Z XT)		
Date	05 Jan 2024 13:55:13				
File Name	\\DKCN-VFSX-CF01\ARC_Labdata\Instrument Data\NMR400Mhz_506935\2023\isarvary\N023989-11\1\PDATA\1\1r				
Frequency (MHz)	400.0300	*Nucleus*	1H	*Number of Transients*	64
Owner	SHR-DKCN-BENCHTOP			*Points Count*	65536
Pulse Sequence	zg30	*Solvent*	DMSO-d6	*Spectrum Offset (Hz)*	2466.9038
Sweep Width (Hz)	7812.38	*Temperature (degree C)*	66.999		

ACD version 2017

Data processed on:
01-10-2024 12:18:47

^{1}H NMR (400 MHz, DMSO-d_6, 67°C) δ ppm 8.89 - 8.94 (m, 1 H), 8.14 (dd, *J*=8.2, 1.8 Hz, 1 H), 7.74 (s, 1 H), 7.65 (d, *J*=1.3 Hz, 1 H), 7.50 - 7.59 (m, 1 H), 7.46 (d, *J*=8.6 Hz, 1 H), 7.43 (dd, *J*=8.5, 2.0 Hz, 1 H), 6.23 (s, 2 H), 4.81 (s, 2 H), 3.28 (d, *J*=7.4 Hz, 2 H), 2.22 - 2.24 (m, 3 H), 1.91 - 2.06 (m, 1 H), 0.82 (br s, 6 H)

23 H's / 23 H's (spectrum / structure)

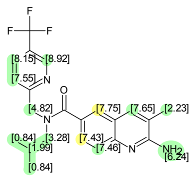

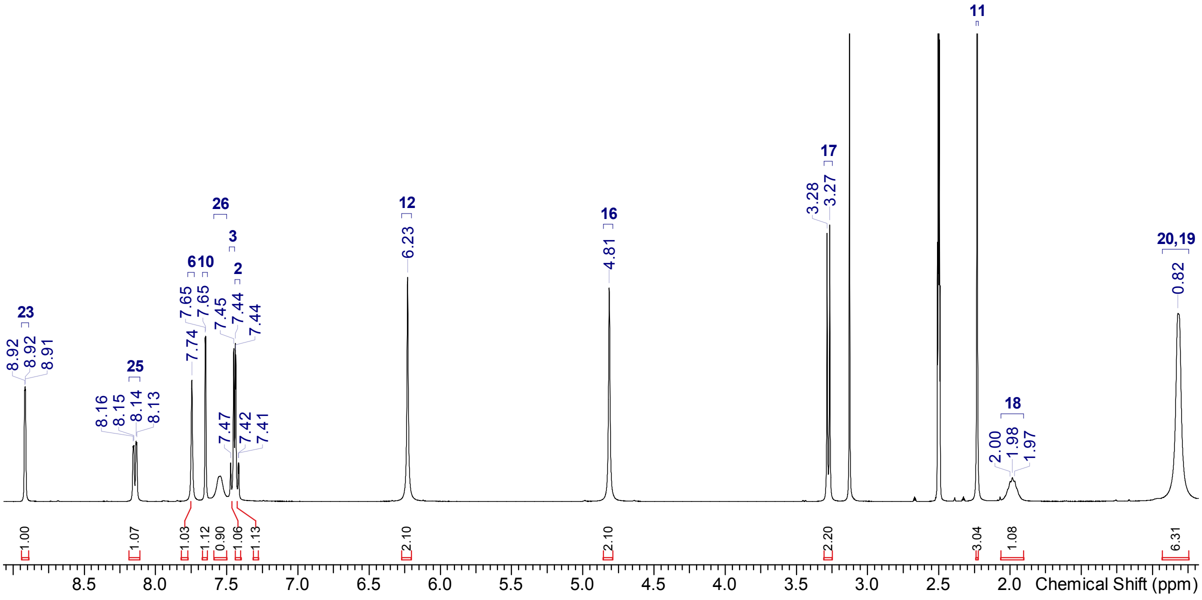

^{1}H NMR of AM-9747 -I

ACD version 2017

Data processed on:
01-12-2024 11:24:18

Acquisition Time (sec)	4.1943	*Comment*	Z167430_0029 (CPP1.1 BBO 400S1 BB-H&F-D-05 Z XT)		
Date	05 Jan 2024 14:48:56				
File Name	\\DKCN-VFSX-CF01\ARC_Labdata\Instrument Data\NMR400Mhz_506935\2023\isarvary\N049735-7\2\PDATA\1\1r				
Frequency (MHz)	400.0300	*Nucleus*	1H	*Number of Transients*	128
Owner	SHR-DKCN-BENCHTOP			*Points Count*	32768
Pulse Sequence	zg30	*Solvent*	DMSO-d6	*Spectrum Offset (Hz)*	2466.9399
Sweep Width (Hz)	7812.26	*Temperature (degree C)*	72.000		

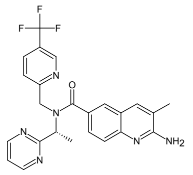

^{1}H NMR (400 MHz, DMSO-d_6, 72°C) δ ppm 8.78 - 8.81 (m, 1 H), 8.76 (d, *J*=4.8 Hz, 2 H), 8.06 (dd, *J*=8.3, 2.1 Hz, 1 H), 7.71 - 7.78 (m, 2 H), 7.52 - 7.59 (m, 2 H), 7.47 (br d, *J*=8.5 Hz, 1 H), 7.36 (t, *J*=4.8 Hz, 1 H), 6.23 (s, 2 H), 5.50 (br q, *J*=7.2 Hz, 1 H), 4.94 (d, *J*=16.9 Hz, 1 H), 4.63 (br d, *J*=16.9 Hz, 1 H), 2.24 (s, 3 H), 1.62 (d, *J*=7.2 Hz, 3 H)

21 H's / 21 H's (spectrum / structure)

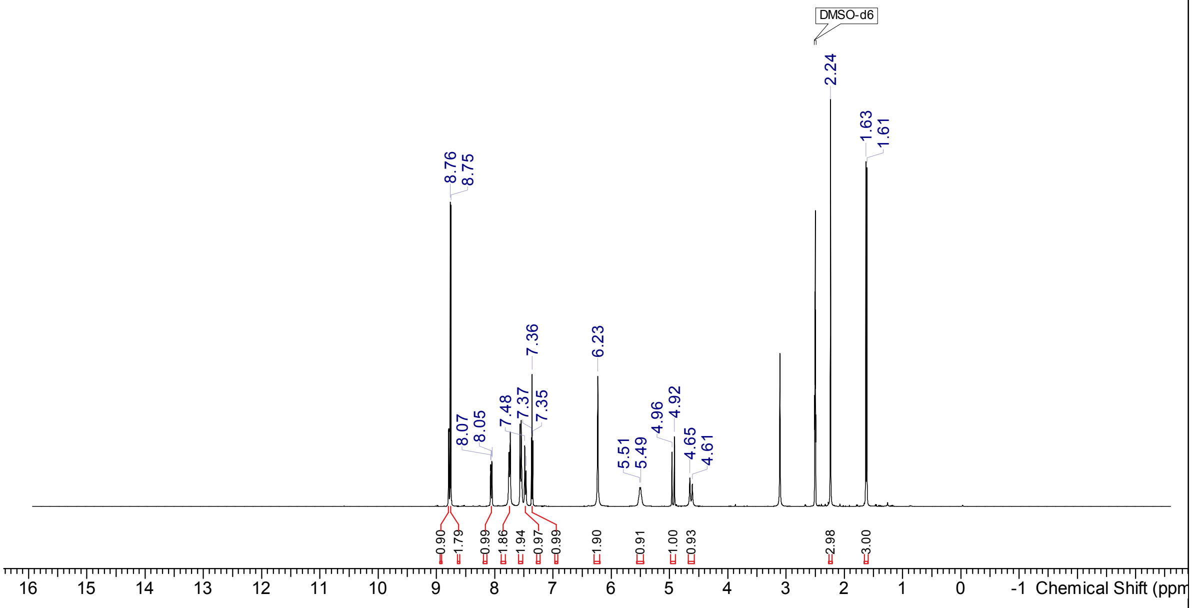

^{1}H NMR of AM-9747-II.

ACD version 2017

Data processed on:
01-12-2024 11:28:10

Acquisition Time (sec)	4.1943	*Comment*	Z167430_0029 (CPP1.1 BBO 400S1 BB-H&F-D-05 Z XT)		
Date	05 Jan 2024 14:48:56				
File Name	\\DKCN-VFSX-CF01\ARC_Labdata\Instrument Data\NMR400Mhz_506935\2023\isarvary\N049735-7\2\PDATA\1\1r				
Frequency (MHz)	400.0300	*Nucleus*	1H	*Number of Transients*	128
Owner	SHR-DKCN-BENCHTOP			*Points Count*	32768
Pulse Sequence	zg30	*Solvent*	DMSO-d6	*Spectrum Offset (Hz)*	2466.9399
Sweep Width (Hz)	7812.26	*Temperature (degree C)*	72.000		

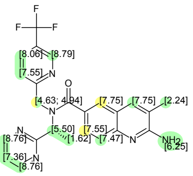

^{1}H NMR (400 MHz, DMSO-d_6, 72°C) δ ppm 8.78 - 8.81 (m, 1 H), 8.76 (d, *J*=4.8 Hz, 2 H), 8.06 (dd, *J*=8.3, 2.1 Hz, 1 H), 7.71 - 7.78 (m, 2 H), 7.52 - 7.59 (m, 2 H), 7.47 (br d, *J*=8.5 Hz, 1 H), 7.36 (t, *J*=4.8 Hz, 1 H), 6.23 (s, 2 H), 5.50 (br q, *J*=7.2 Hz, 1 H), 4.94 (d, *J*=16.9 Hz, 1 H), 4.63 (br d, *J*=16.9 Hz, 1 H), 2.24 (s, 3 H), 1.62 (d, *J*=7.2 Hz, 3 H)

21 H's / 21 H's (spectrum / structure)

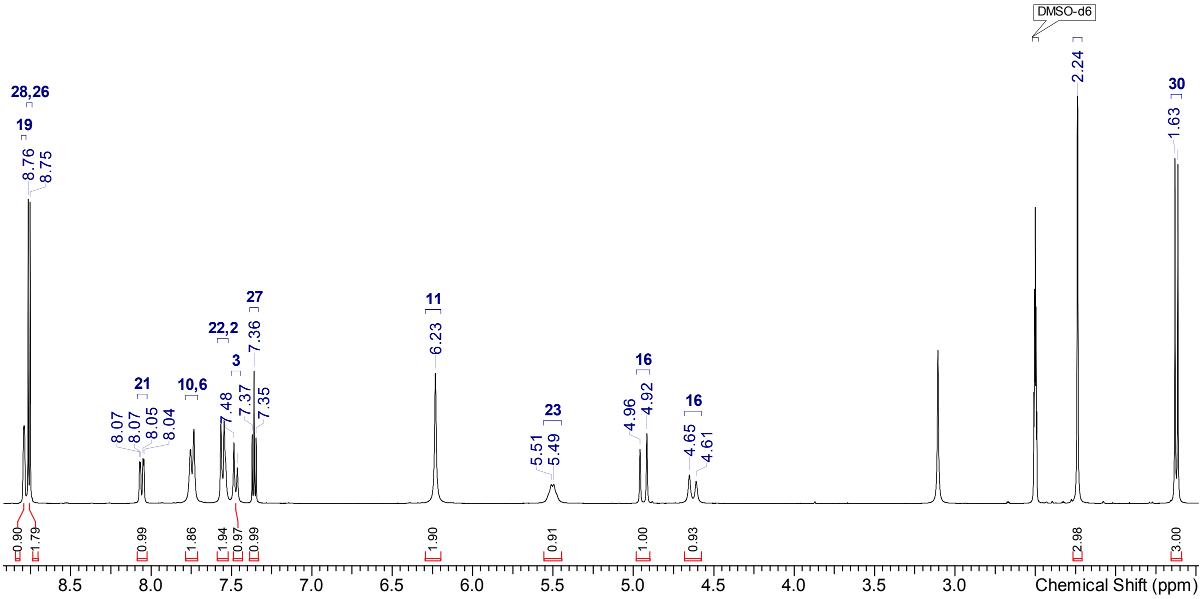

^{13}C NMR of AM-9747.

ACD version 2017

Data processed on:
01-12-2024 11:32:17

Acquisition Time (sec)	1.3763	Comment	Z167430_0029 (CPP1.1 BBO 400S1 BB-H&F-D-05 Z XT)		
Date	05 Jan 2024 21:03:26				
File Name	\\DKCN-VFSX-CF01\ARC_Labdata\Instrument Data\NMR400Mhz_506935\2023\isarvary\N049735-7\3\PDATA\1\1r				
Frequency (MHz)	100.5876	Nucleus	13C	Number of Transients	4096
Owner	SHR-DKCN-BENCHTOP			Points Count	32768
Pulse Sequence	zgpg30	Solvent	DMSO-d6	Spectrum Offset (Hz)	9974.9170
Sweep Width (Hz)	23808.80	Temperature (degree C)	71.999		

^{13}C NMR (101 MHz, DMSO-d_6, 72°C) δ ppm 171.65 (s, 1 C), 168.03 (s, 1 C), 162.93 (s, 1 C), 158.08 (s, 1 C), 156.95 (s, 2 C), 146.90 (s, 1 C), 144.94 (q, *J*=4.1 Hz, 1 C), 135.15 (s, 1 C), 133.25 (q, *J*=3.1 Hz, 1 C), 128.67 (s, 1 C), 126.11 (s, 1 C), 125.02 (s, 1 C), 124.41 (s, 1 C), 122.99 (q, *J*=32.5 Hz, 1 C), 122.39 (s, 1 C), 120.96 (s, 1 C), 120.59 (s, 1 C), 119.54 (s, 1 C), 123.48 (q, *J*=271.8 Hz, 1 C), 58.72 (s, 1 C), 48.24 (s, 1 C), 16.96 (s, 1 C), 16.79 (s, 1 C)

24 C's / 24 C's (spectrum / structure)

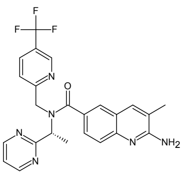

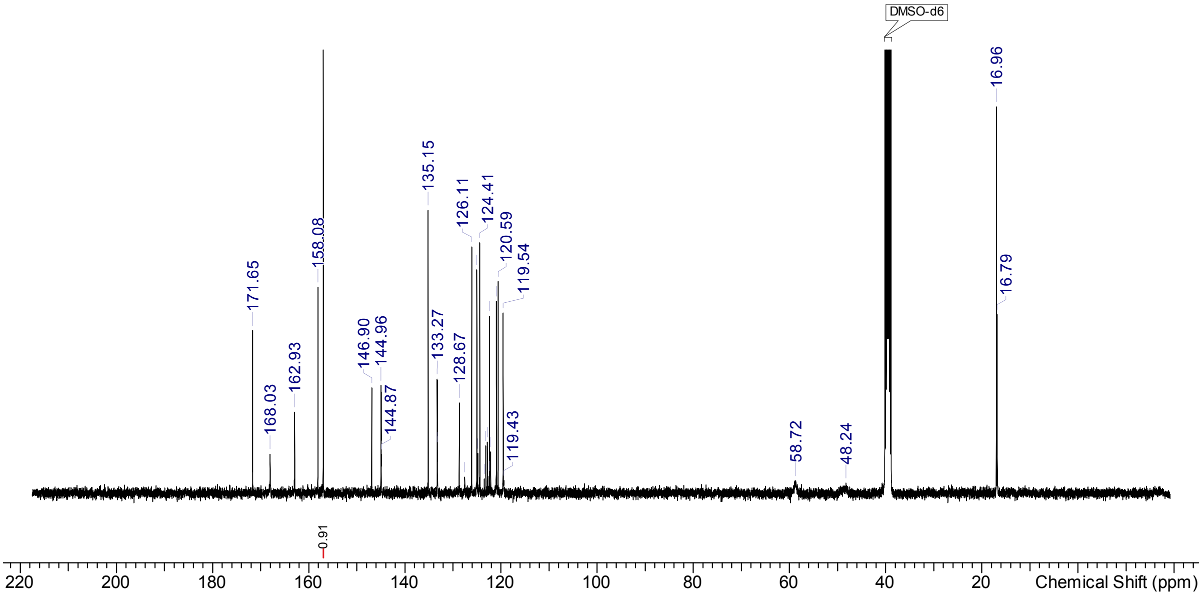

COSY of AM-9747

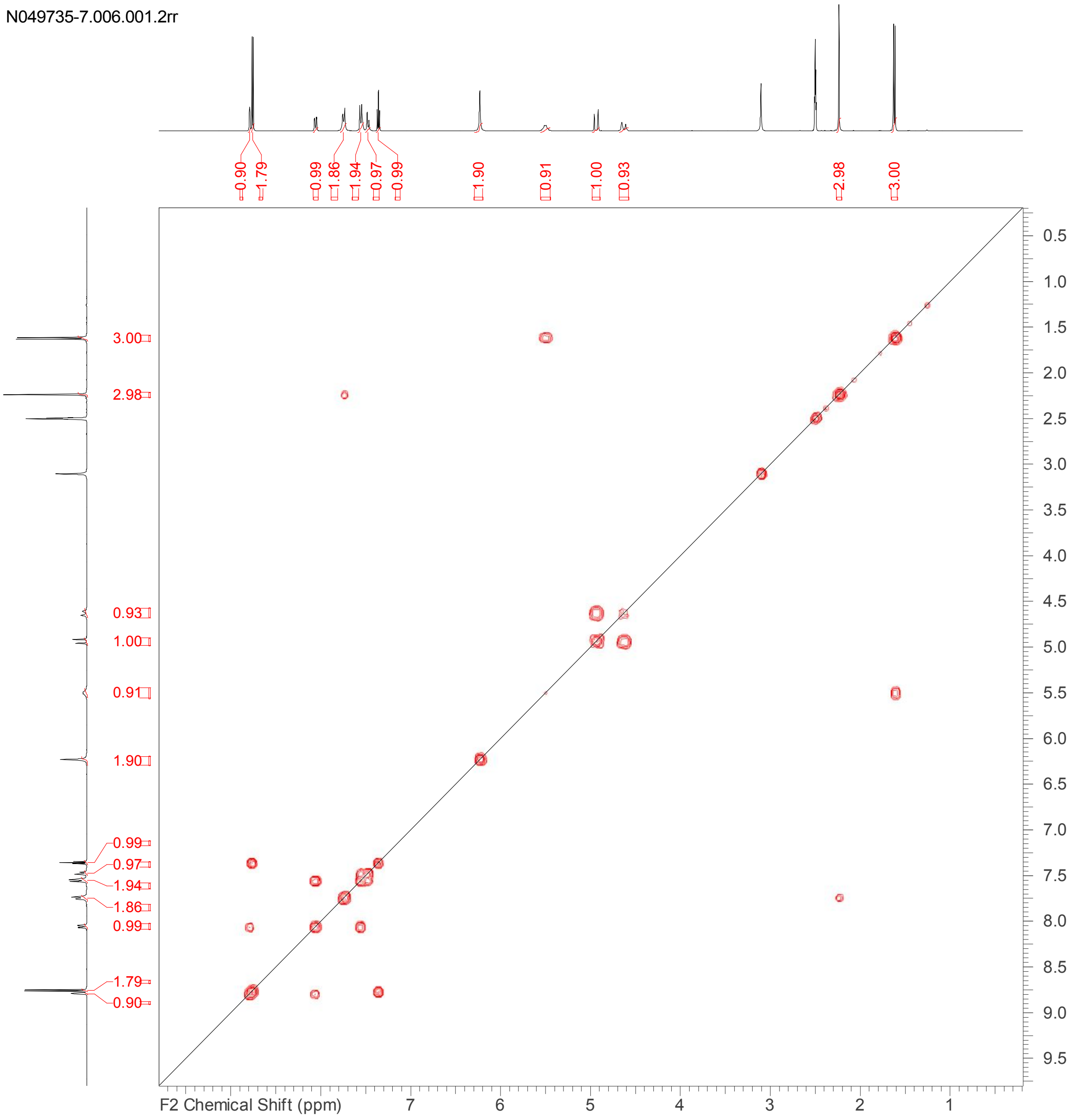

NOESY of AM-9747.

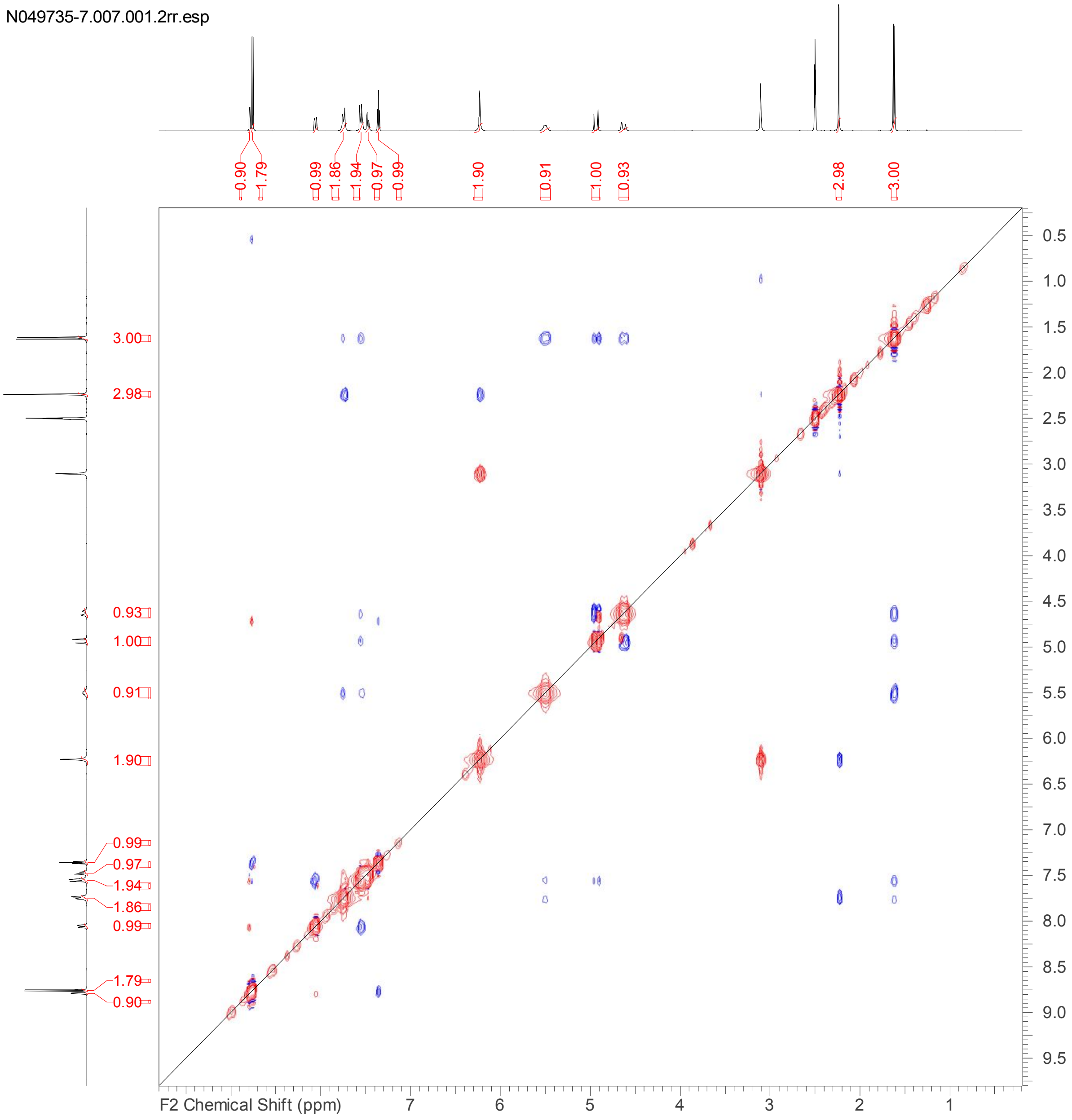

References:

(1) Mavrakis, K. J.; McDonald, E. R., 3rd; Schlabach, M. R.; Billy, E.; Hoffman, G. R.; deWeck, A.; Ruddy, D. A.; Venkatesan, K.; Yu, J.; McAllister, G.; et al. Disordered methionine metabolism in MTAP/CDKN2A-deleted cancers leads to dependence on PRMT5. *Science* **2016**, *351* (6278), 1208-1213. DOI: 10.1126/science.aad5944.

(2) Brehmer, D.; Beke, L.; Wu, T.; Millar, H. J.; Moy, C.; Sun, W.; Mannens, G.; Pande, V.; Boeckx, A.; van Heerde, E.; et al. Discovery and Pharmacological Characterization of JNJ-64619178, a Novel Small-Molecule Inhibitor of PRMT5 with Potent Antitumor Activity. *Mol Cancer Ther* **2021**, *20* (12), 2317-2328. DOI: 10.1158/1535-7163.MCT-21-0367.

(3) Morris, G. M.; Huey, R.; Lindstrom, W.; Sanner, M. F.; Belew, R. K.; Goodsell, D. S.; Olson, A. J. AutoDock4 and AutoDockTools4: Automated docking with selective receptor flexibility. *J Comput Chem* **2009**, *30* (16), 2785-2791. DOI: 10.1002/jcc.21256.

(4) Friesner, R. A.; Murphy, R. B.; Repasky, M. P.; Frye, L. L.; Greenwood, J. R.; Halgren, T. A.; Sanschagrin, P. C.; Mainz, D. T. Extra precision glide: docking and scoring incorporating a model of hydrophobic enclosure for protein-ligand complexes. *J Med Chem* **2006**, *49* (21), 6177-6196. DOI: 10.1021/jm051256o.

(5) Korb, O.; Stutzle, T.; Exner, T. E. Empirical scoring functions for advanced protein-ligand docking with PLANTS. *J Chem Inf Model* **2009**, *49* (1), 84-96. DOI: 10.1021/ci800298z.

(6) Bottegoni, G.; Rocchia, W.; Recanatini, M.; Cavalli, A. AClAP, Autonomous hierarchical agglomerative Cluster Analysis based protocol to partition conformational datasets. *Bioinformatics* **2006**, *22* (14), e58-65. DOI: 10.1093/bioinformatics/btl212.

(7) Team, R. C. *R: A Language and Environment for Statistical Computing*; Computing, R. F. f. S., Ed.; 2022.

(8) *AMBER 2015*; University of California: San Francisco, 2015. (accessed.

(9) Wang, R.; Lai, L.; Wang, S. Further development and validation of empirical scoring functions for structure-based binding affinity prediction. *Journal of Computer-Aided Molecular Design* **2002**, *16* (1), 11-26. DOI: 10.1023/A:1016357811882.

(10) Velec, H. F.; Gohlke, H.; Klebe, G. DrugScore(CSD)-knowledge-based scoring function derived from small molecule crystal data with superior recognition rate of near-native ligand poses and better affinity prediction. *J Med Chem* **2005**, *48* (20), 6296-6303. DOI: 10.1021/jm050436v.

(11) Yang, J. M.; Chen, Y. F.; Shen, T. W.; Kristal, B. S.; Hsu, D. F. Consensus scoring criteria for improving enrichment in virtual screening. *J Chem Inf Model* **2005**, *45* (4), 1134-1146. DOI: 10.1021/ci050034w.

(12) Moretti, L.; Sartori, L. Software Infrastructure for Computer-aided Drug Discovery and Development, a Practical Example with Guidelines. **2016**, *35* (8-9), 382-390. DOI: https://doi.org/10.1002/minf.201501037.

(13) Berthold, M. R.; Cebron, N.; Dill, F.; Gabriel, T. R.; Kötter, T.; Meinl, T.; Ohl, P.; Sieb, C.; Thiel, K.; Wiswedel, B. KNIME: The Konstanz Information Miner. Berlin, Heidelberg, 2008; Springer Berlin Heidelberg: pp 319-326.

(14) Caballero, N. A.; Melendez, F. J.; Munoz-Caro, C.; Nino, A. Theoretical prediction of relative and absolute pKa values of aminopyridines. *Biophys Chem* **2006**, *124* (2), 155-160. DOI: 10.1016/j.bpc.2006.06.007 From NLM Medline.

(15) Sartori, L.; Mercurio, C.; Amigoni, F.; Cappa, A.; Faga, G.; Fattori, R.; Legnaghi, E.; Ciossani, G.; Mattevi, A.; Meroni, G.; et al. Thieno[3,2-b]pyrrole-5-carboxamides as New Reversible Inhibitors of Histone Lysine Demethylase KDM1A/LSD1. Part 1: High-Throughput Screening and Preliminary Exploration. *J Med Chem* **2017**, *60* (5), 1673-1692. DOI: 10.1021/acs.jmedchem.6b01018 From NLM Medline.

(16) Antonysamy, S.; Bonday, Z.; Campbell, R. M.; Doyle, B.; Druzina, Z.; Gheyi, T.; Han, B.; Jungheim, L. N.; Qian, Y.; Rauch, C.; et al. Crystal structure of the human PRMT5:MEP50 complex. *Proc Natl Acad Sci U S A* **2012**, *109* (44), 17960-17965. DOI: 10.1073/pnas.1209814109.

(17) Otwinowski, Z.; Minor, W. Processing of X-ray diffraction data collected in oscillation mode. *Methods Enzymol* **1997**, *276*, 307-326. DOI: 10.1016/S0076-6879(97)76066-X.

(18) McCoy, A. J.; Grosse-Kunstleve, R. W.; Adams, P. D.; Winn, M. D.; Storoni, L. C.; Read, R. J. Phaser crystallographic software. *J Appl Crystallogr* **2007**, *40* (Pt 4), 658-674. DOI: 10.1107/S0021889807021206.
(19) Winn, M. D.; Ballard, C. C.; Cowtan, K. D.; Dodson, E. J.; Emsley, P.; Evans, P. R.; Keegan, R. M.; Krissinel, E. B.; Leslie, A. G.; McCoy, A.; et al. Overview of the CCP4 suite and current developments. *Acta Crystallogr D Biol Crystallogr* **2011**, *67* (Pt 4), 235-242. DOI: 10.1107/S0907444910045749.
(20) Adams, P. D.; Afonine, P. V.; Bunkoczi, G.; Chen, V. B.; Echols, N.; Headd, J. J.; Hung, L. W.; Jain, S.; Kapral, G. J.; Grosse Kunstleve, R. W.; et al. The Phenix software for automated determination of macromolecular structures. *Methods* **2011**, *55* (1), 94-106. DOI: 10.1016/j.ymeth.2011.07.005.
